# A sustainable, efficient, and potentially cost-effective approach to the antimalarial drug candidate MMV688533†

**DOI:** 10.1039/d3sc01699d

**Published:** 2023-05-26

**Authors:** Rahul D. Kavthe, Karthik S. Iyer, Juan C. Caravez, Bruce H. Lipshutz

**Affiliations:** a Department of Chemistry and Biochemistry, University of California Santa Barbara CA 93106 USA lipshutz@chem.ucsb.edu

## Abstract

A 6-step synthesis of the antimalarial drug candidate MMV688533 is reported. Key transformations carried out under aqueous micellar conditions include two Sonogashira couplings and amide bond formation. Compared with the first-generation manufacturing process reported by Sanofi, the current route features ppm levels of palladium loading, less material input, less organic solvent, and no traditional amide coupling reagents. The overall yield is improved ten-fold, from 6.4% to 67%.

## Abstract 2

A 6-step synthesis of the antimalarial drug candidate MMV688533 is reported.

## Graphical abstract

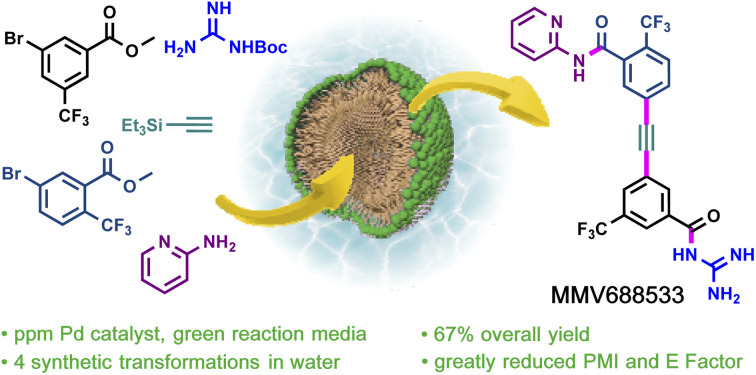

## Introduction

**Fig. 1:**
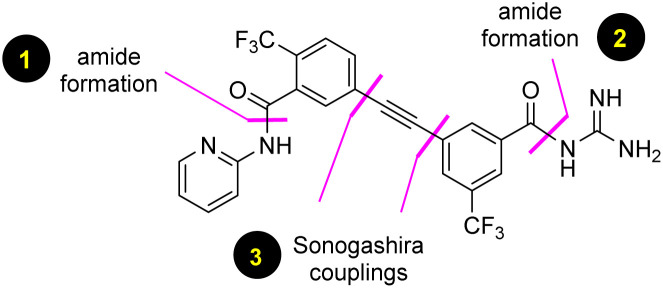
Retrosynthetic analysis of MMV688533 (1).

Malaria is a life-threatening disease that is widely distributed in tropical and subtropical countries, especially in Africa, South Asia, and Latin America. A recent report from the World Health Organization indicated that nearly half of the world's population is at risk, owing to about 247 million cases of malaria having been reported in 2021 alone. Despite the progress made over the last five decades in the treatment of malaria, more than 619 000 deaths were reported in 2021,^1^ most of them being associated with children under five years of age. Since the first antimalarial drug was reported back in 1934, several new and effective treatments have followed, leading to the 2015 Nobel prize in Physiology or Medicine awarded for the development of the antimalarial drug artemisinin.^2^ There are 29 known antimalarial drugs available on the market as part of the fight against malaria;^3^ however, complete eradication of mortality is still an unachieved goal.^4^ Unfortunately, one of the major barriers to controlling global malaria is the emergence of resistant strains of *P. falciparum*.^5^ To address this problem, the Medicines for Malaria Venture (MMV) is actively involved in research and development with private sectors to find highly effective drugs against these persistent strains.^6^ MMV688533 (1) was recently reported as a candidate for advanced clinical development,^7^ and was subsequently featured in C&E News^8^ as a promising single dose treatment for malaria.^9^ Large-scale manufacturing of this drug at the multi-kilogram level for eventual distribution within the third world requires a practical, robust, and environmentally responsible synthesis, which is critical for progression of clinical trials. In this report we describe such a route that, in addition to being the first such alternative synthesis of MMV688533, offers the potential of a cost-effective process that, likewise, is considerably greener than that of prior art (Fig. 1).

Using nanomicelle-forming surfactants as the enabling technology, a wide range of organic reactions can today be successfully carried out in water, including those using substrates and catalysts once thought to be extremely water-sensitive.^10^ This toolbox of established procedures in aqueous micellar media now includes Pd-catalyzed Sonogashira reactions, which rely on ppm levels of palladium loading.^11^ Moreover, conversions, *e.g.*, of complex carboxylic acids and amines to their corresponding amides/peptides can also be accomplished under very green conditions; *i.e.*, using neat conditions, or highly concentrated reaction solutions (2 M in EtOAc), or in an aqueous micellar medium.^12^ Applications of these methodologies towards several drug targets, such as antimalarial agents tafenoquine^13^ and pyronaridine,^15^ as well as the key ingredient in Paxlovid (nirmatrelvir)^14^ all involve sustainable and cost-effective processes. By contrast, routine approaches to these targets typically involve large amounts of traditional and waste-generating organic solvents, as well as high loadings of precious metal catalysts, are verified by comparisons of their calculated *E* Factors or PMI values. These unwanted features not only add to the cost of manufacturing, but also place a substantial burden on our petroleum reserves and precious metal usage, in addition to the serious risks to the health and safety of workers.^16^ With these factors in mind, we set out to develop a streamlined, green, and an economically attractive route to this potentially important antimalarial drug candidate MMV688533.

**Scheme 1:**
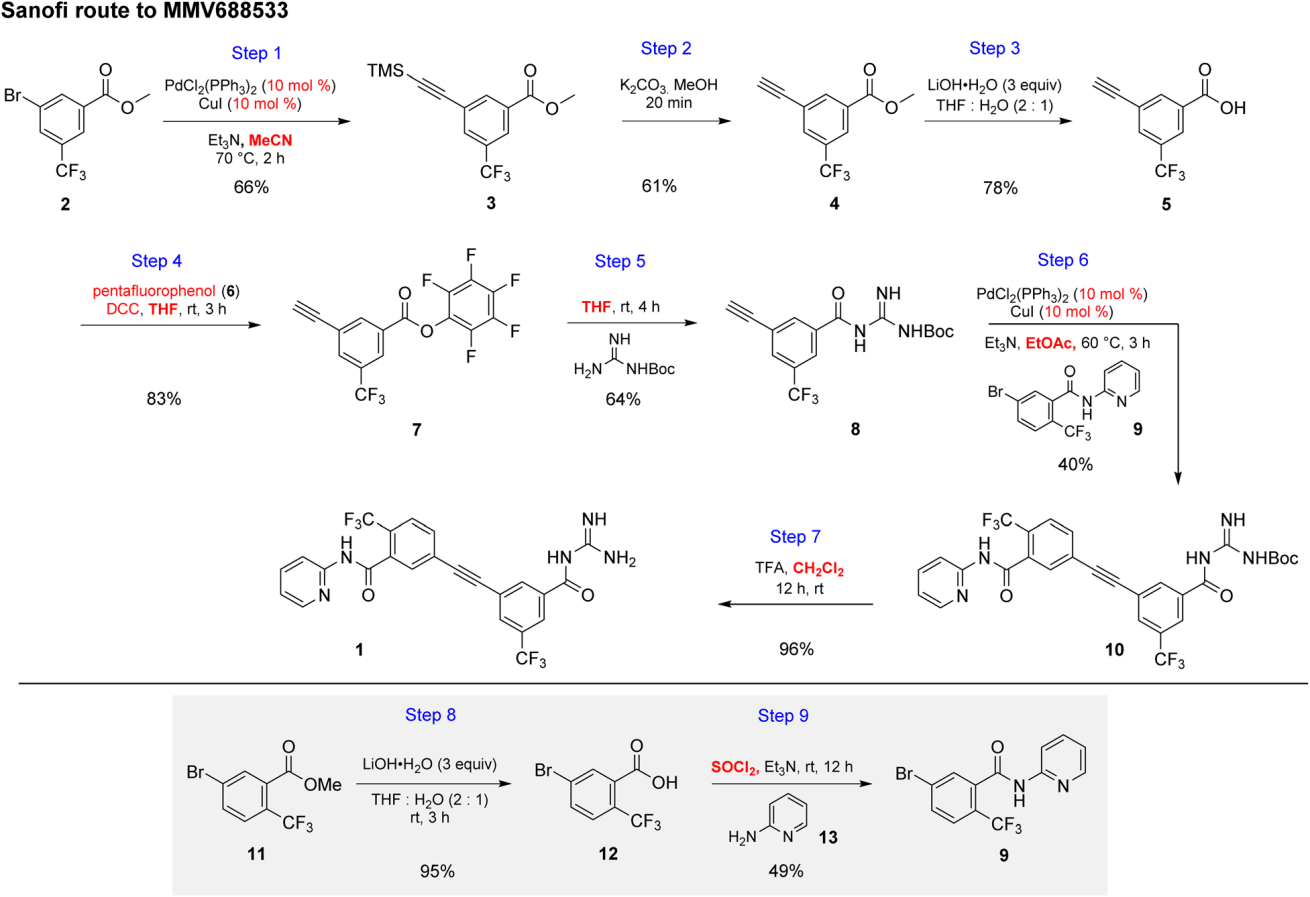
Discovery route used by Sanofi to MMV688533 (1).

The initial discovery route to MMV688533 reported by Sanofi to arrive at 1 is shown in Scheme 1,^6^ where the overall yield was 6.4% based on the longest linear sequence. There are several other major drawbacks associated with this sequence, including (a) a very high loading of palladium catalyst (10 mol%) used in both steps 1 and 6 for the Sonogashira couplings; (b) an additional ester hydrolysis (step 3) resulting from the ineffectiveness of the Sonogashira coupling when run in the presence of the required carboxylic acid; (c) the need for waste-generating traditional amide coupling reagents, such as DCC (step 4); (d) isolation of intermediate pentafluorophenyl ester 7 (step 4); (e) use of the weakly nucleophilic 2-aminopyridine 13 leading to a low yield of the desired amide 9 (step 9); and (f) a required additional ester hydrolysis step (step 8). Thus, numerous opportunities for improvement were envisioned, while maximizing time^17^ and pot economies.^18^

## Results and discussion

### Synthesis of intermediate 14

To avoid the additional esterification and hydrolysis steps used previously,^6^ amidation was envisioned prior to the first required Sonogashira coupling. Thus, an initial coupling between acid 13 and *N*-Boc guanidine to form amide 14 was pursued based on our newly developed thioester-based technology to generate amide bonds under a variety of green conditions that avoids traditional coupling reagents (Table 1).^12^ Several classes of activating agents,^19^ such as DPDTC, COMU,^20^ EDCI/HOBt^21^ and DCC^22^ were screened for comparison purposes under aqueous micellar conditions. Use of dipyridyldithiocarbonate (DPDTC), together with *N*-methylmorpholine (NMM) as base led to only 73% yield of product 14 (entry 1). The addition of catalytic amounts of DMAP (10 mol%) increased the yield to 85% (entry 2). While traditional coupling reagents led to poor results (entries 3–5), readily available activating agents, including cyanuric chloride (TCT)^23^ and thionyl chloride (SOCl_2_),^24^ were also screened thereby furnishing the corresponding amide, albeit in low yields (entries 6 and 7). To further evaluate the nucleophilicity of *N*-Boc guanidine as the amine coupling partner, reactions were carried out in organic solvents such as DMF and EtOAc using well-known coupling reagents T3P and EDCI/HOBt. Interestingly, these reactions gave lower yields of 14 (entries 8 and 9). By contrast, coupling with DPDTC under neat conditions^12^ (at 60 °C) in a 2-step, 1-pot fashion (formation of the thioester intermediate followed by addition of the amine) furnished amide 14 in 96% isolated yield (entry 10). A similar outcome was observed by running the reaction in highly concentrated (2 M) EtOAc (entry 11). One advantage of using DPDTC to form *in situ* the corresponding thioester include the recyclability and ease of removal of (odorless) by-product 2-mercaptopyridine *via* in-flask extraction with controlled amounts of aqueous hydroxide (1 M). Moreover, the same base washing serves to simultaneously remove traces of unreacted acid starting material.

**Table 1:** Optimization of reaction conditions for the preparation of amide 14

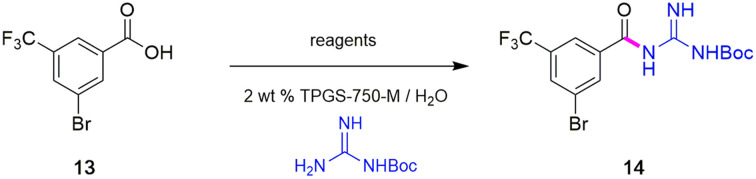					
Entry	Activating agent	Time (*t*)	Temp (°C)	Base	Yield^a^ (%)
1	DPDTC (1.05 equiv.)	8	60	NMM	73
2^c^	DPDTC (1.05 equiv.)	4	60	NMM	85
3	COMU (1.05 equiv.)	8	30	2,6-Lutidine	—b
4	EDCI·HCl (1.1 equiv.) HOBt (1.2 equiv.)	18	45	—	45
5	DCC (1.5 equiv.)	18	45	—	10
6	Cyanuric chloride (1 equiv.)	8	rt	NMM	Trace
7	Thionyl chloride (7 equiv.)	8	50	K_2_CO_3_	Trace
8^c^^,^^d^	EDCI·HCl (1.1 equiv) HOBt (1.2 equiv.)	16	rt	—	61
9^e^	T3P	8	rt	DIPEA	27
**10^c^** ^**,**^ **f**	**DPDTC (1.05 equiv.)**	**8**	**60**	**NMM**	**96**
11^a^	DPDTC (1.05 equiv.)	8	60	NMM	94
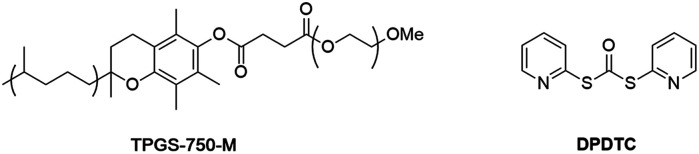					

a Isolated yield.

b Multiple spots observed on TLC.

c 10 mol% DMAP used.

d Run in DMF.

e Run in EtOAc.

f Run in the absence of solvent (neat).

### Sonogashira couplings to afford alkynes 15 and 16

The two required Sonogashira cross couplings are, perhaps, the most crucial and likely the most cost-intensive steps in the synthesis of MMV688533. A recent literature report^6^ describing the first of these two reactions involves use 10 mol% of a Pd catalyst, along with 10 mol% CuI together in MeCN affording the coupled alkyne in 66% yield (educt 2 going to product 3 in Scheme 1). Earlier studies from our group had shown that ppm Pd-catalyzed Sonogashira reactions could be effected in aqueous nanoreactors;^11^ hence; a far more economically attractive process for this coupling was pursued. Initially, the reaction between aryl bromide 14 with TES-acetylene was carried out using 1 mol% Pd[(cinnamyl)Cl]_2_ together with Takasago's ligand cBRIDP (2 mol%) to give 15, but in only 2% NMR yield (Table 2, entry 1).^11*a*^ After screening various Pd catalysts, it was observed that relatively inexpensive Pd(PPh_3_)_4_ (2.5 mol%) along with CuI (1 mol%) was the most effective system, leading to alkyne 15 in 91% yield (entry 9). All attempts to lower the amount of Pd by screening several palladacycles^25^ with various ligands (entries 11–14) gave non-competitive results. Likewise, nanoparticles derived from FeCl_3_ doped with Pd(OAc)_2_ (1 mol%) and XPhos (2 mol%) also resulted in a lower yield of product (entry 7).^11*b*^

**Table 2:** Optimization of the Sonogashira coupling to arrive at 15

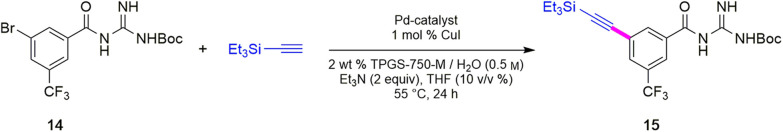		
Entry^a^	Pd catalyst	Yield^b^ (%)
1	[Pd(cinnamyl)Cl]_2_ (1 mol% total Pd)	2
	cBRIDP (2 mol%)	
2	Pd(dppf)Cl_2_·DCM (1 mol%)	68
3	Pd(dtbpf)Cl_2_ (1 mol%)	20
4	PdCl_2_(PPh_3_)_2_ (1 mol%)	41
5	Xantphos Pd G4 (1 mol%)	61
6	SPhos Pd G3 (1 mol%)	Trace
7	FeNP (3 mol%), Pd(OAc)_2_ (1 mol%), XPhos (2 mol%)	15
8	Pd(Ph_3_P)_4_ (1 mol%)	60
**9^c^**	**Pd(Ph**_**3**_ **P)**_**4**_ **(2.5 mol%)**	**91 (88)^e^**
10^d^	Pd(dppf)Cl_2_·DCM (1 mol%)	84
11^d^	BrettPhos Pd G3	12
12^d^	*N*-XantPhos Pd G3	74
13	P1	48
14	P2	Trace
15	P3	50
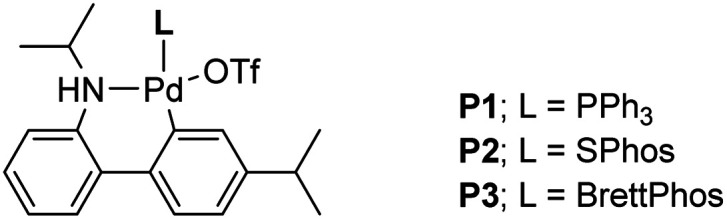		

a Reactions were carried out on a 0.25 mmol scale.

b NMR yields using 1,3,5-trimethoxybenzene as internal standard.

c 5 mol% CuI was used.

d 4 mol% CuI was used.

e Isolated yield.

Although the Sonogashira coupling to arrive at 15 using catalyst Pd(PPh_3_)_4_ appeared promising, the required high loading of Pd (2.5 mol%) was not a sufficient improvement in our opinion over the established route (which uses 10 mol%). Thus, an alternative sequence requiring far less Pd was investigated. We hypothesized that Ph_3_P was not sufficiently bulky to fully prevent nitrogen in the guanidine moiety from coordinating to palladium, thereby increasing the amount of catalyst needed. By carrying out a Sonogashira coupling before inserting the guanidine moiety, on the other hand, resulted in clean coupling of methyl ester 2 to give the initially targeted alkyne 16 (Table 3). Full conversion occurred within 16 hours at 70 °C, producing 16 in 95% yield using only 0.5 mol% of total Pd-catalyst, and in the complete absence of copper (entry 1). Further screening of catalyst loading revealed that similar reaction efficiency could be achieved with only 2500 ppm (0.25 mol%) of the total Pd catalyst and 5000 ppm (0.5 mol%) of cBRIDP ligand (entry 3). This protocol has been reproduced on a larger scale (5 mmol) as well (see ESI, Section 3.3 and Scheme S3†).

**Table 3:** Optimization of reaction conditions for Sonogashira coupling

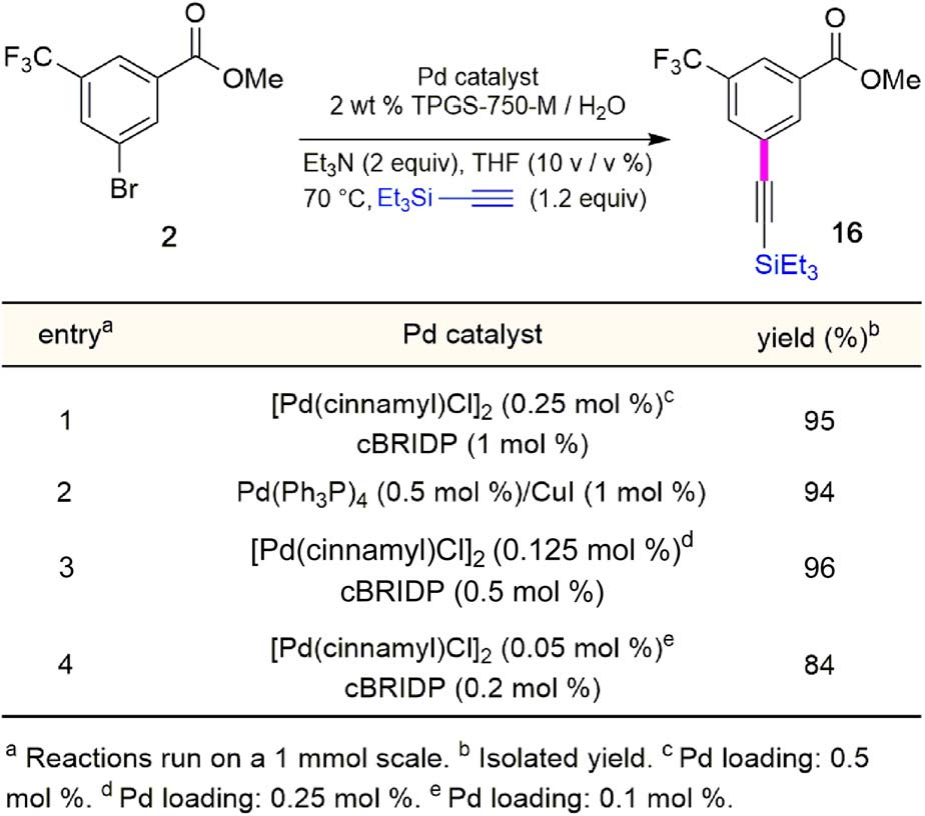

### Synthesis of intermediate 5

**Scheme 2:**
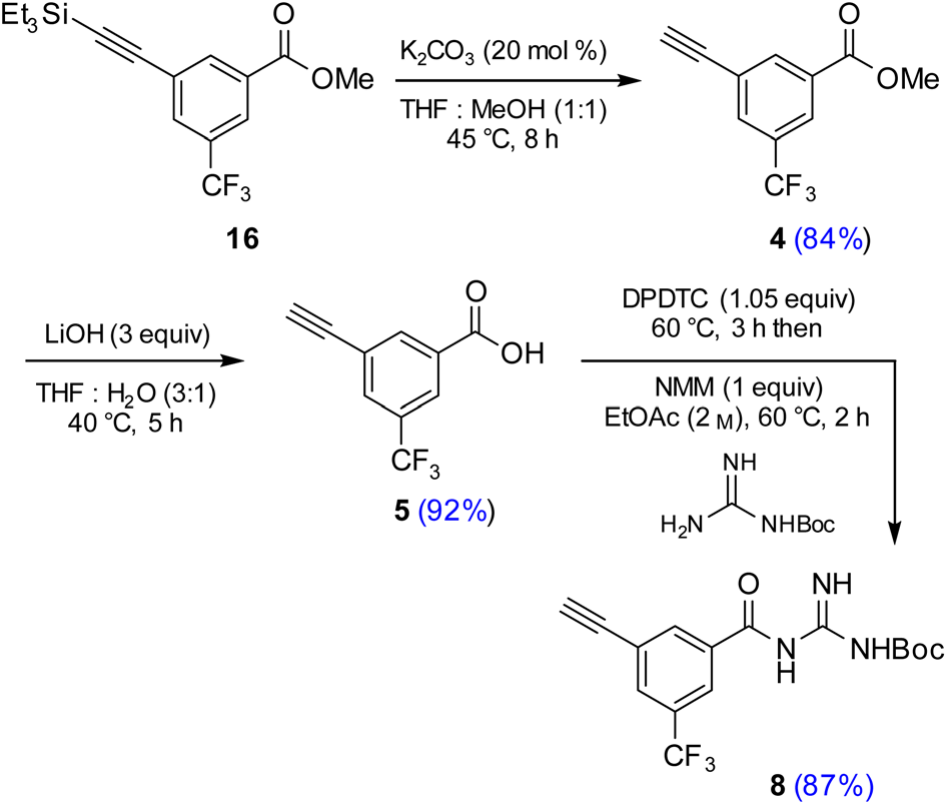
Sequence used to prepare terminal alkyne 8.

Attempts were then made to prepare carboxylic acid 5 (see Scheme 4) directly from intermediate 16*via* a simultaneous one-pot TES deprotection/ester hydrolysis using LiOH in aqueous THF. Unfortunately, the results were not encouraging, as a significant amount of the TES group was still present in the reaction mixture. Hence, a sequential two-step TES deprotection/ester hydrolysis approach was developed (Scheme 2). Desilylation of the TES residue in 16 to afford terminal alkyne 4 was carried out using catalytic amounts of K_2_CO_3_ in a mixture of THF and MeOH (1 : 1),^11^ since desilylation in an aqueous medium led to no reaction. The resulting methyl ester 4 was then hydrolyzed using LiOH in aqueous THF to afford carboxylic acid 5. Once optimized conditions associated with each step had been determined, a two-step, one-pot synthesis was developed starting with 16 and ultimately affording 5 in 89% overall isolated yield, following an aqueous acidic wash. No column chromatography was needed at any stage for purification (Scheme 4, steps 2 and 3).

### Synthesis of terminal alkyne 8 from acid 5

Having established conditions leading to amide bond formation associated with compound 14 (see Table 1, entry 10), identical reaction conditions were utilized in the preparation of amide 8. The reaction of carboxylic acid 5 with DPDTC neat at 60 °C afforded the corresponding thioester, to which was then added Boc-guanidine and NMM in concentrated EtOAc (2 M) at 60 °C affording 8 in 87% isolated yield. Once optimized conditions associated with each step had been determined, a four-step, one-pot synthesis was developed starting with 2 and ultimately affording 8 in 61% overall isolated yield (see ESI, Scheme S8†).

### Synthesis of intermediate 9*via* direct amidation

**Scheme 3:**
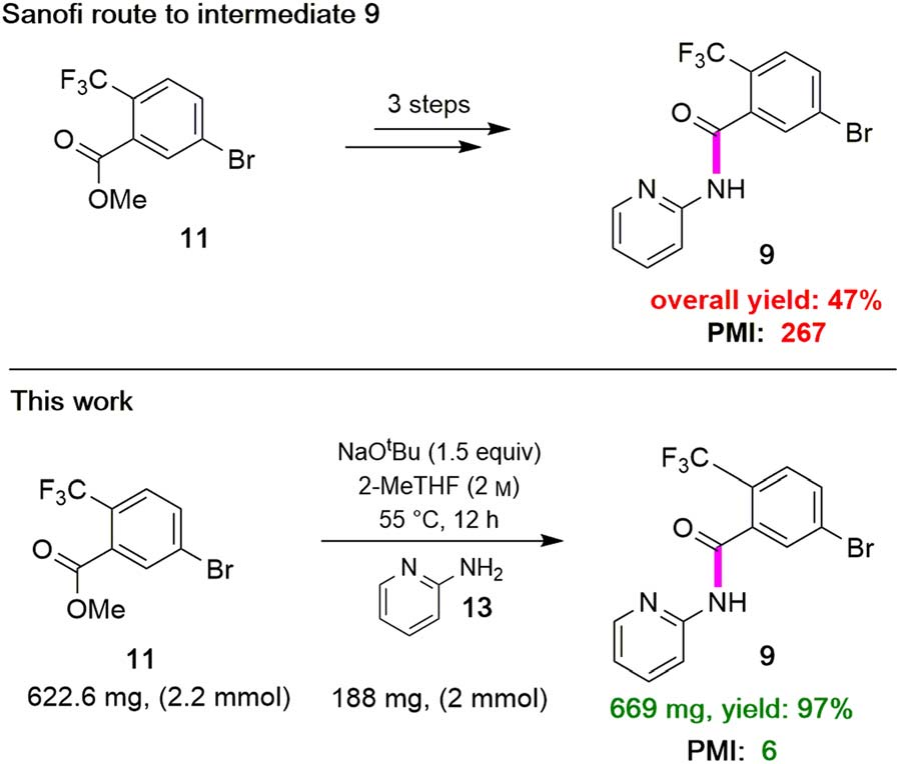
Sequence en route to intermediate 9.

Attention was next focused on assembly of the second fragment that also involved amide bond formation. Following a recently disclosed procedure by Zhang and co-workers,^26^ intermediate 9 can be constructed *via* amidation between unactivated ester 11 and 2-aminopyridine 13. Initial attempts using NaO^*t*^Bu as base at room temperature under aqueous micellar conditions resulted in only a trace amount of the desired product 9, along with 94% recovered starting material (Table 4, entry 1). Further attempts to perform the reaction in water with an increased reaction time or at a higher temperature showed little improvement (entry 2). When the reaction was performed neat at room temperature for 2 h, a 59% yield of product resulted (entry 3). Increasing the reaction time to six hours increased the yield to 66% (entry 4). A significant improvement was eventually observed when the temperature was increased to 60 °C (entry 5). The use of 1.5 equivalents of ester 11 is necessary to achieve this higher yield, which also aids in stirring the reaction mixture. When the reaction was carried out in 2-MeTHF at 55 °C, amide 9 was isolated in 95% yield (entry 6). Furthermore, decreasing the stoichiometry of 11 from 1.5 to 1.2 or 1.1 equivalents gave rise to comparable yields of 9 (entries 7 and 8 *vs.* entry 6). However, couplings using DPDTC, HATU, or T3P reagents gave poor results under aqueous conditions, while their use in organic solvents led to very modest results (<46% yield) presumably due to the weakly nucleophilic nature^27^ of 2- aminopyridine in this type of medium (see ESI, Section 3.5 and Table S4†). The scalability of this protocol at 2.2 mmol was demonstrated without loss in reaction yield (Scheme 3b). It is noteworthy that 9 can now be prepared in a single step featuring an associated low PMI (*i.e.*, 6), compared to the existing three-step process with a much higher PMI (*i.e.*, 267; Scheme 3).

**Table 4:** Optimization of reaction conditions for direct amidation of ester 11

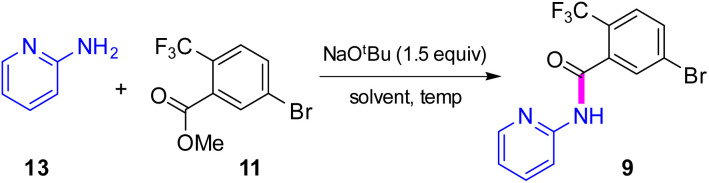				
Entry^a^	Solvent	Temp	Time	Yield^b^
1	2 wt% TPGS-750-M/H_2_O	rt	16	—c
2	2 wt% TPGS-750-M/H_2_O	80	16	15
3	Neat	rt	2	59
4	Neat	rt	6	66
**5**	**Neat**	**60**	**4**	**96**
6^d^	2-MeTHF	55	14	95
7^d^^,^^e^	2-MeTHF	55	14	98
**8^d^** ^**,**^ **f**	**2-MeTHF**	**55**	**14**	**>99**
9^f^^,^^g^	2-MeTHF	55	14	87
10^g^	Neat	55	14	>99

a Reaction conditions: 13 (0.25 mmol), 11 (0.37 mmol), solvent (0.5 M).

b Isolated yield.

c 94% starting material remained unreacted.

d Solvent (2 M) was used.

e 11 (0.3 mmol) was used.

f 11 (0.27 mmol) was used.

g KO^*t*^Bu was used instead of NaO^*t*^Bu.

**Scheme 4:**
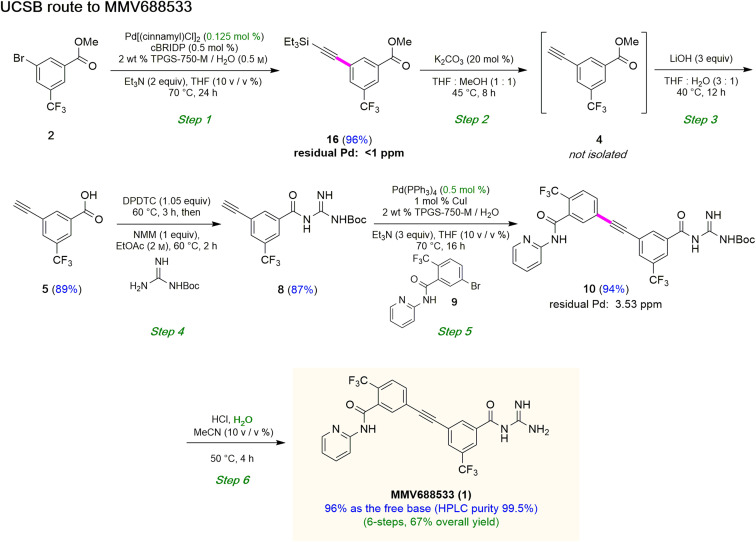
Overall route to MMV688533.

### Sonogashira coupling and *N*-Boc deprotection to arrive at MMV688533 (1)

The combination of 8 and 9 was screened in terms of catalysts leading to alkyne 10 (Table 5, entries 1–11). Ultimately, it was observed that using only 5000 ppm (0.5 mol%) Pd(PPh_3_)_4_ in aqueous micellar media at 55 °C led to the desired coupling product 10 in 94% isolated yield (entry 7). Further reduction in catalyst loading to 2500 ppm (0.25 mol%) afforded 10 in only 68% yield.

**Table 5:** Optimization of reaction conditions for the second Sonogashira coupling^a^

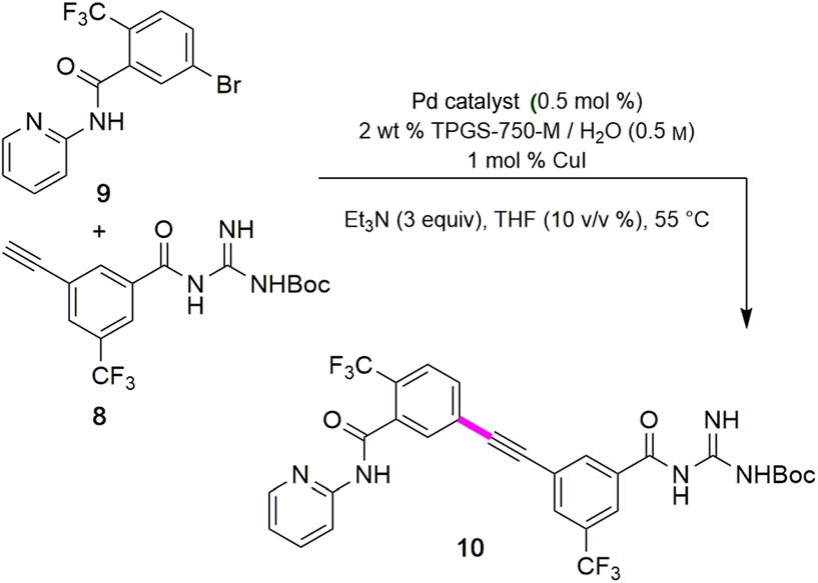			
Entry	Pd catalyst	Time (*t*)	Yield^b^ (%)
1^c^	Pd[(cinnamyl)Cl]_2_ cBRIDP (1 mol %)	24	19
2	Pd(PPh_3_)Cl_2_	24	47
3	Pd(dppf)Cl_2_·DCM	24	29
4	Pd(dtbpf)Cl_2_	24	51
5	XantPhos Pd G4	24	93
6	Pd(Ph_3_P)_4_	24	96
**7^d^**	**Pd(Ph**_**3**_ **P)**_**4**_	**16**	**94**
8^e^	Pd(Ph_3_P)_4_	24	68
9^f^	Pd(Ph_3_P)_4_	16	87
10	PdCl_2_(CH_3_CN)_2_	24	34
11^g^	Pd(Ph_3_)_2_Cl_2_	24	30

a Reaction condition: 9 (0.25 mmol), 8 (0.30 mmol), Et_3_N (3 equiv.), solvent (0.5 M).

b Isolated yield.

c Run in the absence of CuI.

d Run on 0.5 mmol scale.

e Pd(Ph_3_P)_4_ (2500 ppm) used.

f Run in the absence of co-solvent.

g Run in EtOAc (0.5 M).

**Fig. 2:**
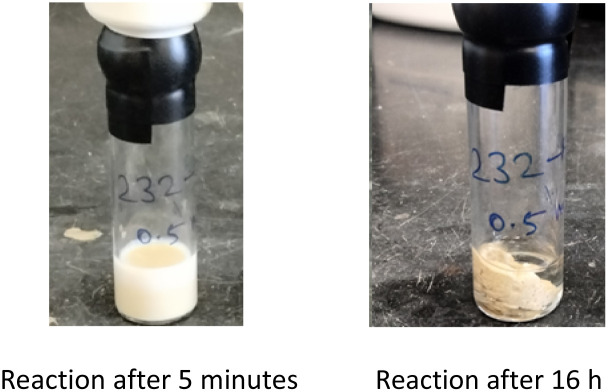
Appearance of Sonogashira coupling between 8 and 9 using 0.5 mol% Pd(PPh_3_)_4_.

Interestingly, the corresponding reaction profile in EtOAc was not clean, giving the desired product in only 30% yield (entry 11). Upon completion, 10 precipitates from the reaction mixture and is isolated simply by decanting off the aqueous layer (Fig. 2). However, the same reaction in EtOAc appeared to be low yielding (entry 11), with the crude product containing highly-colored impurities. Hence, running the reaction in aqueous TPGS-750-M not only allows for a significant reduction in precious metal catalyst loading, but also maximizes pot economy which can otherwise be costly at scale. Subsequent removal of the *N*-Boc protecting group was achieved using 5 equiv of aqueous HCl at 50 °C to afford 1 in 96% yield.^28^ The 10 v/v % acetonitrile co-solvent was included to help with reaction stirring. Once the deprotection is complete, addition of aqueous sodium hydroxide leads to precipitation of product 1, which is directly isolated *via* centrifugation in 96% yield. It should be noted that *N*-Boc deprotection in organic solvent is very slow, with only trace amounts of desired product being formed. However, use of 20 equiv of TFA under neat conditions gave the desired product in 89% yield (see ESI, Section 3.7 and Table S6†).

**Fig. 3:**
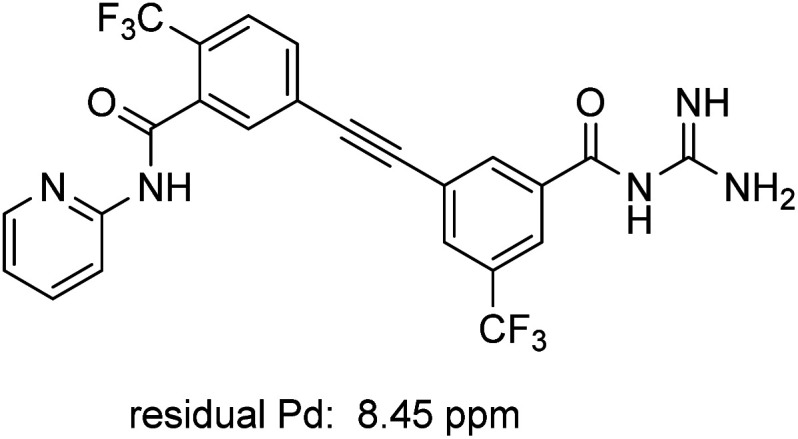
Residual palladium in newly prepared MMV688533 (1).

ICP-MS analysis of compound 1 revealed low levels of residual metal: only 8.45 ppm of Pd, which is below the FDA allowed 10 ppm per day per dose (Fig. 3).^29^ Similar analysis of intermediate 10 prepared *via* literature methods (*i.e.*, 10 mol% PdCl_2_(PPh_3_)_2_ and 10 mol% CuI) showed the presence of *ca.* 3800 ppm Pd (see ESI, Section 5†), thereby necessitating further processing to remove excess Pd.

**Fig. 4:**
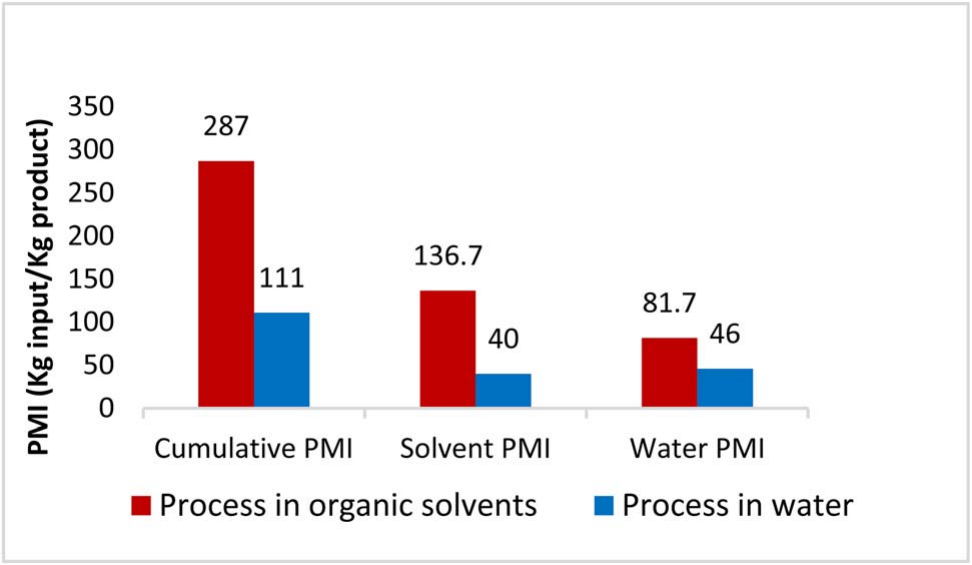
Comparison of PMI between the literature process in organic solvent with this process in water.

To verify greenness and extent of sustainability of the present process, quantitative green metrics, including a complete *E* Factor (cEF)^30^ and process mass intensity (PMI)^31^ were evaluated (see ESI, Section 4†). These were calculated and compared based on the Sanofi process (as detailed in ESI†). The results are shown in Fig. 4 and indicate that the route utilizing chemistry in water leads to a net decrease in process mass intensity from 287 to 111, representing a 61% reduction in materials required to manufacture MMV688533. Furthermore, Table 6 provides a comparison between this work and Sanofi's discovery route^6^ for several of the key steps. It features an improvement in environmental impact as exemplified by the number of steps, along with a 10-fold increase in overall yield.

**Table 6:** Comparisons between route by Sanofi and this work en route to MMV688533 (1)

Reaction parameter	Sanofi	This work
1st Sonogashira coupling	10 mol% Pd, MeCN	0.25 mol% Pd, H_2_O
Amidation to afford 8	DCC, THF, pentafluorophenol	DPDTC, neat EtOAc (2 M)
Synthesis of 9	SOCl_2_, pyridine	NaO^*t*^Bu, neat or 2-MeTHF (2 M)
Sonogashira coupling of 8 and 9	10 mol% Pd, EtOAc	0.5 mol% Pd, H_2_O
*N*-Boc deprotection	TFA, DCM	aq. HCl, H_2_O
*E* Factor (all waste)	286	110
Number of steps for synthesis of 1	7	6 steps, 5 pots
Overall yield	6.4%	67% (*via* int-16)
		64% (*via* int-14)^a^

a See ESI.

## Conclusions

A highly efficient and sustainable route to the antimalarial drug MMV688533 has been developed. All reactions can be performed under neat or aqueous micellar conditions, or in a green solvent. The key Sonogashira reactions take place in water, enabled by nanoreactors derived from the benign-by-design surfactant TPGS-750-M. Although highly functionalized intermediates are involved, only ppm levels of a commercially available and relatively inexpensive Pd catalyst are needed. The first amide bond formation was achieved using our newly introduced, green, practical, and user-friendly protocol leading to the targeted amides in good-to-excellent yields under very mild conditions. And second amide bond formation with less nucleophilic amine was done using a NaO^*t*^Bu-promoted protocol. The present route may significantly reduce both the cost and environmental footprint associated with this antimalarial drug candidate, thereby making it potentially more available to a wider group of recipients, especially in third world countries.

## Data availability

The synthetic procedures, characterization, and spectral data supporting this article have been uploaded as part of the ESI.†

## Author contributions

R. D. K. conceived the project and drafted the initial manuscript. K. S. I. and J. C. C. performed experiments and contributed to the manuscript. B. H. L. oversaw the work and aided in drafting the final manuscript.

## Conflicts of interest

There are no conflicts to declare.

## Supplementary Material

SC-014-D3SC01699D-s001
