# Supplementary material for: A sustainable, efficient, and potentially cost-effective approach to the antimalarial drug candidate MMV688533
Source: Chem Sci. 2023 May 26;14(23):6399–407. doi: 10.1039/d3sc01699d (PMC10266478; doi:10.1039/d3sc01699d)
Supplement: SC-014-D3SC01699D-s001 [file SC-014-D3SC01699D-s001.pdf]

Electronic Supplementary Material (ESI) for Chemical Science.
This journal is © The Royal Society of Chemistry 2023

Electronic Supplementary Information

A Sustainable, Efficient, and Potentially Cost-effective Approach to the Antimalarial Drug MMV688533

Rahul D. Kavthe, Karthik S. Iyer, Juan C. Caravez, and Bruce H. Lipshutz*

Department of Chemistry & Biochemistry, University of California, Santa Barbara, CA 93106 USA

Phone: 805-893-2521
Fax: 805-893-8265
Email: lipshutz@chem.ucsb.edu
website: https://lipshutz.chem.ucsb.edu/

Contents

1. General information 2
2. Synthetic schemes 4
3. General procedures 6
 3.1 Optimization for amide bond formation 6
 3.2 Optimization for Sonogashira coupling 8
 3.3 Optimization for synthesis of Int-**16** 10
 3.4 Synthesis of Int-**8** 12
 3.4 Synthesis of **9** from methyl 5-bromo-2-(trifluoromethyl)benzoate 17
 3.5 Synthesis of **9** from carboxylic acid **11** 18
 3.6 Synthesis of **10** 20
 3.7 *N*-Boc deprotection to afford MMV688533 (**1**) 21
4. PMI and E Factor calculations 22
5. ICP-MS data for residual palladium 32
6. Analytical data 33
7. References 36
8. HPLC data 37
9. NMR spectral data 38

1. General information

Reagents:

Reagents were purchased from Sigma-Aldrich, Combi-Blocks, TCI America, Inc., Ambeed, Inc., BLD Pharma, Fischer Scientific, and used without further purification unless noted otherwise.

Surfactant Solution Preparation:

The surfactant, TPGS-750-M, was prepared via a standard literature procedure,[1] or can be purchased from Millipore-Sigma (catalog #733857 as a 2 wt % solution of the wax dissolved in water). A standard 2 wt % aqueous solution of TPGS-750-M was typically prepared on a 100 g scale by dissolving 2 g of the wax in 98 g of thoroughly degassed (steady stream of argon, minimum of 1 h bubbling time with stirring) HPLC grade water in a 250 mL round-bottomed flask equipped with a stir bar and allowed to stir vigorously overnight under argon (NOTE: Do not attempt to degas the aqueous phase with surfactant wax submerged; vigorous foaming to the point of overflowing may occur). The 2 wt % TPGS-750-M/H_2O solution, once prepared, was kept under a positive argon pressure at all times.

Chromatography:

Silica gel TLC plates (UV 254 indicator, thickness 200 mm standard grade, glass backed and 230-400 mesh from Merck) were used. The developed TLC plate was analyzed by a UV lamp (254 nm). The plates were further analyzed with the use of an aqueous ceric ammonium molybdate stain or permanganate stain and developed with a heat gun. Flash chromatography was performed using Silicycle Silicaflash® P60 unbonded grade silica.

Nuclear Magnetic Resonance Spectroscopy (NMR):

^1H, ^{13}C, and ^{19}F NMR were recorded at 25 ºC on an Agilent Technologies 400 MHz, a Bruker Avance III HD 400 MHz and Bruker Avance NEO 500 MHz spectrometer in $CDCl_3$ or DMSO-d_6 with residual $CHCl_3$ (^1H = 7.26 ppm, ^{13}C = 77.16 ppm) or DMSO (^1H = 2.54 ppm, ^{13}C = 40.45 ppm) as the internal standard. Deuterated solvents were purchased from Cambridge Isotope Laboratories. Chemical shifts are reported in parts per million (ppm). The data presented will be reported as follows; chemical shift, multiplicity (s = singlet, bs = broad singlet, d = doublet, dd =

doublet of doublet, t = triplet, q = quartet, quin = quintet, m = multiplet), coupling constant (if applicable), and integration.

Mass Spectrometry (MS):

HRMS analysis (ESI-MS or CI-MS) was performed by the UC Santa Barbara mass spectrometry facility or the UC Irvine mass spectrometry facility. ESI-MS analysis was performed on a Waters LCT Premier mass spectrometer equipped with an Alliance 2695 Separations module. EI-MS analysis was performed on a Waters GCT Premier mass spectrometer equipped with an Agilent 7890A GC oven and J&W Scientific DB-5ms+DG narrow bore column using helium carrier gas.

Melting points (MP):

Melting points were determined using a MEL-TEMP II melting point apparatus with samples in Kimble Kimex 51 capillaries (1.5-1.8 x 90 mm).

High-Performance Liquid Chromatography (HPLC):

HPLC analysis was performed on an Agilent 1220 series HPLC with columns indicated for each compound. HPLC-grade solvents were obtained from Fischer Scientific. Column conditions are specified for each relevant substrate.

Inductively coupled plasma mass spectrometry (ICP-MS):

ICP-MS analysis was performed using a PerkinElmer NexION 2000 ICP-MS.

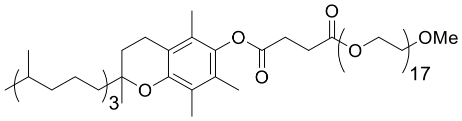

TPGS-750-M

2. Synthetic schemes

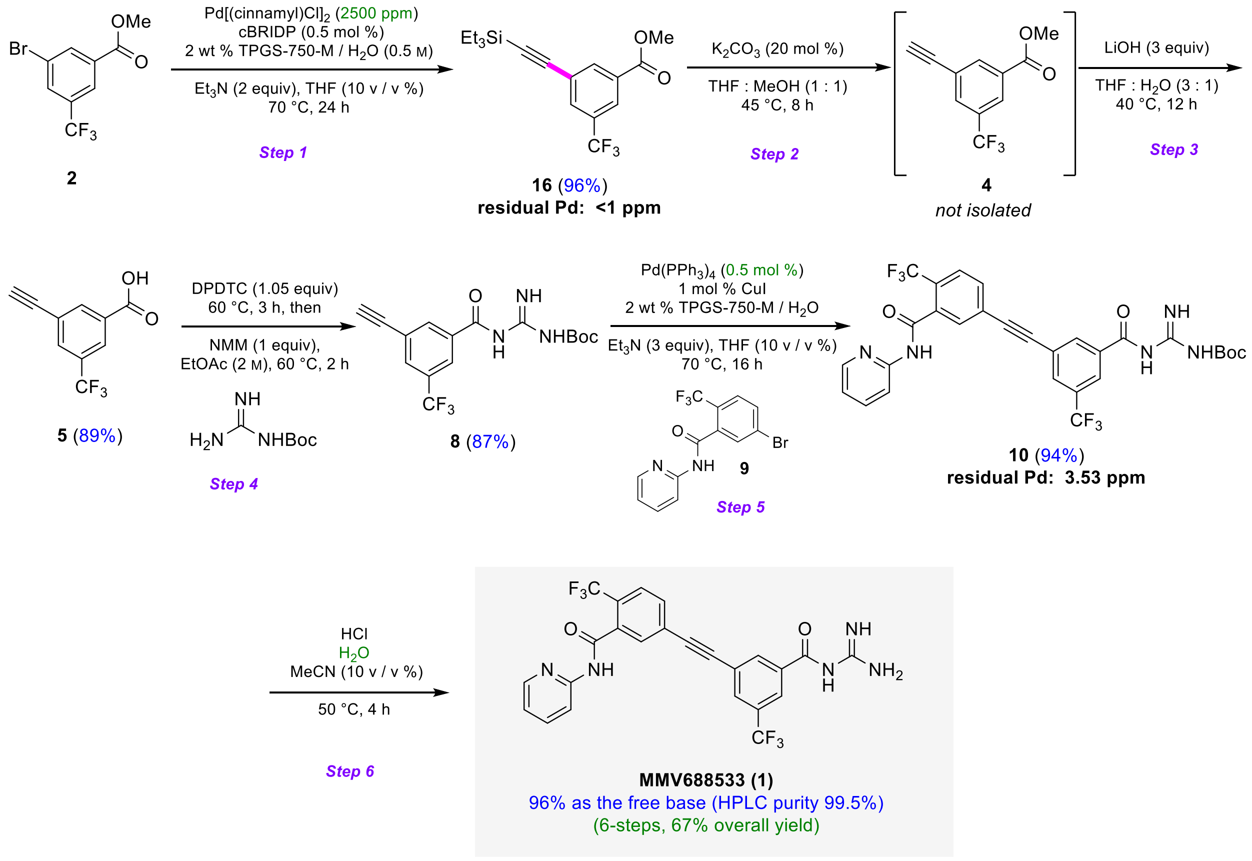

Scheme S1: UCSB synthesis of MMV688533 via Route 1.

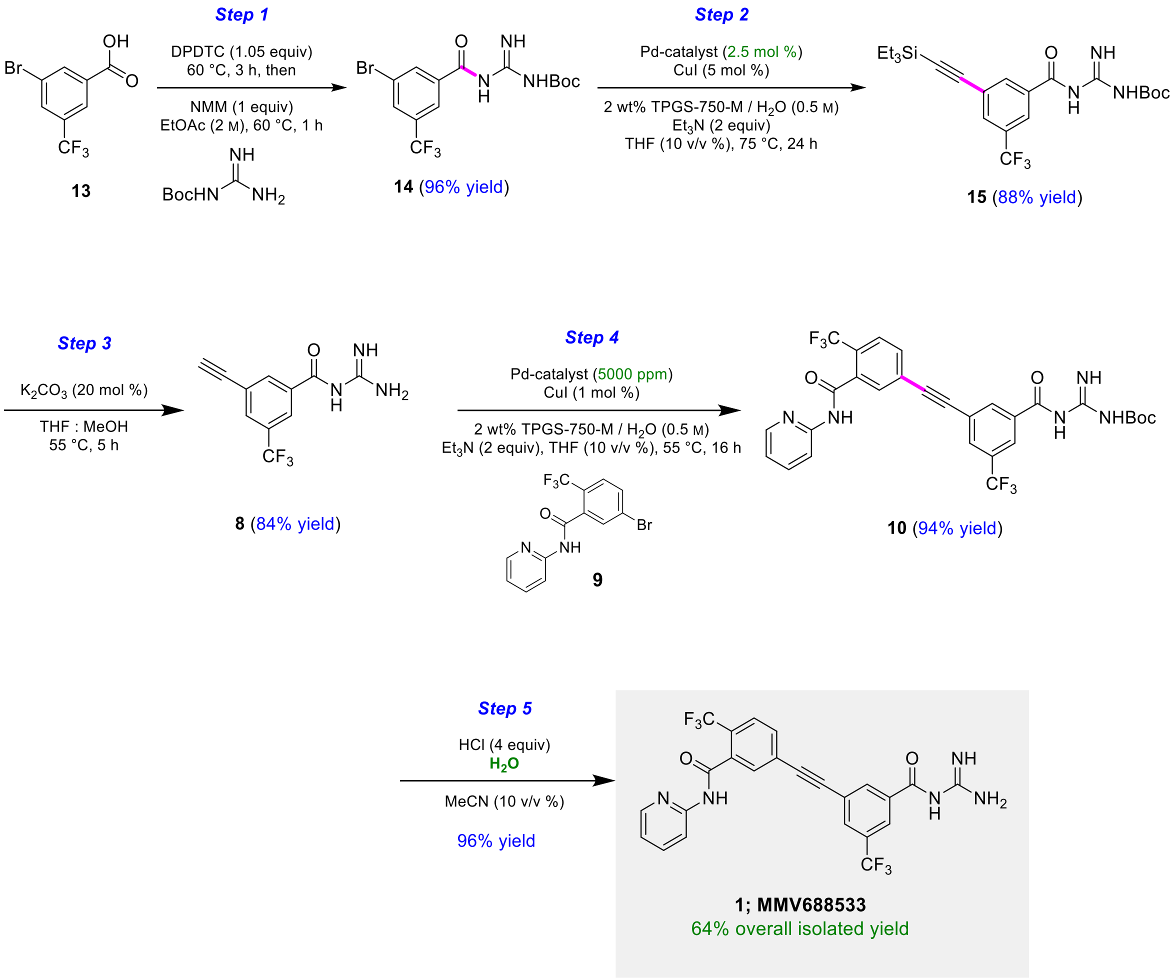

Scheme S2: UCSB synthesis of MMV688533 via Route 2.

3. General procedures

3.1 Optimization for amide bond formation

Table S1. Screening of activating agents for the amide coupling to afford **14**.

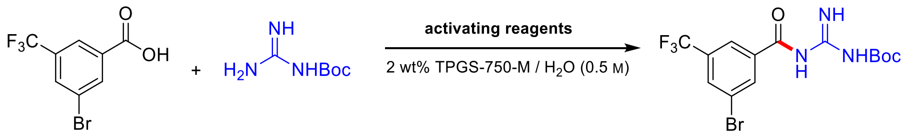

13 **14**

entry	activating reagents	time (h)	temp (°C)	base	yield (%)[a]
1	DPDTC (1.05 equiv)	8	60	NMM (1.05 equiv)	73
2[b]	DPDTC (1.05 equiv)	4	60	NMM (1.05 equiv)	85
3	COMU (1.05 equiv)	8	30	2,6-lutidine (3.1 equiv)	messy
4[c]	EDCI•HCl (1.1 equiv) HOBt (1.2 equiv)	18	45	–	45
5	DCC (1.5 equiv)	18	45	–	19
6	cyanuric Chloride (1 equiv)	8	rt	NMM (1 equiv)	trace
7[d]	thionyl Chloride (7 equiv)	8	50	K_2CO_3 (8 equiv)	trace
8[c]	EDCI•HCl (1.1 equiv)/ HOBt (1.2 equiv)	16	rt	–	61
9[e]	T3P (1.2 equiv)	8	rt	DIPEA (3 equiv)	27
**10[f]**	**DPDTC (1.05 equiv)**	**8**	**60**	**NMM (1.05** equiv**)**	**96**
11[e]	DPDTC (1.05 equiv)	8	60	NMM (1.05 equiv)	94

[a] Isolated yield; [b] 10 mol % DMAP was used; [c] reaction was run in DMF; [d] The acid chloride of **13** was pre-formed in neat thionyl chloride before addition to an aqueous solution of guanidine and K_2CO_3; [e] Reaction run in EtOAc (2 M); [f] Reaction was run in the absence of solvent (neat).

Activating agent abbreviations:

- DPDTC: di-2-pyridyldithiocarbonate
- EDCI: 1-ethyl-3-(3-dimethylaminopropyl)carbodiimide
- COMU: (1-cyano-2-ethoxy-2-oxoethylidenaminooxy)dimethylamino-morpholino-carbenium hexafluorophosphate
- DCC: *N,N'*-dicyclohexylcarbodiimide
- HOBt: 1-hydroxybenzotriazole
- T3P: propanephosphonic acid anhydride

a. General procedure for amide couplings using DPDTC (Table S1; entries 1, 2, 10 and 11):

1. *Procedure for amide bond formation under aqueous conditions* (entries 1 and 2): To a 2-dram vial containing a PTFE-coated magnetic stir bar was added 3-bromo-5-(trifluoromethyl) benzoic acid **13** (1 equiv, 0.5 mmol) and DPDTC (1.05 equiv, 0.52 mmol, 130.2 mg). The contents were stirred at 60 °C until full consumption of the acid, as determined by TLC (~4 – 6 h). Upon complete consumption of the acid, *N*-Boc-guanidine (1.00 equiv, 79.6 mg) was added followed by addition of *N*-methylmorpholine (1.00 equiv, 54 µL) and an aqueous solution of 2 wt % TPGS-750-M (0.5 M, 1 mL), and the contents were stirred at 60 °C until complete consumption of the thioester was observed. The crude reaction mixture was extracted with EtOAc (1.5 mL) and concentrated *in vacuo*, after which it was subjected to flash chromatography (10 – 30% EtOAc/hexanes as eluent) to afford product **14** as a white solid.

2. *Procedure for amide bond formation under neat conditions* (entry 10): To a 2-dram vial containing a PTFE-coated magnetic stir bar was added 3-bromo-5-(trifluoromethyl) benzoic acid **13** (1 equiv, 1 mmol) and DPDTC (1.05 equiv, 1.05 mmol, 260.4 mg). The contents were stirred at 60 °C until full consumption of the acid, as determined by TLC (~4 – 6 h). Subsequently, the *N*-Boc-guanidine was added, in a 1-pot fashion to the vial followed by the addition of *N*-methylmorpholine (NMM, 1.00 equiv, 108 µL) and stirred until complete consumption of the thioester. The crude reaction mixture was dissolved in EtOAc and concentrated *in vacuo*, after which it was subjected to flash chromatography (10 – 30% EtOAc/hexanes as the eluent) to afford product **14** as a white solid.

3. *Procedure for amide bond formation using EtOAc* (2 M; entry 11): To a 2-dram vial containing a PTFE-coated magnetic stir bar was added 3-bromo-5-(trifluoromethyl) benzoic acid **13** (1 equiv, 1 mmol) and DPDTC (1.05 equiv, 1.05 mmol, 260 mg). The contents were stirred at 60 °C until full consumption of the acid, as determined by TLC (~4–6 h). Upon complete consumption of the acid, the *N*-Boc-guanidine (1.00 equiv, 159.2 mg) was added followed by addition of EtOAc (2 M, 500 µL) and *N*-methylmorpholine (1.00 equiv, 108 µL), and the contents were stirred at 60 °C until complete consumption of the thioester was observed. The crude reaction mixture was further diluted with EtOAc (1.5 mL) and concentrated *in vacuo*,

whereupon it was subjected to flash chromatography (10 – 30% EtOAc/hexanes as the eluent) to afford product **14** as a white solid.

b. General procedure for amide couplings using other coupling reagents:

To a 1-dram vial with a PTFE-coated magnetic stir-bar was added 3-bromo-5-(trifluoromethyl) benzoic acid **13** (1.00 equiv, 0.500 mmol), *N*-Boc-guanidine (1.2 equiv, 95.5 mg) and base. 2 wt % TPGS-750-M in water (0.5 M, 1.00 mL) was added followed by addition of the activating agent. The reaction mixture was stirred at the indicated temperature for the indicated amount of time. Upon, completion, the crude mixture was extracted with EtOAc (2.5 mL) and subjected to flash chromatography (10 – 30 % EtOAc/hexanes as eluent) to afford product **14**, as a white solid.

3.2 Optimization of the first Sonogashira coupling

Table S2. Catalyst screening for the first Sonogashira coupling to afford **15**.

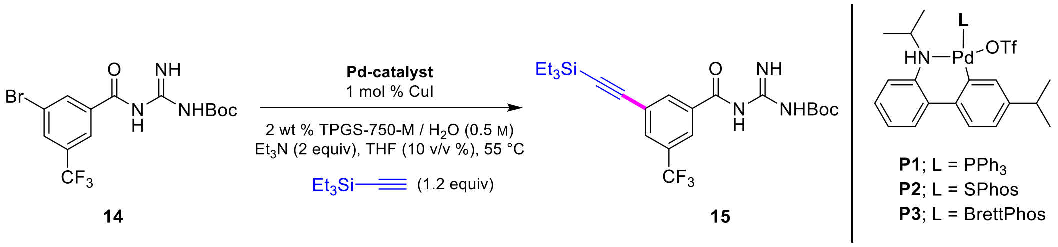

entry[a]	Pd catalyst / ligand	yield (%)[b]
1	$[Pd(cinnamyl)Cl]_2$ (1 mol %) cBRIDP (2 mol %)	2
2	$PdCl_2$(dppf) • DCM (1 mol %)	68
3	Pd(dtbpf)Cl_2 (1 mol %)	20
4	$PdCl_2(PPh_3)_2$ (1 mol %)	41
5	Xantphos Pd G4 (1 mol %)	61
6	SPhos Pd G3 (1 mol %)	trace
7	FeNPs (5 mol %), $Pd(OAc)_2$ (1 mol %), XPhos (3 mol %)	15
8	$Pd(PPh_3)_4$ (1 mol %)	60
**9[c]**	**$Pd(PPh_3)_4$ (2.5 mol %)**	**91(88)**
10[d]	$PdCl_2$(dppf) • DCM (2 mol %)	84
11[d]	BrettPhos Pd G3 (2 mol %)	12
12[d]	*N*-XantPhos Pd G3 (2 mol %)	74

13	**P1** (1 mol %)	48
14	**P2** (1 mol %)	0
15	**P3** (1 mol %)	50

[a] Reactions were carried out on a 0.25 mmol scale; [b] NMR yields using 1,3,5-trimethoxybenzene as an internal standard; [c] 5 mol % CuI was used; [d] 4 mol % CuI was used.

General procedure for Sonogashira coupling to arrive at 15:

To a 1-dram vial containing a PTFE-coated magnetic stir-bar was added the aryl bromide **14** (0.25 mmol, 102.5 mg), followed by addition of the Pd catalyst as indicated ($Pd(PPh_3)_4$ was added inside the glovebox). The vial was evacuated and back-filled with argon three times and was taken inside the glovebox, wherein CuI was added. A solution of 2 wt % TPGS-750-M (0.45 mL), THF (0.05 mL) and Et_3N (2 equiv, 104.5 µL) were added via a syringe under a positive flow of argon, followed by the addition of triethylsilylacetylene (1.2 equiv, 0.3 mmol, 48 µL) and the reaction mixture was allowed to stir vigorously at 55 ^oC for 24 h. The reaction mixture was extracted with EtOAc (3 x 0.5 mL). The combined organic layers were concentrated *in vacuo*. Subsequently, 0.5 mL $CDCl_3$ was added followed by the addition of 1,3,5-trimethoxybenzene (5 – 7 mg) as internal standard, and the samples were analyzed by ^1H NMR (20 sec relaxation delay; the aromatic peaks are well separated).

Fe/ppm Pd nanoparticle-mediated Sonogashira coupling (entry 7):

To a 1-dram vial containing a PTFE-coated magnetic stir bar were added pure $FeCl_3$ (2.1 mg, 5 mol %) and XPhos (3.56 mg, 3 mol %) under anhydrous conditions. The reaction vial was closed with a rubber septum and the mixture was evacuated and backfilled with argon. Dry THF (0.5 mL) was added to the vial and 100 µL solution of $Pd(OAc)_2$ (1 mol %) from a stock solution was then added (the stock solution was prepared by dissolving 5.6 mg $Pd(OAc)_2$ in 1 mL dry THF). The mixture was stirred for 15 min at rt, after which dissolution and complexation of iron trichloride was clearly visualized by a color change to dark brown. While maintaining an inert atmosphere, THF was evaporated under reduced pressure at rt. MeMgBr in THF (0.12 mL, 10 mol %, 0.2 M) was added to the reaction mixture, which was then stirred at rt for 1 min. A freshly degassed aqueous solution of 2 wt % TPGS-750-M/H_2O (1 mL) was added to the vial and the mixture was stirred for 1 min. The aryl bromide **14** (0.25 mmol, 102.5 mg), triethylsilylacetylene (1.2 equiv, 0.3 mmol, 48 µL), and Et_3N (0.5 mmol, 2 equiv) were sequentially added to the reaction vial. The

vial was sealed with a rubber septum under argon and then stirred at 45 °C for 24 h. After 24 h, the reaction mixture was cooled to rt. EtOAc (1.0 mL) was added to the reaction mixture and then stirred *gently* for 5 min at rt. Stirring was then stopped and the organic layer was decanted using a pipette. Subsequently, 0.5 mL of $CDCl_3$ was added followed by the addition of 1,3,5-trimethoxybenzene (5 – 7 mg) as the internal standard and the samples were analyzed by ^{1}H NMR.

3.3 Optimization for synthesis of Intermediate 16

Table S3. Optimization of reaction conditions for Sonogashira coupling.

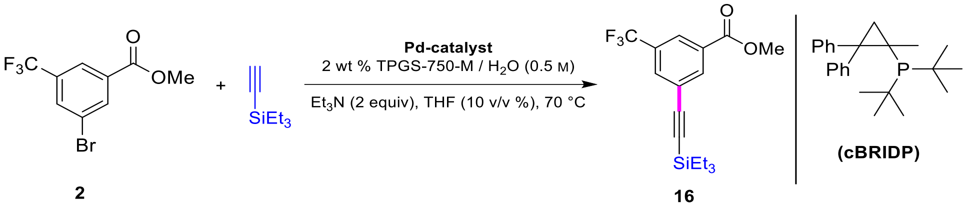

entry[a]	Pd catalyst	yield[b]
1	$[Pd(cinnamyl)Cl]_2$ (0.25 mol %)<br>cBRIDP (1 mol %)	95
2	$Pd(PPh_3)_4$ (0.5 mol%)/CuI (1 mol %)	94
**3**	**$[Pd(cinnamyl)Cl]_2$ (0.125 mol %)<br>cBRIDP (0.50 mol %)**	**96**
4	$[Pd(cinnamyl)Cl]_2$ (0.05 mol %)<br>cBRIDP (0.2 mol %)	84

[a] Reactions were carried out on a 1 mmol scale; [b] Isolated yield.

Preparation of stock solution of catalyst: In a 1-dram vial containing a PTFE coated magnetic stir bar, cBRIDP (17.6 mg, 0.05 mmol) and $[(cinnamyl)PdCl]_2$ (6.5 mg, 0.0025 mmol) were added in a glove box. The reaction vial was sealed with a rubber septum and degassed, after which anhydrous THF (2 mL) was added via syringe. The mixture was stirred for 5 min at rt. A yellow stock solution was obtained for subsequent Sonogashira reactions (*Note: always use fresh stock solution because it is unstable at rt, as indicated by the color change from yellow to orange to black within one week*).

Procedure for Sonogashira coupling (entry 3):

To a 2-dram vial containing a PTFE coated magnetic stir-bar was added aryl bromide **2** (1 mmol, 1 equiv, 283 mg) and the vial was evacuated and backfilled with argon three times. An aqueous solution of 2 wt % TPGS-750-M (0.5 M, 1.8 mL) was added followed by the addition of the catalyst stock solution (200 µL for 2500 ppm of Pd) and Et_3N (2 equiv, 2 mmol, 279 µL). Lastly, triethylsilyl-acetylene (1.2 equiv, 1.2 mmol, 192 µL) was added under a positive flow of argon and the reaction was vigorously stirred at 70 °C for 24 h. After complete consumption of starting material, as monitored by TLC, the reaction mixture was extracted with EtOAc (1.0 mL x 3). The combined organic layer was dried over anhydrous Na_2SO_4 and the solvent was removed *in vacuo*. The crude product was purified by flash chromatography over silica gel (1% EtOAc/hexanes) to afford **16** as light-yellow thick liquid (327.2 mg, 96% yield).

Gram-scale synthesis of intermediate 16

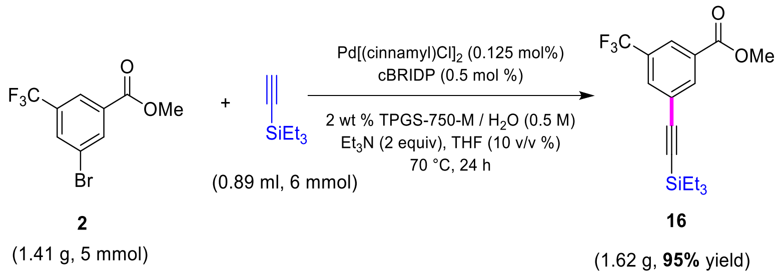

Scheme S3: Gram-scale synthesis of intermediate **16**

Procedure For Gram-Scale Reactions

To a 25 mL cylindrical Schlenk flask equipped with a PTFE-coated magnetic stir bar was added methyl 3-bromo-5-(trifluoromethyl) benzoate (**2**, 1 equiv, 5 mmol, 1.41 g) and the flask was then transferred to a glove box where Pd[(cinnamyl)Cl]$_2$ (0.125 mol %, 3.23 mg, 0.006 mmol) and cBRIDP (0.5 mol %, 0.012 mmol, 8.8 mg) were added under argon. The side-arm of the tube was sealed with a rubber septum and the flask was removed from the glove box, after which a solution of 2 wt % TPGS-750-M/H_2O (9 mL), THF (1 mL), and Et_3N (2 equiv, 1.39 mL, 10 mmol) were added under a flow of argon and the mixture allowed to stir for 10 min at rt. The triethylsilyl-acetylene (1.2 equiv, 6 mmol, 0.89 mL) was added slowly via syringe and the flask was sealed and allowed to stir at 70 °C for 24 h. Upon completion, the reaction mixture was extracted with EtOAc

(3 x 5.0 mL). The combined organic layer was dried over anhydrous Na_2SO_4 and the solvent was removed *in vacuo*. The crude product was purified by flash chromatography over silica gel (1% EtOAc/hexanes) to afford product **16** as light-yellow thick liquid (1.62 g, 95% yield).

3.4 Synthesis of Intermediate 8

1. Deprotection of the triethylsilyl group to afford **4**.

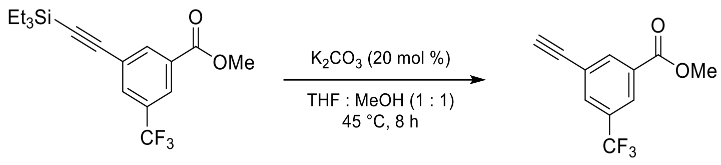

16 4

Scheme S4: Deprotection of the triethylsilyl group

Procedure: To a 6-dram vial containing a PTFE coated magnetic stir bar was added compound **16** (1.9 mmol, 650 mg) followed by the addition of K_2CO_3 (20 mol %, 52 mg) and a 1:1 mixture of THF and MeOH (2 mL each) to adjust the global concentration to 0.4 M. The reaction mixture was stirred at 50 ^oC for 8 h, whereupon full removal of the TES group was observed by TLC (2% EtOAc/hexanes). Upon completion, the reaction mixture was concentrated *in vacuo* and subjected to flash chromatography using silica gel (1 – 2% EtOAc/hexanes as eluent) to afford product **4** as a light-yellow color oil (363.4 mg, 84% yield).

2. Ester hydrolysis to afford acid **5**

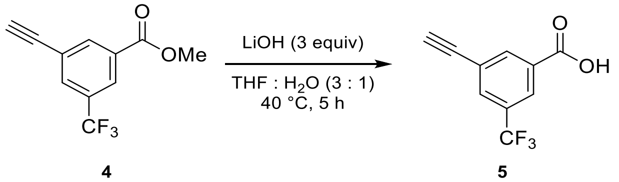

4 5

Scheme S5: Ester hydrolysis of **4**

Procedure: In a 2-dram vial containing a PTFE coated magnetic stir bar, methyl ester **4** (1.27 mmol, 290 mg) was added followed by the addition of LiOH (3 equiv, 91.6 mg). A mixture of THF (6 mL) and H_2O (2 mL) were added via syringe and the reaction mixture was stirred at 50 ^oC

for 5 h. Upon completion, the crude mixture was concentrated *in vacuo* to remove THF and the residue was acidified to pH 2 – 3 using a 1 M aqueous HCl. The light brown precipitate of product was collected via centrifugation and washed with water three times by repeated resuspension in DI water and centrifugation, then dried under vacuum at 50 °C for 16 h to afford product **5** as a light brown - buff colored solid (250.3 mg, 92% yield).

3. Amide coupling to afford **8:**

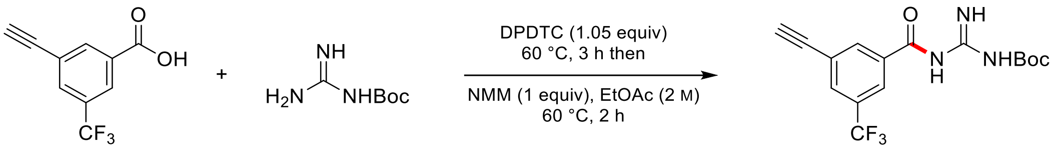

5 **8** (87% yield)

Scheme S6: Amide bond formation to afford **8**

Procedure: To a 1-dram vial containing a PTFE-coated magnetic stir bar was added the carboxylic acid **5** (107 mg, 1 equiv, 0.5 mmol) and DPDTC (129 mg, 1.05 equiv, 0.52 mmol). The reaction mixture was stirred at 60 °C for 3 h. Upon complete consumption of the acid, the reaction mixture was cooled to rt and *N*-Boc-guanidine (1.05 equiv, 87.6 mg) was added followed by addition of EtOAc (2 M, 250 µL) and *N*-methylmorpholine (NMM, 47 µL, 1.0 equiv, 0.5 mmol). The reaction was stirred at 60 °C for 2 h, until complete consumption of the thioester. Upon completion, the reaction mixture was first diluted with 1.5 mL of EtOAc, then washed with 1 M NaOH (2 × 0.5 mL) to remove 2-mercaptopyridine. The organic layer was concentrated to afford crude product. The crude product was purified by silica gel flash chromatography (5 – 20% EtOAc/hexanes as eluent) to give the pure product **8** as a white solid (154 mg, 87% yield).

4. 2-Step, 1-pot deprotection of the triethylsilyl group/ester hydrolysis to afford **5:**

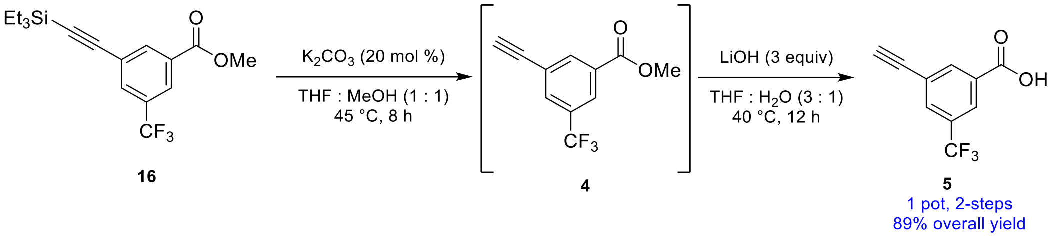

Scheme S7. 2-Step, 1-pot synthesis of **5**

Procedure: To a 2-dram vial containing a PTFE coated magnetic stir bar was added compound **16** (325 mg, 0.95 mmol) followed by the addition of K_2CO_3 (20 mol %, 26.2 mg) and a 1:1 mixture of THF and MeOH (1 mL each). The reaction mixture was stirred at 45 ^oC for 8 h, whereupon full deprotection of the TES group was observed by TLC. The mixture was cooled to rt and LiOH (3 equiv, 68 mg) and H_2O (1 mL) were added and the reaction mixture was stirred at 50 ^oC for 12 h. Upon completion of ester hydrolysis, the crude mixture was concentrated *in vacuo* to remove THF and MeOH and the resulting residue was acidified to pH 3 – 4 using 1 M aqueous HCl. The light brown precipitate of product was collected via centrifugation and washed with water three times by repeated resuspension in DI water and centrifugation, then dried under vacuum at 50 °C for overnight to afford the product **5** light brown – buff colored solid (190.1 mg, 89% yield).

5. 4-Step, 1-pot sequence to afford carboxylic acid **8**:

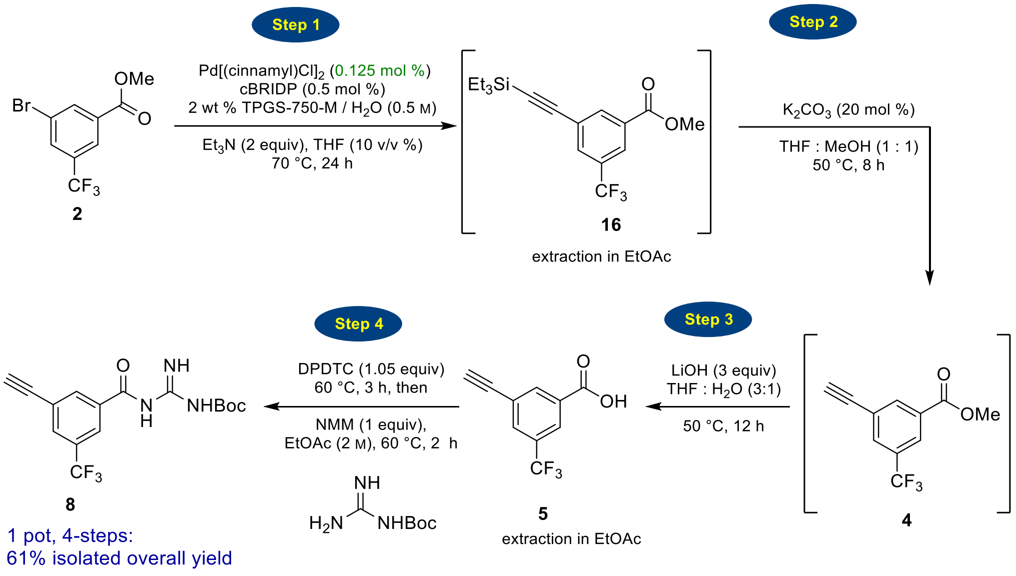

Scheme S8. 4-Step, 1-pot synthesis of **8**

Procedure for 4-step, 1-pot synthesis of 8

Step 1 – Sonogashira coupling to afford **16**: To a 2-dram vial equipped with a PTFE-coated magnetic stir bar was added methyl 3-bromo-5-(trifluoromethyl) benzoate **2** (1 mmol, 1 equiv, 283 mg) and the vial was evacuated and backfilled with argon three times. An aqueous solution of 2 wt % TPGS-750-M (0.5 M, 1.8 mL) was added followed by the addition of the catalyst stock solution (200 µL for 2500 ppm of Pd, (0.125 mol % of dimer), see ESI, section 3.3) and Et_3N (2 equiv, 2 mmol, 279 µL). Lastly, triethylsilylacetylene (1.2 equiv, 1.2 mmol, 192 µL) was added under a positive flow of argon and the reaction was vigorously stirred at 70 °C for 24 h. Upon completion (by TLC), the reaction mixture was extracted with EtOAc (3.0 mL) and brine (1 mL), and the layers were allowed to separate. The aqueous layer was removed using a syringe and the resulting organic layer was concentrated, in the same vial to afford crude **16**. This material was used in the next step without further purification.

Step 2 – Triethylsilyl deprotection to afford terminal alkyne **4**: The crude material from the previous step was subjected to K_2CO_3 (20 mol %, 27.6 mg) and a 1:1 mixture of THF and MeOH

(1 mL each). The reaction was stirred at 50 °C for 8 h, until complete deprotection of the TES group was observed (by TLC or GC-MS). the reaction mixture was cooled to rt and was used in the next step without further purification.

Step 3 – Ester hydrolysis to afford carboxylic acid **5**: The material from the previous step was subjected to LiOH (3 equiv, 3 mmol, 72 mg), followed by addition of water (1 mL). The reaction vial was capped and stirred for 5 h at 50 °C. Upon completion, the reaction mixture was concentrated *in vacuo* to remove most of the THF and MeOH, then acidified to pH 2 using a 1 M aqueous solution of HCl. The aqueous phase was extracted with EtOAc (3 mL), brine (1 mL), and the layers were allowed to separate. The aqueous layer was removed using a syringe and the resulting organic layer was concentrated *in vacuo* to afford the crude carboxylic acid **5**. This material was used in the subsequent step without further purification.

(Step 4) Amide bond formation to afford **8**: To the crude material from the previous step was added DPDTC (1.05 equiv, 1.05 mmol, 260.4 mg). The contents were stirred at 60 °C until full consumption of the acid was observed, as determined by TLC (~3 h). Upon complete consumption of the acid, *N*-Boc-guanidine (1.05 equiv, 175.1 mg) was added followed by addition of EtOAc (2 M, 0.5 mL) and *N*-methylmorpholine (1.05 equiv, 115 µL), and the contents were stirred at 60 °C until complete consumption of the thioester was observed (~2 h). The crude reaction mixture was further diluted with EtOAc (1.5 mL) and concentrated *in vacuo*, whereupon it was subjected to flash chromatography (10 – 20% EtOAc/hexanes as eluent) to afford product **8** as a white solid (216 mg, 61% yield).

3.4 Synthesis of 9 from methyl 5-bromo-2-(trifluoromethyl)benzoate

Table S3. Optimization of amide bond formation from ester **11**

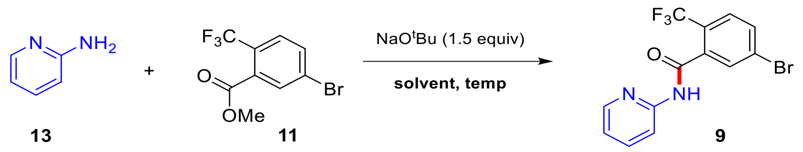

entry	solvent	temp (°C)	Time (h)	yield (%)[b]
1	2 wt % TPGS-750-M / H_2O	rt	16	–
2	2 wt % TPGS-750-M / H_2O	80	16	15
3	neat	rt	2	59
4	neat	rt	6	66
**5**	**neat**	**60**	**4**	**96**
6[c]	2-MeTHF	55	14	95
7[c,d]	2-MeTHF	55	14	98
**8[c,e]**	**2-MeTHF**	**55**	**14**	**>99**

[a] Reaction condition: **13** (0.25 mmol), **11** (0.37 mmol), solvent (0.5 M); [b] Isolated yield; [c] solvent (2 M) was used; [d] **11** (0.3 mmol) was used; [e] **11** (0.27 mmol) was used.

General procedure for direct amidation to afford 9:

2-Aminopyridine **13** (1 equiv, 0.25 mmol, 23.5 mg) and methyl 5-bromo-2-(trifluoromethyl) benzoate (**11**, 1.5 equiv, 0.37 mmol, 106.1 mg) were placed in a 1-dram vial with a PTFE-coated magnetic stir bar which was covered with a rubber septum. The vial was taken into the glovebox, in which NaO*t*Bu (1.5 equiv, 36.0 mg) was added. The indicated solvent (0.5 M) was added via syringe and the reaction mixture was allowed to stir at the indicated temperature for the indicated amount of time. Upon completion of the reaction, as monitored by TLC, the crude mixture was diluted (in the case of neat reactions) or extracted with EtOAc (3 x 0.5 mL) in the case of reactions in aqueous 2 wt % TPGS-750-M as the medium. Reactions run with 2-MeTHF were directly concentrated *in vacuo* and subjected to flash chromatography with silica gel (5–20% EtOAc/hexanes as eluent) to afford **9** as a white solid.

Gram-scale reaction to afford 9:

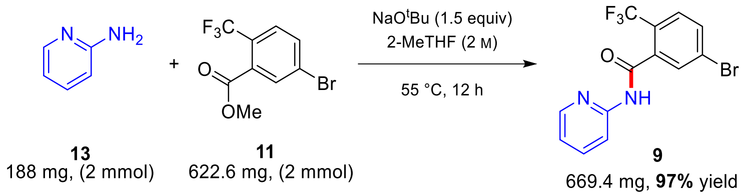

Scheme S9. Gram-scale reaction for the synthesis of **9**

Procedure: 2-Aminoppyridine **13** (1 equiv, 2 mmol, 188 mg) and methyl 5-bromo-2-(trifluoromethyl) benzoate (**11**, 1.1 equiv, 2.2 mmol, 622.6 mg) were placed in a 1-dram vial with a PTFE-coated magnetic stir bar and then covered with a rubber septum. The vial was taken into the glovebox and NaO^{t}Bu (1.5 equiv, 288 mg) was added and the flask was sealed with a rubber septum, and then removed from the glove box. The mixture was dissolved in 2-MeTHF (2 M, 1 ml) and stirred at 50 °C for 12 h. Upon completion (as monitored by TLC), water (1 mL) was added dropwise to quench the reaction and the resulting mixture was stirred for another 10 min and extracted with EtOAc (3 x 1 mL). The combined organic layers were concentrated *in vacuo* and subjected to flash chromatography (5 – 20% EtOAc/hexanes as eluent) to afford **9** (669.4 mg, 97% yield as a white color solid.

3.5 Synthesis of 9 from carboxylic acid 11

Table S4. Optimization of amide bond formation from carboxylic acid **11**

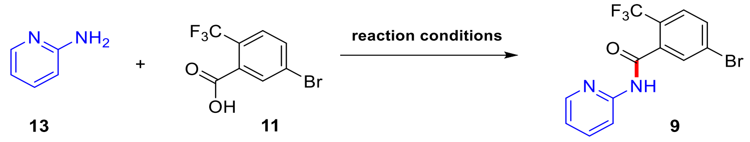

Entry[a]	reaction condition	yield of 9 (%)[b]
1	DPDTC (1.1 equiv), DMAP (10 mol %), 60 °C, 3 h, then EtOAc (2 M), NMM (1.1 equiv), 60 °C, 12 h	15
**2**	**T3P (1.2 equiv), EtOAc (2 M), rt, 12 h**	**47**
3	HATU (1.1 equiv), DIPEA (3 equiv), DMF, rt, 16 h	18
4	HATU (1.1 equiv), DIPEA (3 equiv), DMF, rt, 4 h	trace
5	EDCI (1.2 equiv), HOBt (1.2 equiv), DMF, rt, 8 h	12

[a] Reaction conditions: 0.25 mmol **13**, 0.275 mmol **11**; [b] Isolated yield.

Procedure (entry 2): To a 1-dram vial equipped with a PTFE-coated magnetic stir bar, were added carboxylic acid **11** (1.0 equiv, 0.25 mmol, 23.5 mg), 2-aminopyridine **13** (1.1 equiv, 0.275 mmol, 26 mg), and DIPEA (3 equiv, 0.75 mmol, 130.6 µL) followed by the addition of anhydrous EtOAc (0.125 mL). The resulting solution was cooled to -10 °C using an acetone-ice bath. The mixture was stirred for 5 min at this temperature after which a 50 w/w % solution of propanephosphonic acid anhydride (T3P) in EtOAc (1.2 equiv, 0.3 mmol, 179 µL) was added dropwise with stirring and the resulting reaction mixture was stirred for 30 min at this temperature, at which time it was allowed to warm to rt and further stirred for 3 h. On completion of the reaction (as monitored by TLC), the mixture was diluted with EtOAc (0.5 mL) and quenched by addition of water (0.5 mL) and the aqueous layer was extracted 3 times with EtOAc (0.5 mL). The combined organic layers were concentrated *in vacuo* to give crude material which was then purified by flash chromatography (10% EtOAc/hexanes) to give pure product **9** (40.3 mg, 47%) as a white solid.

Procedure (entries 3–5): In a 1-dram vial, carboxylic acid **11** (1.0 equiv, 0.25 mmol, 23.5 mg) , amine **13** (1.1 equiv, 0.275 mmol, 26 mg), and base were added followed by the addition of DMF (0.5 mL). The vial was capped and stirred at rt for ~5 min, after which the coupling reagent (1.2 equiv) was added. The reaction was stirred at rt for the indicated amount of time. The reaction mixture was first concentrated to remove most of the DMF and then diluted with H_2O, followed by extraction with EtOAc (3 x 1 mL). The organic was concentrated *in vacuo* to give crude material which was then purified by flash chromatography (10% EtOAc/hexanes) to give pure product **9**.

Procedure (entry 1): To a 1-dram vial equipped with a PTFE-coated magnetic stir bar, were added carboxylic acid **11** (1.0 equiv, 0.25 mmol, 23.5 mg) and DPDTC (1.1 equiv, 275 mmol, 68.2 mg). The reaction mixture was allowed to stir at 60 ^{o}C for 3 h until complete consumption of the carboxylic acid was observed. After 3 h, the reaction mixture was cooled to rt and amine **13** (1.1 equiv, 26 mg) was added followed by addition of EtOAc (2 M, 125 µL) and *N*-methylmorpholine (NMM, 30 µL, 1.1 equiv, 0.275 mmol). The reaction was stirred at 60 °C for 2 h, until complete consumption of the thioester. Upon completion, the reaction mixture was first diluted with 1.5 mL of EtOAc, then washed with 1 M NaOH (2 × 0.5 mL) to remove 2-mercaptopyridine. The organic layer was concentrated to afford crude product. The crude product was purified by silica gel flash chromatography (10% EtOAc/hexanes as eluent) to give the pure product **9**.

3.6 Synthesis of 10

Table S5. Optimization of reaction conditions for Sonogashira coupling to **10**.

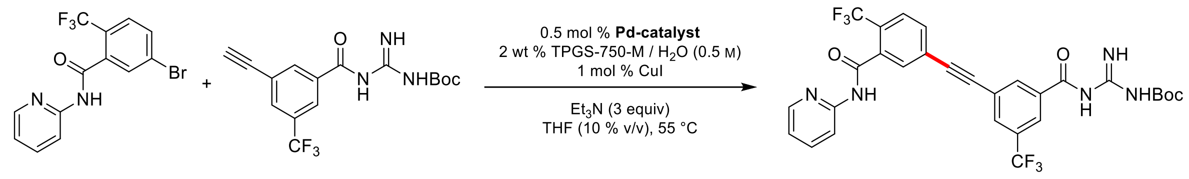

9 8 10

entry	Pd catalyst	time	yield (%)[a]
1[b]	Pd[(cinnamyl)Cl]$_2$ (0.25 mol %)	24	19
	cBRIDP (1 mol %)		
2	Pd(PPh$_3$)$_2$Cl$_2$	24	47
3	Pd(dppf)Cl$_2$•DCM	24	29
4	Pd(dtbpf)Cl$_2$	24	51
5	XantPhosPd G4	24	93
6	Pd(PPh$_3$)$_4$	24	96
**7[c]**	**Pd(PPh$_3$)$_4$**	**16**	**94**
8[d]	Pd(PPh$_3$)$_4$	24	68
9[e]	Pd(PPh$_3$)$_4$	16	87
10	PdCl$_2$(CH$_3$CN)$_2$	24	34
11[f]	Pd(PPh$_3$)Cl$_2$	24	30

Reaction conditions: **9** (0.25 mmol), **8** (0.30 mmol), Et$_3$N (3 equiv), solvent (0.5 M); [a] Isolated yield; [b] The reaction was run in the absance of CuI; [c] Reaction performed on 0.5 mmol scale; [d] Pd(PPh$_3$)$_4$ (2500 ppm; 0.25 mol %) was used; [e] Reaction run in absence of co-solvent; [f] Reaction was performed in EtOAc (0.5 M).

General procedure for Sonogashira coupling to afford 10:

To a 1-dram vial with a PTFE coated magnetic stir bar were added compounds **9** (1.0 equiv, 0.25 mmol, 86.3 mg) and **8** (1.2 equiv, 0.3 mmol, 106.5 mg) and the vial was taken into a glovebox. The indicated Pd catalyst (0.5 mol %), along with CuI (1 mol %) were added. The flask was then sealed with a rubber septum and subsequently, 2 wt % TPGS-750-M aqueous solution (0.45 mL) was added followed by the addition of THF (10 v/v %, 0.05 mL) and Et$_3$N (3 equiv, 105 µL) and the reaction mixture was stirred at 70 ^{o}C for 16 h. Upon completion, the crude mixture was extracted with EtOAc (3 x 3 mL; due to moderate solubility of the product in EtOAc, more volume was needed for carrying out the extractions) and the combined organic layers were concentrated

in vacuo and subjected to flash chromatography using silica gel (30% EtOAc/hexanes as eluent) to afford **10** as an off white colored solid.

Procedure for Sonogashira coupling to afford 10 (entry 7):

To a 2-dram vial with a PTFE coated magnetic stir bar were added compounds **9** (0.5 mmol, 172.5 mg) and **8** (1.2 equiv, 0.6 mmol, 213 mg). The vial was taken into the glovebox, where $Pd(PPh_3)_4$ (0.5 mol %, 2.88 mg, 0.0025 mmol) and CuI (1 mol %, 1 mg, 0.005 mmol) were added and the flask was then sealed with a rubber septum, after which it was removed from the glove box. Subsequently, 2 wt % TPGS-750-M aqueous solution (0.9 mL) was added followed by the addition of THF (10 v/v %, 0.1 mL) and Et_3N (3 equiv, 210 μL) and the reaction mixture was stirred at 70 ^{o}C for 16 h. Upon completion, the crude mixture was extracted with EtOAc (3 x 5 mL) and the combined organic layers were concentrated *in vacuo* and subjected to flash chromatography using silica gel (30% EtOAc/hexanes as eluent) to afford **10** (290.5 mg, 94% yield) as an off-white solid.

3.7 *N*-Boc deprotection to afford MMV688533 (1)

Table S6. Optimization of *N*-Boc deprotection of **10** to afford **1.**

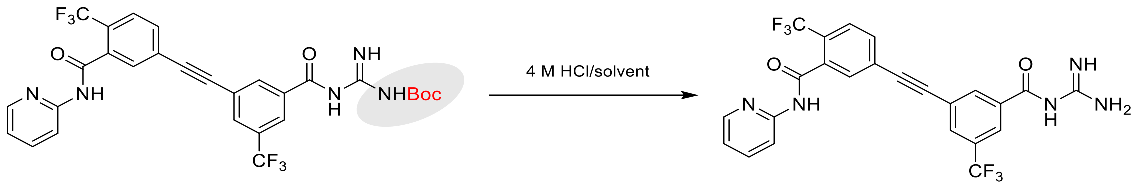

10

MMV688533 (1)

entry[a]	conditions	yield (%)[b]
1	TFA (20 equiv), neat, rt, 6 h	89
2	4 M HCl/dioxane (5 equiv), MeCN (0.5 M) rt, 6 h	trace[c]
3	1 M HCl/EtOAc (3 equiv), rt, 18 h	trace[c]
4	3 M HCl/MeOH (3 equiv), rt, 6 h	trace[c]
**5[d]**	**4 M HCl (5 equiv), H_2O, 10 % v/v MeCN**	**96**

[a] Reaction conditions: **10** (0.06 mmol), solvent (0.5 M); [b] Isolated yield; [c] Unreacted starting material (**10**) was observed; [d] Reaction performed on 0.125 mmol scale.

General procedure for optimizing *N*-Boc deprotection (Table S6; entries 1 – 4).

To a 1-dram vial with a PTFE coated magnetic stir bar was added the *N*-Boc-protected compound **10** (37.1 mg, 0.06 mmol) followed by the addition of the indicated solvent and hydrochloric acid (4 M in solvent, 5 equiv). Stirring was initiated and the reaction was allowed to stir at rt for the indicated time whereupon the product precipitated as a white solid and solvent was removed *in vacuo*. The resulting white solid was washed with Et_2O (3 × 1 mL) and collected via centrifugation and removal of the supernatant via syringe. The obtained white powder was analyzed by ^{1}H NMR.

Procedure for *N*-Boc deprotection of 10 to afford 1 (Table S6; entry 5).

Compound **10** (0.12 mmol, 77.4 mg, 1.00 equiv), water (0.25 mL), acetonitrile (10 v/v %, 25 µL) and hydrochloric acid (4 M in water, 3.00 equiv, 156 µL) were added to a 1-dram vial containing a magnetic stir bar. Stirring was initiated and the resulting solution was heated to 50 °C. The reaction mixture was stirred at 50 °C for 5 h. Upon complete consumption of *N*-Boc-protected starting material **10**, the reaction mixture was cooled to rt and the pH of the mixture was adjusted to approximately 11–12 by addition of sodium hydroxide (4 M in water, 0.3 mL) at rt. The slurry was then centrifuged and the solid residue was washed twice with water (2 x 1 mL) and then decanted. The isolated solids were dried under high vacuum at 55 °C for 12 h to afford compound **1** (62.1 mg, 96% yield) as a white solid.

4. PMI and E Factor calculations

Process mass intensity (PMI calculations)

$$PMI = \frac{Total\ Mass\ of\ Material\ Inputs\ (kg)}{Mass\ of\ Product\ (kg)}$$

Note: Calculations regarding column chromatographic purifications have not been considered.

A. Literature route: Sanofi[2]

Sanofi route to MMV688533

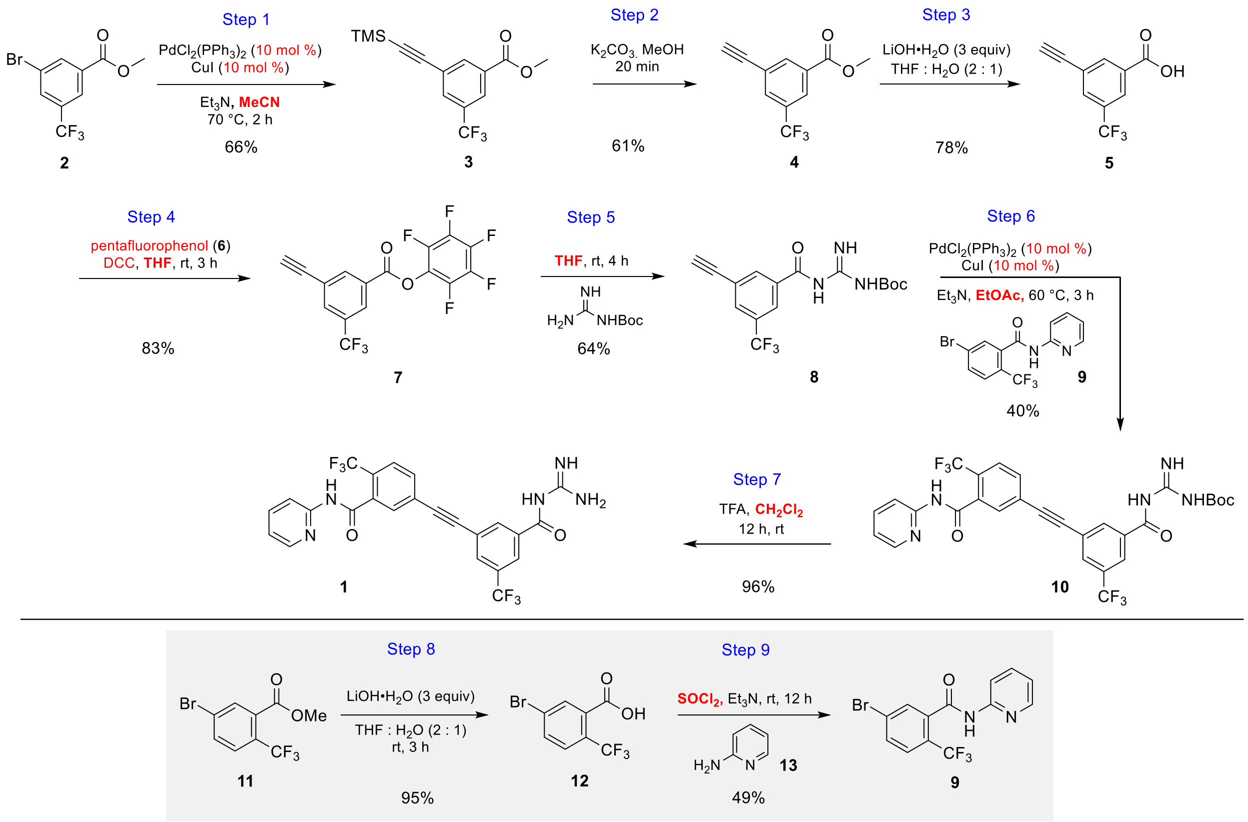

Scheme 1. Discovery route used by Sanofi *en route* to MMV688533 (**1**).

1. *Preparation of intermediate **3**:*

	**Material**	**Amount (g)**
**Limiting Reagent Input**	aryl bromide **2**	20
**Product Output**	compound **3**	14
**reagents**	TMS acetylene	17.40
	$Pd(PPh_3)_2Cl_2$	4.96
	CuI	1.34
	triethylamine	21.45
**solvents**	acetonitrile	78.6
**water**	N/A	
**totals**	all materials	143.75
	reagents	26.15
	solvents	78.6
	water	0

Step PMI : 10.3
Step PMI solvents : 5.6
Step PMI water : 0.0

2. *Preparation of intermediate **4**:*

	Material	Amount (g)
**Limiting Reagent Input**	compound **3**	14
**Product Output**	compound **4**	11
**Reagents**	K_2CO_3	0.58
**Solvents**	methanol	39.6
	ethyl acetate	90.2
**Aqueous**	brine	100
	water	100
**Totals**	all materials	344.4
	reagents	0.58
	solvents	129.8
	water	200

Note: Due to unavailability of data for volume of water used for washing, an amount equal to brine was considered for the calculations.

Step PMI : 31.3
Step PMI solvents : 11.8
Step PMI water : 18.2

3. *Preparation of intermediate **5**:*

	Material	Amount (g)
**Limiting Reagent Input**	compound **4**	11
**Product Output**	compound **5**	9.2
**Reagents**	LIthium hydroxide	6.0
**Solvents**	tetrahydrofuran	44.4
**Aqueous**	water	25
	aq. citric acid solution	50
**Totals**	all materials	136.4
	reagents	6.0
	solvents	44.4
	water	75

Note: Due to unavailability of data for volume of citric acid solution used for the acidification process, 50 mL was considered owing to the scale of the reaction.

Step PMI : 14.8
Step PMI solvents : 4.8
Step PMI water : 8.2

4. *Preparation of intermediate **7**:*

	Material	Amount (g)
**Limiting Reagent Input**	compound **5**	9.2
**Product Output**	compound **7**	13.6
**Reagents**	DCC	13.3
	Pentafluorophenol **(6)**	11.8
**Solvents**	tetrahydrofuran	44.4
**Aqueous**	N/A	0

**Totals**	all materials	69.5
	reagents	25.1
	solvents	44.4
	water	0

Step PMI : 5.8
Step PMI solvents : 3.3
Step PMI water : 0

5. *Preparation of intermediate **8**:*

	Material	Amount (g)
**Limiting Reagent Input**	compound **7**	13.6
**Product Output**	compound **8**	8.2
**Reagents**	*N*-Boc-guanidine	6.8
**Solvents**	tetrahydrofuran	44.4
**Aqueous**	N/A	0
**Totals**	all materials	14
	reagents	7
	solvents	44.4
	water	0

Note: Due to unavailability of data for volume of THF used, 50 mL was considered owing to the scale of the reaction.

Step PMI : 7.9
Step PMI solvents : 5.4
Step PMI water : 0

6. *Preparation of intermediate **10.***

	Material	Amount (g)
**Limiting Reagent Input**	compound **9**	25
**Product Output**	compound **10**	18
**Reagents**	compound **8**	28.3
	CuI	0.68
	triethylamine	22.0
	$Pd(PPh_3)_2Cl_2$	5.08
**Solvents**	ethyl acetate	225.5
**Aqueous**	N/A	0
**Totals**	all materials	307
	reagents	56
	solvents	226
	water	0

Step PMI : 17
Step PMI solvents : 12.5
Step PMI water : 0

7. *Preparation of intermediate **1**:*

	Material	Amount (g)
**Limiting Reagent Input**	compound **10**	18
**Product Output**	compound **1**	14.6
**Reagents**	trifluoroacetic acid	66.3
**Solvents**	dichloromethane	199.5
	diethyl ether	71.3
**Aqueous**	10% aq. $NaHCO_3$ solution	150
**Totals**	all materials	505
	reagents	66
	solvents	271
	water	150

Note: Due to unavailability of data for volume of diethyl ether used, 100 mL was assumed considering the scale of the reaction. 150 mL of $NaHCO_3$ solution was also used.

Step PMI : 34.6
Step PMI solvents : 18.5
Step PMI water : 10.3

8. *Calculation of cumulative PMI*

Step	***Step product***	***MW***	***Yield (%)***	***Usage factor (g)****	***Step PMI***	***Cumulative PMI contribution***	***Step PMI solvents***	***Cumulative PMI contribution solvents***	***Step PMI water***	***Cumulative PMI contribution water***
1	**3**	300.35	66	6.36	10.3	65.51	5.6	35.62	0	0
2	**4**	228.17	61	2.95	31.3	92.34	11.8	34.81	18.2	53.7
3	**5**	214.14	78	2.16	14.8	38.48	4.8	10.37	8.2	17.71
4	**7**	380.19	83	3.18	5.8	18.44	3.3	10.49	0	0
5	**8**	355.31	64	1.90	7.9	15.01	5.4	10.26	0	0
6	**10**	619.52	40	1.33	17	22.61	12.5	16.63	0	0
7	**1**	519.40	96	1.00	34.6	34.6	18.5	18.5	10.3	10.3

Usage factor = amount of compound required to synthesize 1 g of MMV688533 (1**).*

Cumulative PMI : 287
Cumulative PMI solvents : 136.7
Cumulative PMI water : 81.7

This work:

1. *Preparation of intermediate **16**:*

	Material	Amount (g)
**Limiting Reagent Input**	compound **2**	0.28
**Product Output**	compound **16**	0.32
**Reagents**	triethylsilyl)acetylene	0.16
	Et_3N	0.20
	[(cinnamyl)PdCl]$_2$	0.65
	cBRIDP	1.76

**Solvents**	terahydrofuran	1.77
	ethyl acetate	1.35
**Aqueous**	2 wt % TPGS-750-M/H_2O	1.8
**Totals**	all materials	7.97
	reagents	2.77
	solvents	3.12
	water	1.8

Step PMI : 24.9
Step PMI solvents : 9.8
Step PMI water : 5.6

2. *Preparation of intermediate **4**:*

	Material	Amount (g)
**Limiting Reagent Input**	compound **16**	0.65
**Product Output**	compound **4**	0.36
**Reagents**	K_2CO_3	0.52
**Solvents**	methanol	1.54
	tetrahydrofuran	1.77
**Totals**	all materials	4.48
	reagents	0.52
	solvents	3.31

Step PMI : 12.4
Step PMI solvents : 9.2
Step PMI water : 0

3. *Preparation of intermediate **5**:*

	Material	Amount (g)
**Limiting Reagent Input**	compound **4**	0.29
**Product Output**	compound **5**	0.25
**Reagents**	lithium hydroxide	0.91
**Solvents**	tetrahydrofuran	1.33
**Aqueous**	water	2.0
	1 M aq. HCl	0.5
**Totals**	all materials	5.03
	reagents	0.91
	solvents	1.33
	water	2.5

Step PMI : 20.1
Step PMI solvents : 5.3
Step PMI water : 10

4. *Preparation of intermediate **8**:*

	Material	Amount (g)
**Limiting Reagent Input**	compound **5**	0.107
**Product Output**	compound **8**	0.153
**Reagents**	DPDTC	0.129
	N-Boc guanidine	0.876
**Solvents**	ethyl acetate	1.58
**Aqueous**	1 M aq. NaOH	0.5
**Totals**	all materials	3.19
	reagents	1.05
	solvents	1.58
	water	0.5

Step PMI : 20.8
Step PMI solvents : 10.3
Step PMI water : 3.3

5. *Preparation of intermediate **10**:*

	Material	Amount (g)
**Limiting Reagent Input**	compound **9**	0.172
**Product Output**	compound **10**	0.290
**Reagents**	compound **8**	0.213
	CuI	0.001
	triethylamine	0.152
	$Pd(PPh_3)_4$	0.0028
**Solvents**	tetrahydrofuran	0.088
	ethyl acetate	2.7
**Aqueous**	2 wt % TPGS-750-M / H_2O	0.9

**Totals**	all materials	4.2288
	reagents	0.3688
	solvents	2.78
	water	0.9

Step PMI : 14.6
Step PMI solvents : 9.6
Step PMI water : 3.1

6. *Preparation of intermediate **1**:*

	Material	Amount (g)
**Limiting Reagent Input**	compound **10**	0.0774
**Product Output**	compound **1**	0.0621
**Reagents**	N/A	--
**Solvents**	acetonitrile	0.019
**Aqueous**	4 M aq. HCl	0.156
	H_2O	1.25
	4 M NaOH	0.3
**Totals**	all materials	1.8
	reagents	0.0
	solvents	0.019
	water	1.706

Step PMI : 29
Step PMI solvents : 0.3
Step PMI water : 27.5

7. Calculation of Cumulative PMI

Step	*Step product*	*MW*	*Yield (%)*	*Usage factor (g)**	*Step PMI*	*Cumulative PMI contribution*	*Step PMI solvents*	*Cumulative PMI contribution solvents*	*Step PMI water*	*Cumulative PMI contribution water*
1	**14**	353.33	96	1.1	24.9	28.84	9.8	10.78	5.6	6.16
2	**4**	228.17	84	0.62	12.4	7.79	9.2	5.7	0	0
3	**5**	214.14	92	0.54	20.1	10.9	5.3	2.86	10	5.4
4	**8**	355.31	86	0.77	20.8	16.1	10.3	7.93	3.3	2.54
5	**10**	619.52	94	1.27	14.6	18.5	9.6	12.19	3.1	3.94
6	**1**	519.40	94	1.00	29	29	0.3	0.3	27.5	27.5

Cumulative PMI : 111
Cumulative PMI solvents : 40
Cumulative PMI water : 46

Sequence used in synthesis of intermediate **9**

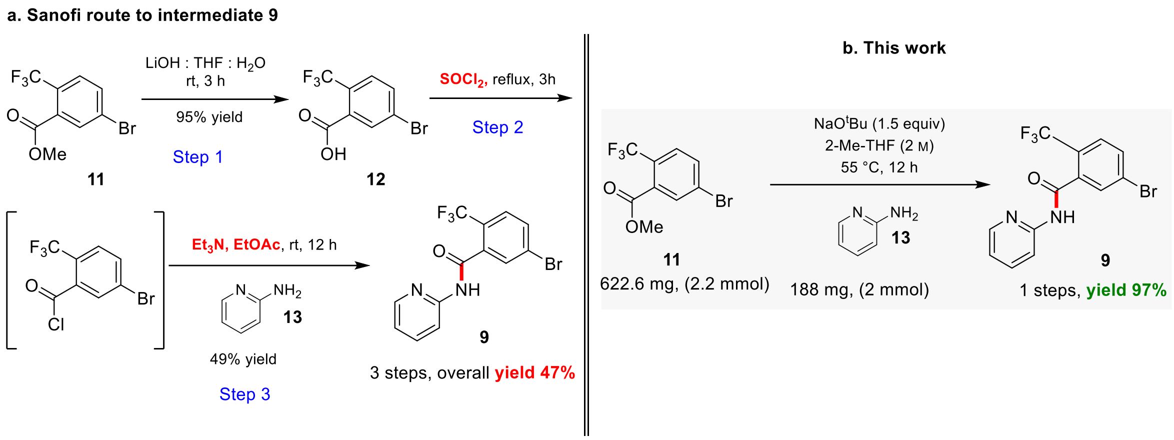

Literature route: Sanofi

Step 1:

	Material	Amount (g)
**Limiting Reagent Input**	compound **11**	9.1
**Product Output**	compound **12**	8.0
**Reagents**	**LiOH**	4.4
**Solvents**	tetrahydrofuran	17.6
**Aqueous**	H_2O	10
	aq. citric acid	30
	water wash	30
**Totals**	all materials	92
	reagents	4
	solvents	18
	water	70

Step PMI : 12.6
Step PMI solvents : 2.2
Step PMI water : 8.8

Step 2/Step3:

	Material	Amount (g)
**Limiting Reagent Input**	compound **12**	8.0
**Product Output**	compound **9**	5.1
**Reagents**	thionyl chloride	65.6
	2-aminopyridine (**13**)	3.2
	triethylamine	9.03
**Solvents**	ethyl acetate	433
**Aqueous**	water	400
	brine	200

**Totals**	all materials	1118.8
	reagents	77.83
	solvents	433
	water	600

Step PMI : 219
Step PMI solvents : 85
Step PMI water : 118

This work: synthesis of Intermediate 9

	Material	Amount (g)
**Limiting Reagent Input**	compound **13**	0.188
**Product Output**	compound **9**	0.669
**Reagents**	compound **11**	0.622
	NaO^tBu	0.192
**Solvents**	2-MeTHF	0.854
	ethyl acetate	1.66
**Aqueous**	water	0.5
**Totals**	all materials	3.824
	reagents	0.622
	solvents	2.514
	water	0.5

Step PMI : **6**
Step PMI solvents : **3.8**
Step PMI water : **0.7**

Step	*Step product*	*MW*	*Yield (%)*	*Usage factor (g)**	*Step PMI*	*Cumulative PMI contribution*	*Step PMI solvents*	*Cumulative PMI contribution solvents*	*Step PMI water*	*Cumulative PMI contribution water*
1	**9**	283.04	95	1.673	12.6	48.24	2.2	3.68	8.8	14.72
2	**9**	345.12	49	1.00	219	219	85	85	118	118

Cumulative PMI : 267.24
Cumulative PMI solvents : 88.68
Cumulative PMI water : 132.72

5. ICP-MS data for residual palladium

ICP-MS analysis of MMM688533 (**1**), Intermediate **14**, and Intermediate **10** were performed by the UCLA Nano and Pico (NPC) Laboratory in order to determine the amount of residual palladium present in the final drug, and was shown to be below the detectable limit (see Table S5).

		**palladium**	
		[µg/g]	
Sample #	Sample weight in analysis [mg]	Average*	St dev
MMV688533 (**1**)	2.50	8.455	0.071
Intermediate **16**	10.00	0.520	0.010
Int-**10** (This work)	2.00	3.536	0.061
Int-**10** (literature)	8.00	3759.832	40.000

**Each sample was done in triplicated measurements with background correction; n/a represents below detection limit.*

6. Analytical data

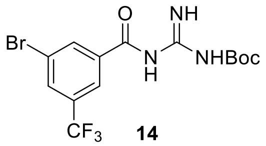

14

t-Butyl *N*-[*N*-(3-(trifluoromethyl)-5-bromo-benzoyl) carbamimidoyl] carbamate (14): ^{1}H NMR (400 MHz, CDCl$_3$) δ 9.64 (bs, 1H), 8.77 (s, 1H), 8.47 (s, 1H), 8.35 (s, 1H), 7.86 (s, 1H), 1.43 (s, 9H). **^{13}C NMR (101 MHz, CDCl$_3$)** δ 175.4, 169.6, 159.6, 153.3, 139.9, 135.6, 131.3 (q, *J* = 3.6 Hz), 124.9 (q, *J* = 3.9 Hz), 122.7, 84.1, 28.0. **^{19}F NMR (376 MHz, CDCl$_3$)** δ -62.8. **HRMS** (ESI–, m/z, [M–H]$^+$): calcd. for $C_{14}H_{16}BrF_3N_3O_3$ 408.0170; found 408.0174. **R$_f$** (SiO$_2$, hexanes/EtOAc 85:15) = 0.55.

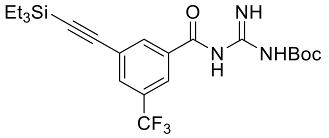

15

***t*-Butyl *N*-[*N*-[3-((triethylsilyl)ethynyl)-5-(trifluoromethyl) benzoyl] carbamimidoyl] carbamate (15): ^{1}H NMR (400 MHz, CDCl$_3$)** δ 9.42 (bs, 1H), 8.67 (s, 1H), 8.41 (s, 1H), 8.36 (s, 1H), 7.80 (s, 1H), 1.40 (s, 9H), 1.03 (t, *J* = 7.7 Hz, 9H), 0.67 (q, *J* = 7.7 Hz, 6H). **^{13}C NMR (101 MHz, CDCl$_3$)** δ 176.6, 159.6, 153.1, 138.6, 135.9, 131.3, 125.9, 124.4, 123.95 (q, *J* = 272.5 Hz), 104.3, 94.2, 84.0, 28.0, 7.6, 4.4. **^{19}F NMR (376 MHz, CDCl$_3$)** δ -62.9. **HRMS** (ESI+, m/z, [M+H]$^+$) : calcd. for $C_{22}H_{31}F_3N_3O_3Si$, 470.2087; found 470.2094; **R$_f$** (SiO$_2$, hexanes/EtOAc 90:10) = 0.35.

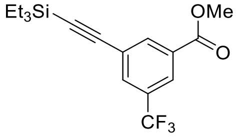

16

Methyl 3-((triethylsilyl)ethynyl)-5-(trifluoromethyl) benzoate (16): ^{1}H NMR (400 MHz, CDCl$_3$) δ 8.28 (t, *J* = 1.7 Hz, 1H), 8.21 (td, *J* = 1.7, 0.9 Hz, 1H), 7.87 (td, *J* = 1.6, 0.8 Hz, 1H), 3.96 (s, 3H), 1.06 (t, *J* = 7.9 Hz, 9H), 0.70 (q, *J* = 7.8 Hz, 6H). **^{13}C NMR (126 MHz, CDCl$_3$)** δ 165.2, 136.2, 132.71 (q, *J* = 3.7 Hz), 131.46 (q, *J* = 33.5 Hz), 131.4, 129.8, 129.0, 126.05 (q, *J* = 3.8 Hz), 125.1, 123.38 (q, *J* = 272.8 Hz), 103.5, 95.5,52.8, 7.6, 4.4. **^{19}F NMR (376 MHz, CDCl$_3$)**

δ -63.0. **HRMS** (GC-ESI+, *m/z*, $[M]^+$): calcd. for $C_{17}H_{21}F_3O_2Si$, 342.1262; found 342.1263. **R_f** (SiO_2, hexanes/EtOAc 95:05) = 0.40.

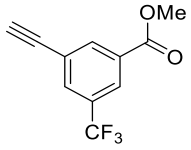

4

Methyl 3-ethynyl-5-(trifluoromethyl)benzoate (4): ^{1}H NMR (400 MHz, $CDCl_3$) δ 8.32 (t, *J* = 1.6 Hz, 1H), 8.26 (d, *J* = 1.9 Hz, 1H), 7.91 (d, *J* = 1.9 Hz, 1H), 3.97 (s, 3H), 3.21 (s, 1H). **^{13}C NMR (101 MHz, $CDCl_3$)** δ 165.1, 136.3, 132.86 (q, *J* = 3.7 Hz), 131.60 (q, *J* = 33.3 Hz), 131.6, 126.59 (q, *J* = 3.8 Hz), 123.9, 123.48 (q, *J* = 272.4 Hz), 81.2, 80.1, 52.8. **^{19}F NMR (376 MHz, $CDCl_3$)** δ -63.1. **HRMS** (GC-ESI^+, *m/z*, $[M+H]^+$): calcd. for $C_{11}H_7F_3O_2$, 228.0398; found 228.0398. **R_f** (SiO_2, hexanes/EtOAc 95:05) = 0.38.

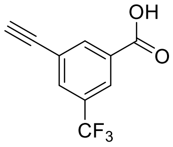

5

3-Ethynyl-5-(trifluoromethyl)benzoic acid (5) : ^{1}H NMR (500 MHz, DMSO) δ 13.82 (bs, 1H), 8.20 (d, *J* = 1.8 Hz, 1H), 8.16 (s, 1H), 8.09 (d, *J* = 2.4 Hz, 1H), 4.53 (s, 1H). **^{13}C NMR (126 MHz, DMSO)** δ 165.2, 135.9, 133.1, 131.94 (q, *J* = 3.8 Hz), 130.16 (q, *J* = 32.8 Hz), 125.71 (q, *J* = 3.7 Hz), 123.6, 123.31 (q, *J* = 273.1 Hz), 83.7, 81.0. **^{19}F NMR (471 MHz, DMSO-d_6)** δ -61.6. **HRMS** (ESI^+, *m/z*, $[M-H]^+$): calcd. for $C_{10}H_4F_3O_2$, 213.0163; found 213.0174; **R_f** (SiO_2, hexanes/EtOAc 60:40) = 0.40.

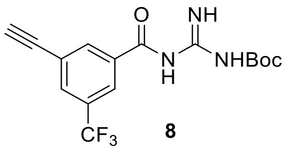

8

***t*-Butyl *N*-[*N*-[3-ethynyl-5-(trifluoromethyl)benzoyl]carbamimidoyl]carbamate (8): ^{1}H NMR (400 MHz, $CDCl_3$)** δ 9.06 (s, 1H), 8.78 (s, 1H), 8.60 (s, 1H), 8.45 (d, *J* = 1.6 Hz, 1H), 8.41 (d, *J* = 2.0 Hz, 1H), 7.83 (d, *J* = 1.8 Hz, 1H), 3.15 (s, 1H), 1.51 (s, 9H). **^{13}C NMR (126 MHz, $CDCl_3$)**

δ 176.3, 159.63 (d, J = 2.8 Hz), 153.1, 138.8, 136.0, 131.54 (q, J = 3.7 Hz), 130.90 (q, J = 33.0 Hz), 126.36 (q, J = 3.7 Hz), 123.54 (d, J = 272.9 Hz) 123.2, 84.0, 81.9, 79.1, 27.9. **^{19}F NMR (376 MHz, CDCl$_3$)** δ -62.9. **HRMS** (ESI$^+$, *m/z*, [M+H]$^+$): calcd. for $C_{16}H_{17}F_3N_3O_3$, 356.1222; found 356.1214; **R$_f$** (SiO$_2$, hexanes/EtOAc 70:30) = 0.45.

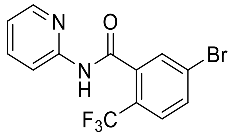

9

5-Bromo-*N*-(pyridin-2-yl)-2-(trifluoromethyl)benzamide (9): ^{1}H NMR (400 MHz, CDCl$_3$) δ 8.98 (s, 1H), 8.36 (d, J = 8.4 Hz, 1H), 8.15 (s, 1H), 7.83 (s, 2H), 7.75 (d, J = 8.4 Hz, 1H), 7.62 (d, J = 8.4 Hz, 1H), 7.12 (s, 1H). **^{13}C NMR (126 MHz, CDCl$_3$)** δ 164.8, 151.4, 146.9, 139.1, 137.2, 133.4, 131.6, 128.35 (q, J = 4.8 Hz), 126.7, 126.60 (q, J = 31.7 Hz), 121.77 (q, J = 274.7 Hz), 120.4, 115.1. **^{19}F NMR (376 MHz, CDCl$_3$)** δ -59.0. **HRMS** (ESI$^+$, *m/z*, [M+Na]$^+$): calcd. for $C_{13}H_8BrF_3N_2ONa$, 366.9670; found 366.9675; **R$_f$** (SiO$_2$, hexanes/EtOAc 70:20) = 0.42.

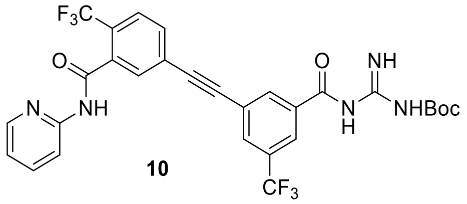

10

***t*-Butyl *N*-[*N*-[3-[2-[3-(2-pyridylcarbamoyl)-4-(trifluoromethyl) phenyl] ethynyl] -5-(trifluoromethyl) benzoyl] carbamimidoyl] carbamate (10): ^{1}H NMR (500 MHz, CDCl$_3$)** δ 10.94 (s, 1H), 10.32 (s, 1H), 9.82 (s, 1H), 8.79 (d, J = 4.7 Hz, 1H), 8.40 – 8.32 (m, 2H), 8.21 (dt, J = 8.7, 1.7 Hz, 2H), 7.90 – 7.85 (m, 1H), 7.85 – 7.81 (m, 1H), 7.66 – 7.57 (m, 2H), 7.20 – 7.14 (m, 1H), 7.04 (s, 1H), 1.52 (s, 9H). **^{13}C NMR (126 MHz, CDCl$_3$)** δ 175.3, 165.6, 160.7, 153.8, 151.4, 147.4, 139.4, 138.1, 135.4 (q, J = 2.4 Hz), 135.3, 132.8, 130.9 (q, J = 3.4 Hz), 130.9 (q, J = 33.4 Hz), 126.8, 126.6 (q, J = 4.5 Hz), 126.3 (q, J = 3.5 Hz), 125.85 (q, J = 31.7 Hz), 124.56 (d, J = 13.3 Hz), 124.1, 122.39 (d, J = 14.3 Hz), 120.4, 115.7, 91.6, 89.0, 83.7, 28.2. **^{19}F NMR (471 MHz, CDCl$_3$)** δ -59.2, -63.0. **HRMS** (ESI$^+$, *m/z*, [M+Na]$^+$): calcd. for $C_{29}H_{23}F_6N_5O_4Na$, 642.1552; found 642.1556; **R$_f$** (SiO$_2$, hexanes/EtOAc 70:40) = 0.55.

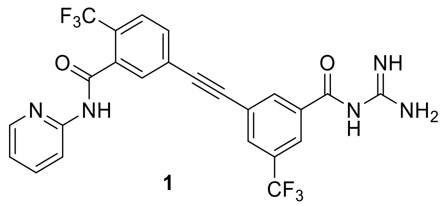

1

5-((3-(Carbamimidoylcarbamoyl)-5- (trifluoromethyl)phenyl)ethynyl) -*N*-(pyridin-2-yl)-2-(trifluoromethyl) benzamide (**1**): **^{1}H NMR (400 MHz, DMSO-d$_6$)** δ 11.18 (s, 1H), 8.51 (d, *J* = 1.6 Hz, 1H), 8.42 – 8.34 (m, 2H), 8.16 (d, *J* = 8.4 Hz, 1H), 8.09 (d, *J* = 1.9 Hz, 1H), 7.98 (s, 1H), 7.93 – 7.83 (m, 3H), 7.19 (dd, *J* = 7.3, 4.8 Hz, 1H). **^{13}C NMR (126 MHz, DMSO-d$_6$)** δ ^{13}C NMR (126 MHz, DMSO) δ 172.93, 165.89, 163.59, 152.13, 148.63, 141.31, 138.81, 136.57, 135.49, 133.14, 132.22, 130.37, 130.33, 130.31, 130.12, 129.87, 129.61, 127.49, 127.45, 127.41, 127.38, 127.22, 126.65, 126.40, 126.31, 126.14, 125.91, 125.88, 125.85, 125.23, 125.04, 123.06, 122.93, 122.87, 120.90, 120.69, 120.65, 114.64, 91.12, 89.25, 40.57, 40.48, 40.41, 40.32, 40.24, 40.15, 40.07, 39.98, 39.90, 39.82, 39.65, 39.48. **^{19}F NMR (376 MHz, DMSO-d$_6$)** δ -58.1, -61.4. **HRMS** (ESI$^+$, *m/z*, [M+Na]$^+$): calcd. for $C_{24}H_{15}F_6N_5O_2Na$, 542.1028; found 542.1014; **mp** = 153-156 °C. **R$_f$** (SiO_2, MeOH/CH_2Cl_2 10:90) = 0.48.

7. References

1. B. H. Lipshutz, S. Ghorai, A. R. Abela, R. Moser, T. Nishikata, C. Duplais and A. Krasovskiy, *J. Org. Chem.*, 2011, **76**, 4379-4391.

2. WO2019008027A1 (PCT/EP2018/068079).

8. HPLC data

Column	:	Lux cellulose-2, 256 x 4.6 mm
Eluent	:	hexane/ i-PrOH = 85/15
Flow rate	:	1.25 mL/min
Column temperature	:	20 °C

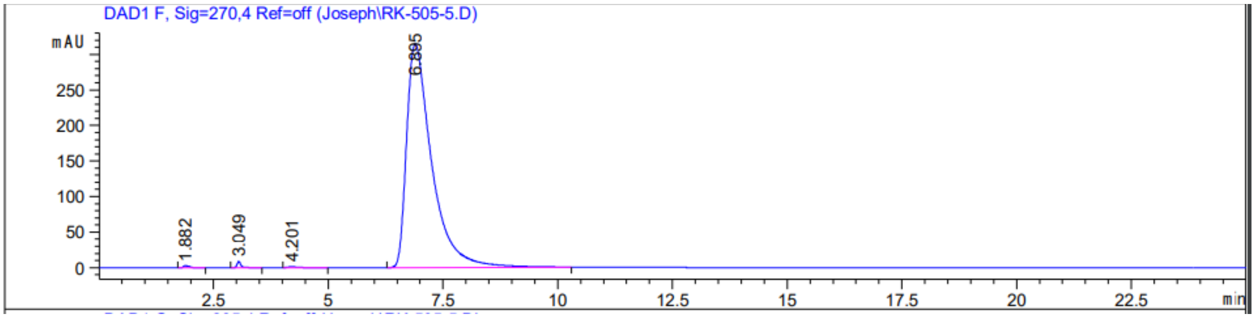

Signal 6: DAD1 F, Sig=270,4 Ref=off

Peak #	RetTime [min]	Type	Width [min]	Area [mAU*s]	Height [mAU]	Area %
1	1.882	BB	0.1250	24.42749	2.71456	0.2020
2	3.049	BB	0.0906	52.52763	8.68288	0.4343
3	4.201	BB	0.1985	16.36362	1.21990	0.1353
4	6.895	BB	0.5691	1.20010e4	314.55920	99.2284

Totals : 1.20943e4 327.17655

9. NMR spectral data

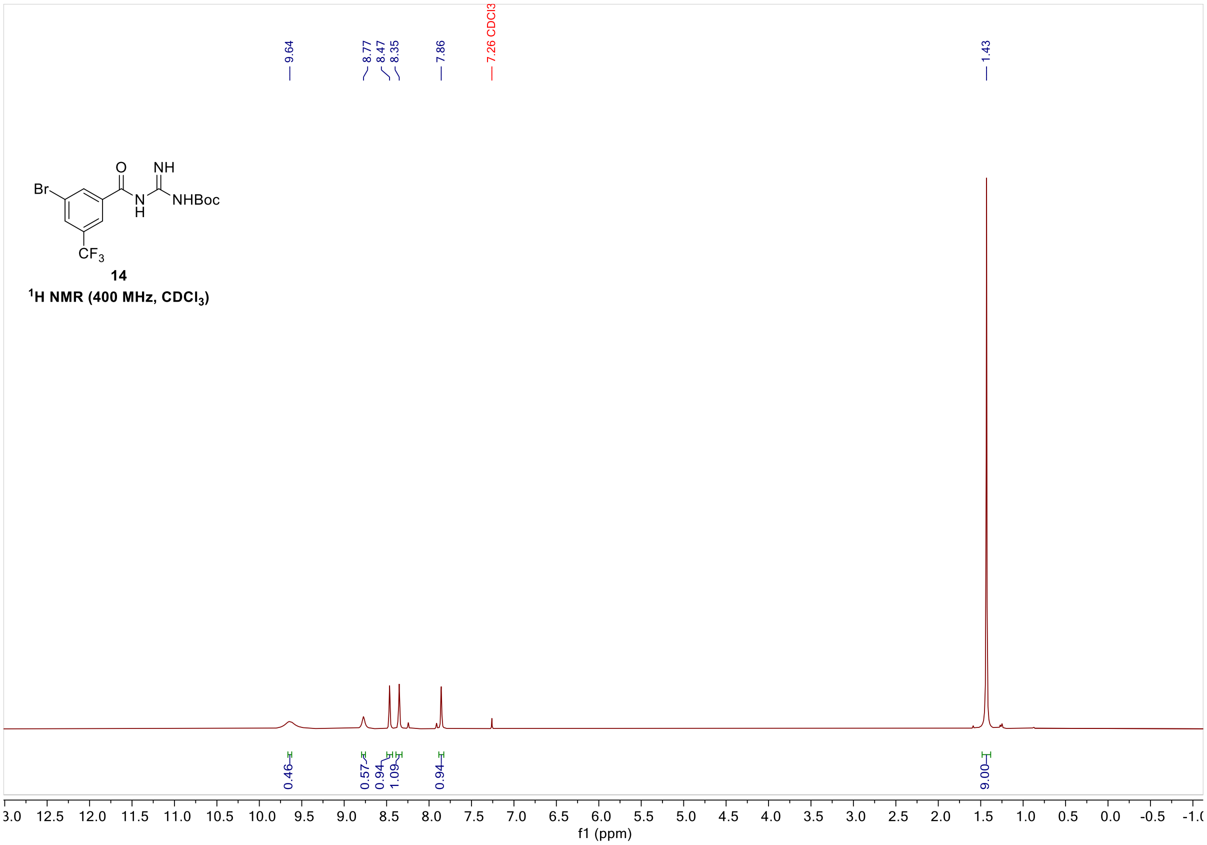

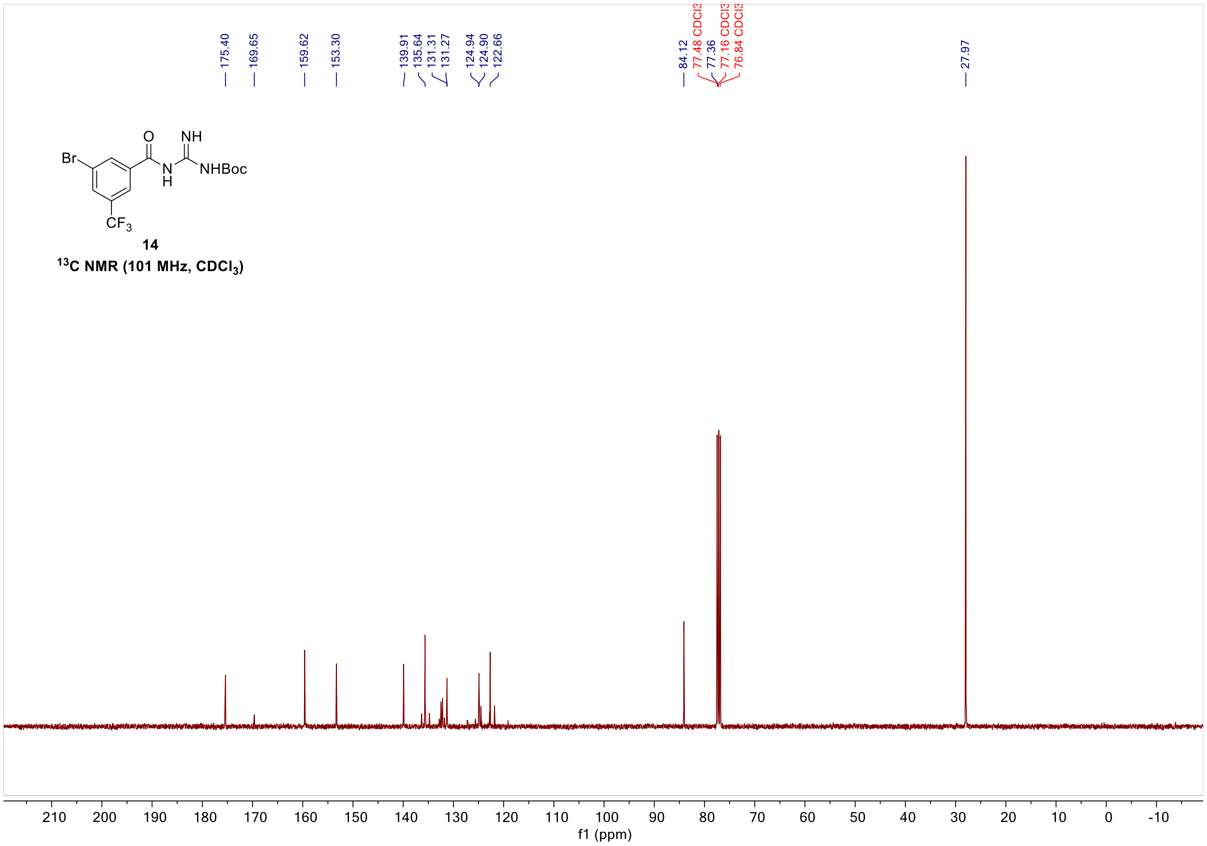

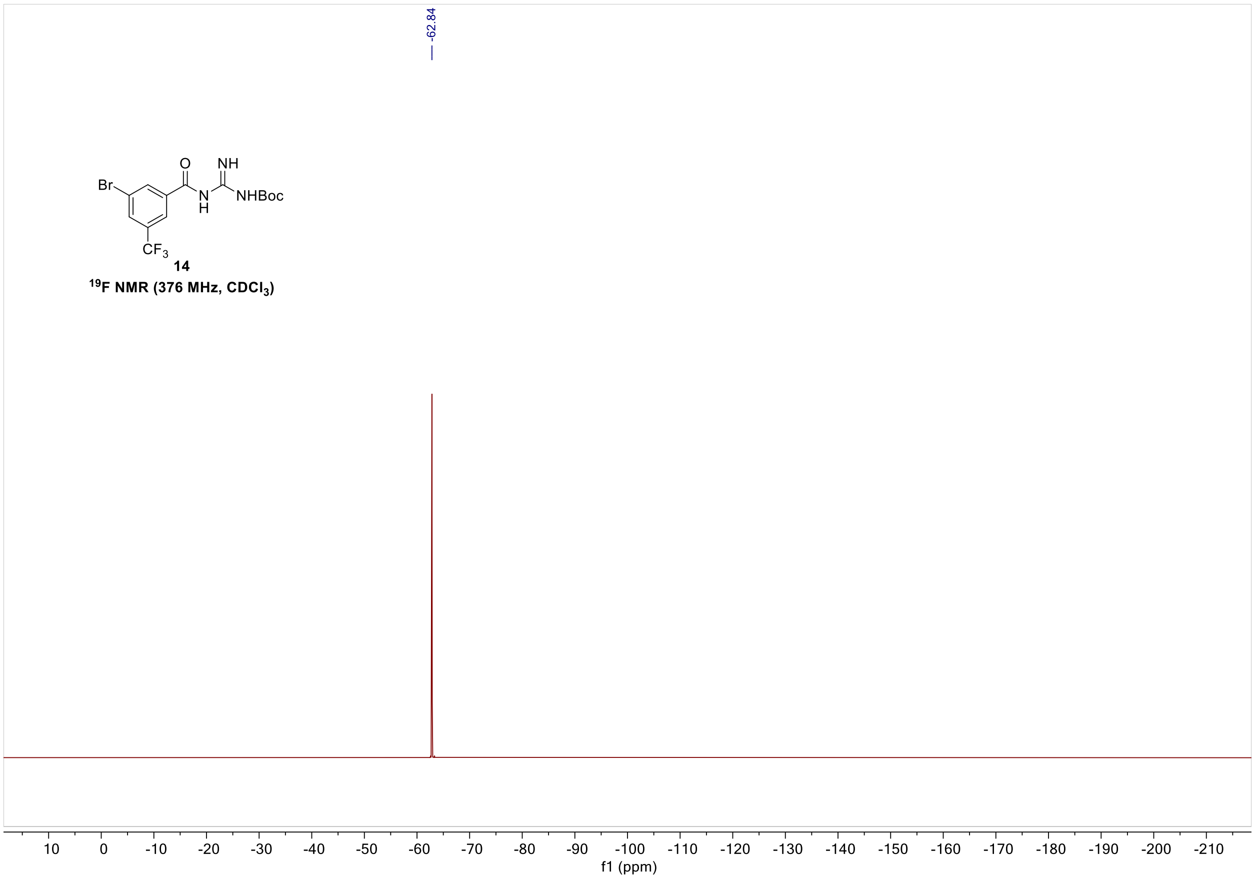

-62.84
Br
O
NH
N
H
NHBoc
CF3
14
19F NMR (376 MHz, CDCl3)
10 0 -10 -20 -30 -40 -50 -60 -70 -80 -90 -100 -110 -120 -130 -140 -150 -160 -170 -180 -190 -200 -210
f1 (ppm)

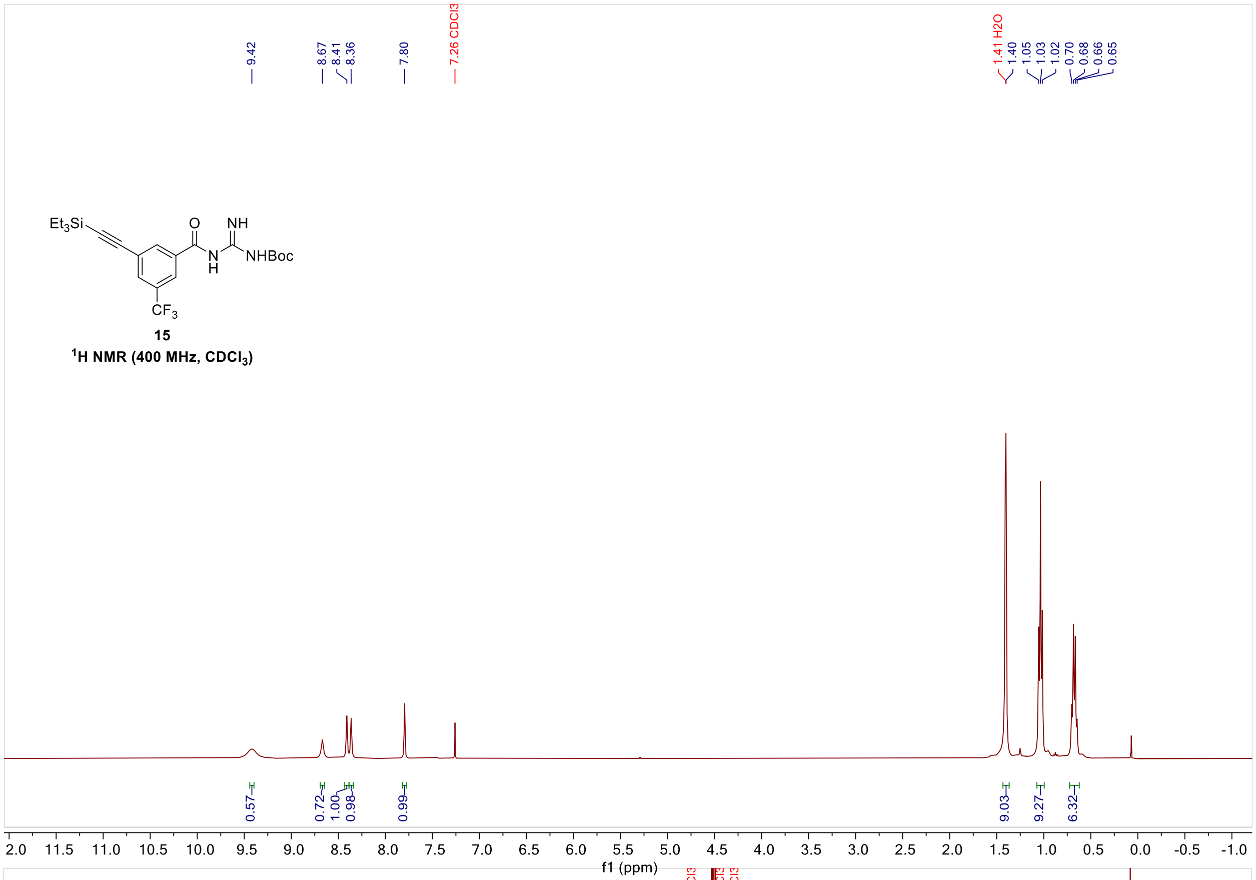
9.42
8.67
8.41
8.36
7.80
7.26 CDCl3
1.41 H2O
1.40
1.05
1.03
1.02
0.70
0.68
0.66
0.65
Et3Si
O
NH
N
H
NHBoc
CF3
15
1H NMR (400 MHz, CDCl3)
0.57
0.72
1.00
0.98
0.99
9.03
9.27
6.32
f1 (ppm)

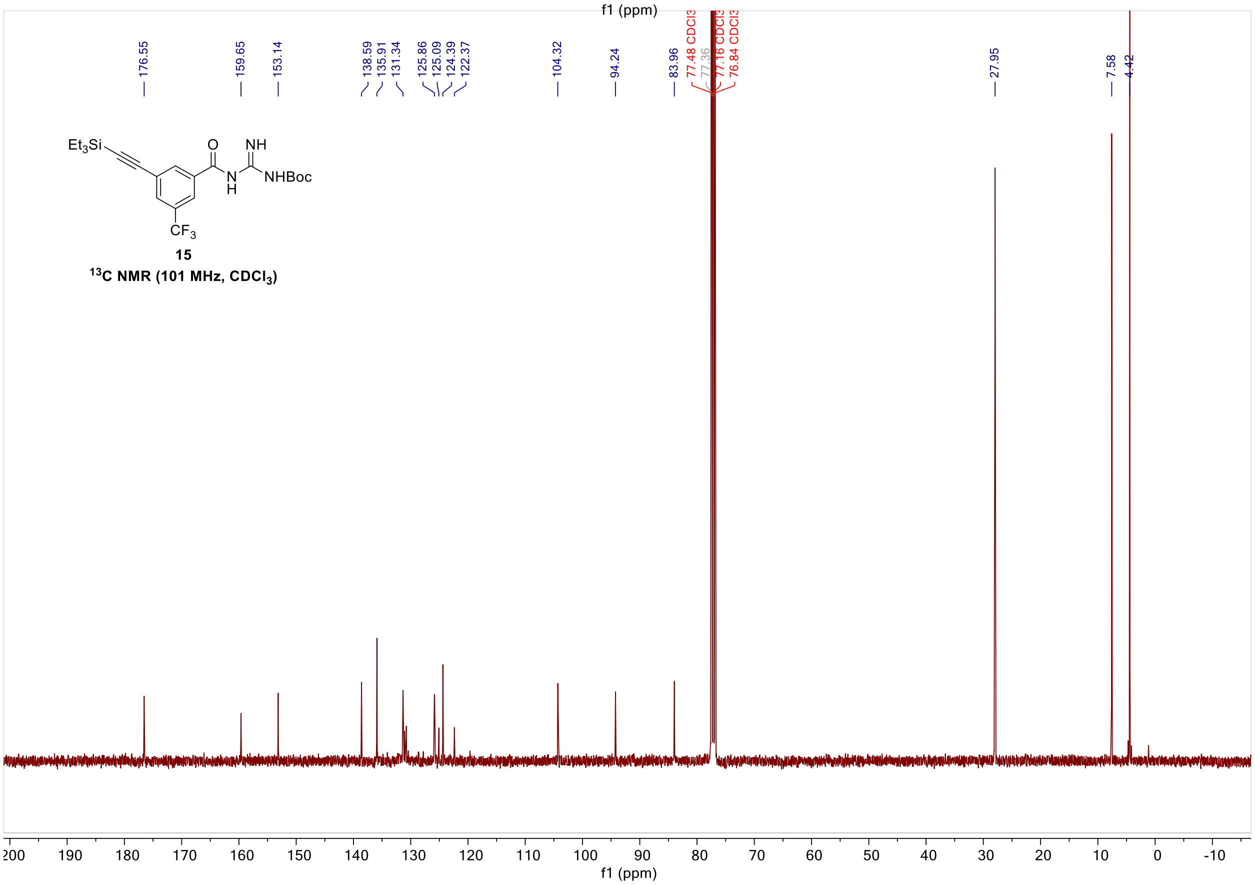
176.55
159.65
153.14
138.59
135.91
131.34
125.86
125.09
124.39
122.37
104.32
94.24
83.96
77.48 CDCl3
77.36
77.16 CDCl3
76.84 CDCl3
27.95
7.58
4.42
Et3Si
O
NH
N
H
NHBoc
CF3
15
13C NMR (101 MHz, CDCl3)
f1 (ppm)

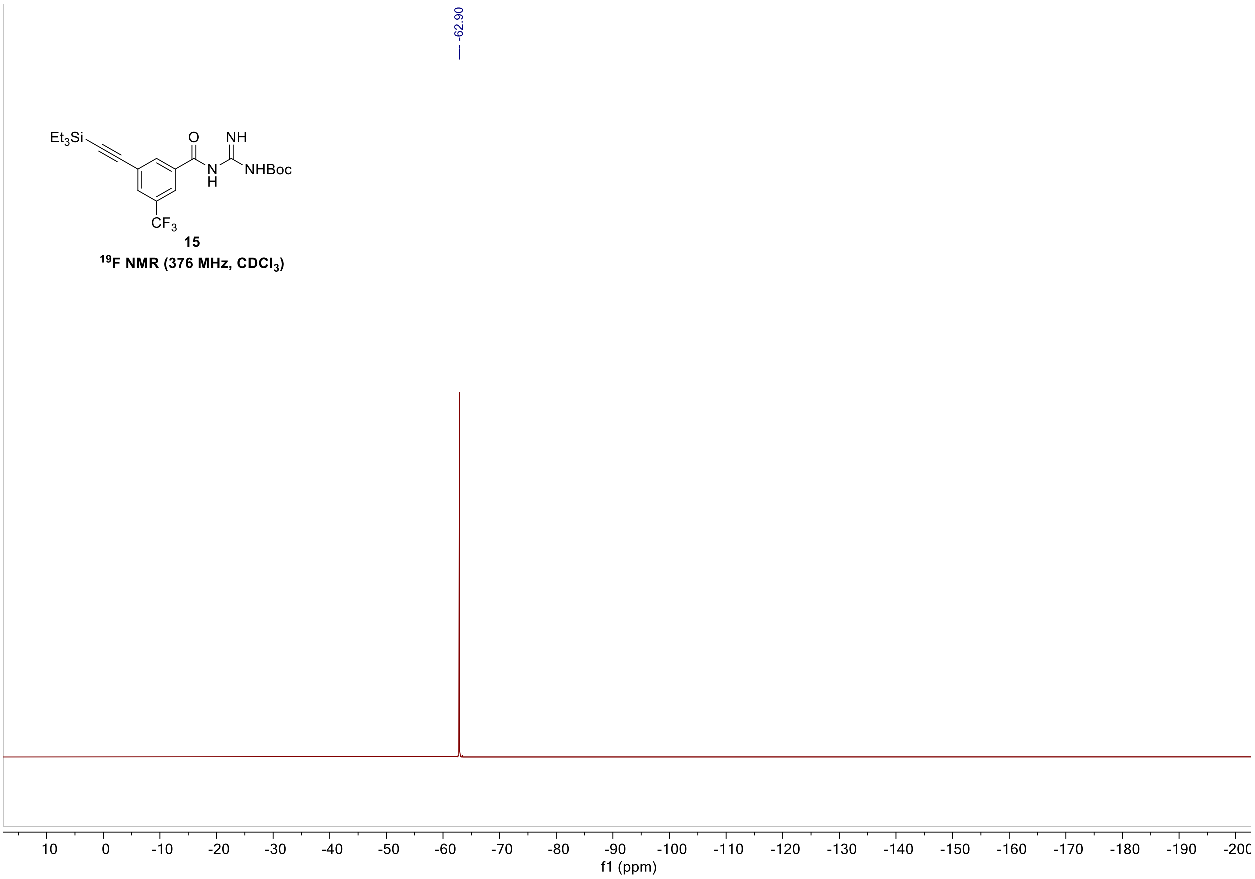
-62.90
Et3Si
O
NH
N
H
NHBoc
CF3
15
19F NMR (376 MHz, CDCl3)
10
0
-10
-20
-30
-40
-50
-60
-70
-80
-90
-100
-110
-120
-130
-140
-150
-160
-170
-180
-190
-200
f1 (ppm)

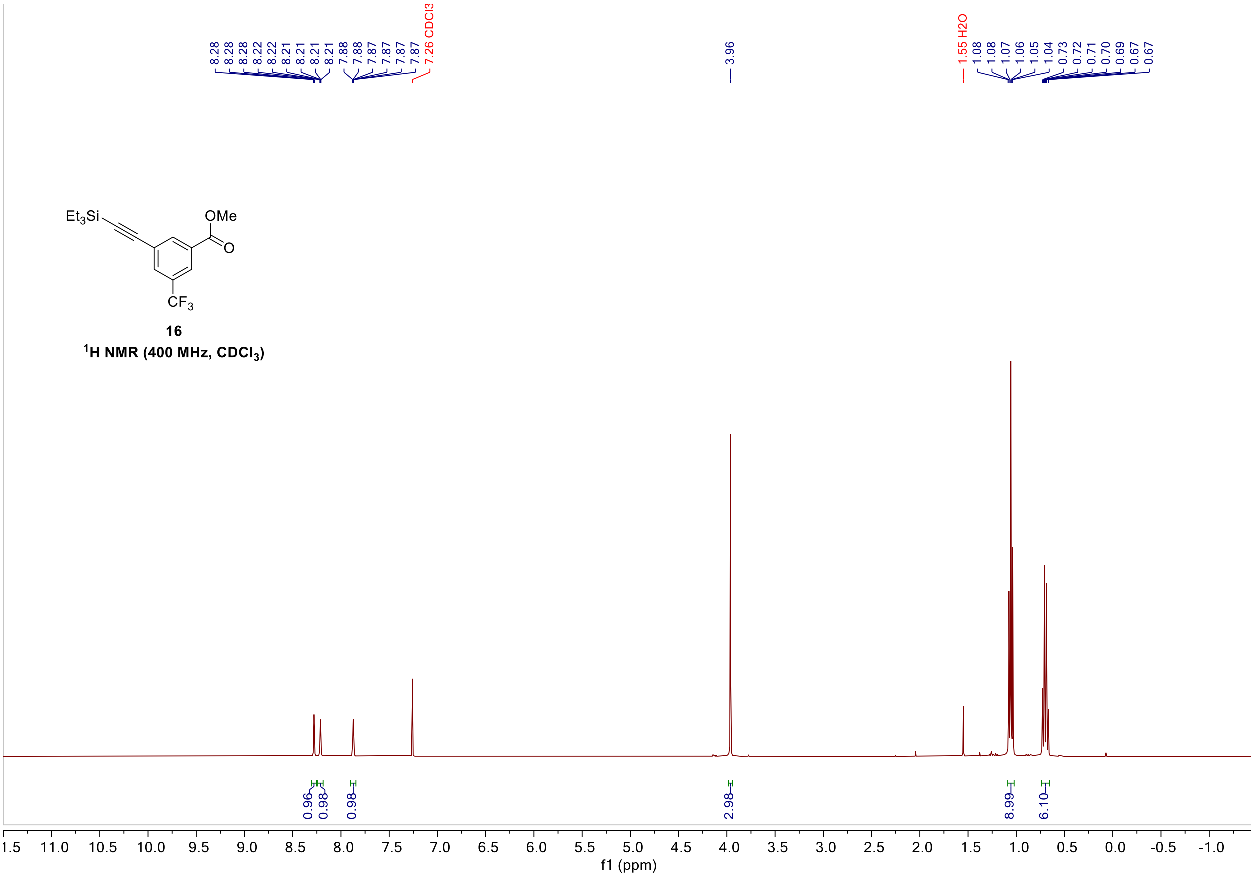
8.28 8.28 8.28 8.22 8.22 8.21 8.21 8.21 8.21 7.88 7.88 7.87 7.87 7.87 7.87 7.26 CDCl3
3.96
1.55 H2O 1.08 1.08 1.07 1.06 1.05 1.04 0.73 0.72 0.71 0.70 0.69 0.67 0.67
Et3Si
OMe
O
CF3
16
1H NMR (400 MHz, CDCl3)
0.96 0.98 0.98 2.98 8.99 6.10
f1 (ppm)

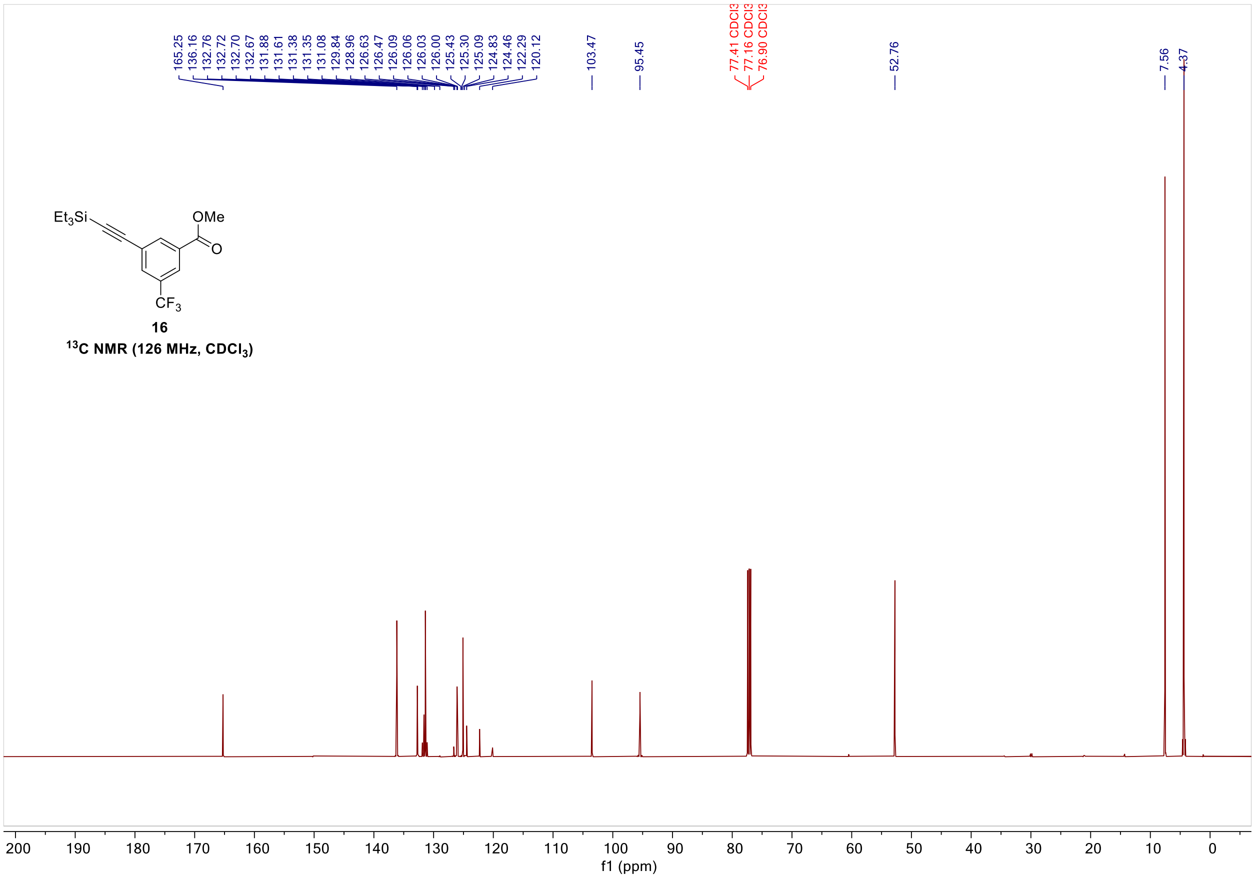
165.25 136.16 132.76 132.72 132.70 132.67 131.88 131.61 131.38 131.35 131.08 129.84 128.96 126.63 126.47 126.09 126.06 126.03 126.00 125.43 125.30 125.09 124.83 124.46 122.29 120.12
103.47
95.45
77.41 CDCl3 77.16 CDCl3 76.90 CDCl3
52.76
7.56
4.37
Et3Si
OMe
O
CF3
16
13C NMR (126 MHz, CDCl3)
f1 (ppm)

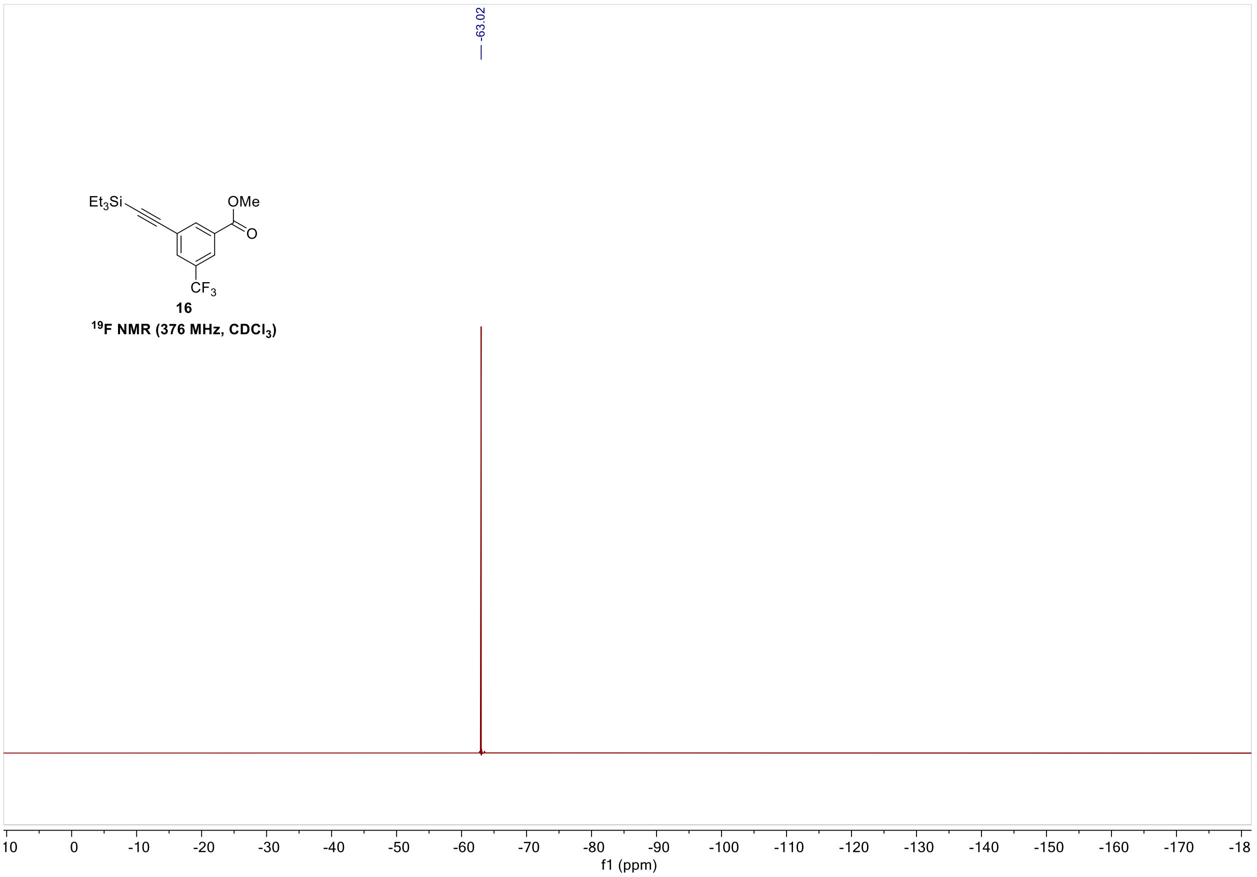
-63.02
Et3Si
OMe
O
CF3
16
19F NMR (376 MHz, CDCl3)
10
0
-10
-20
-30
-40
-50
-60
-70
-80
-90
-100
-110
-120
-130
-140
-150
-160
-170
-18
f1 (ppm)

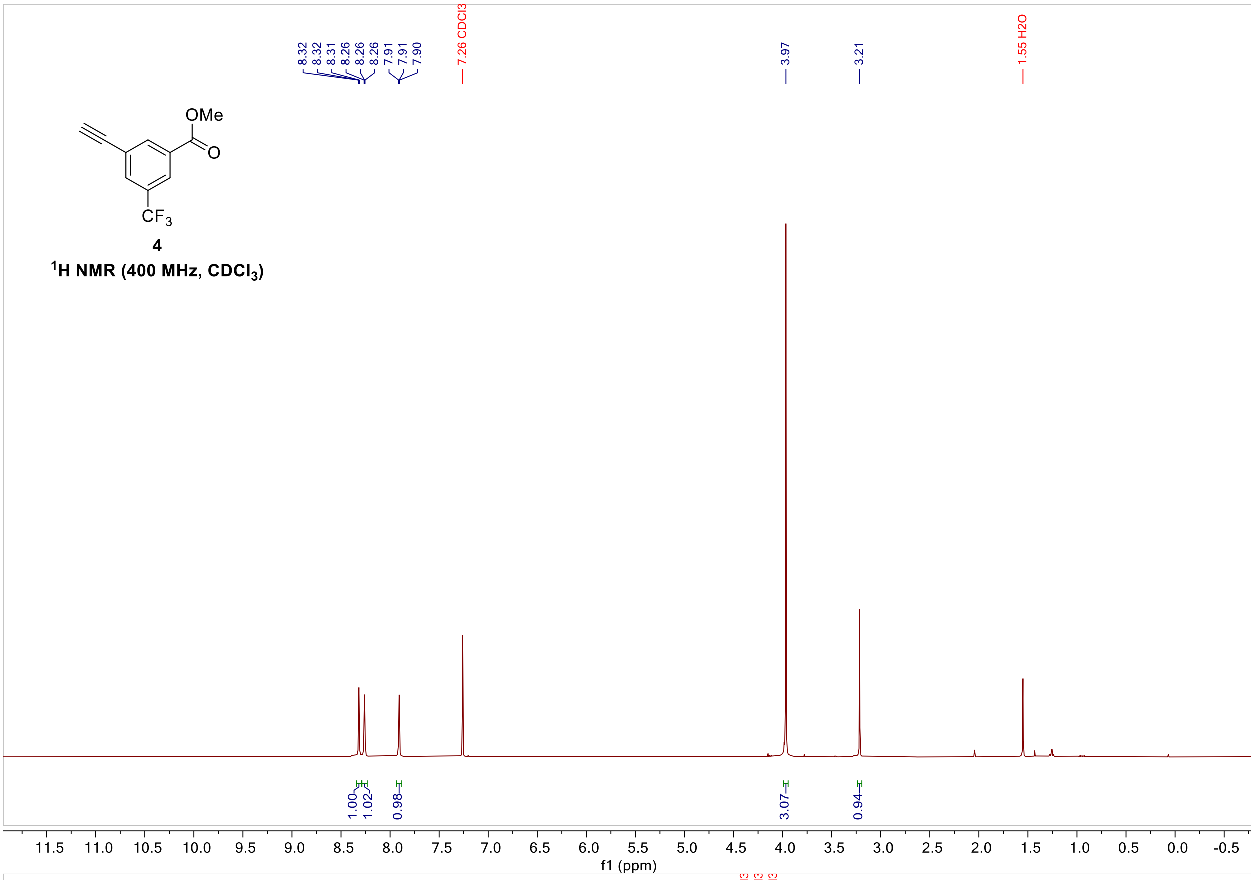

OMe
O
CF3
4
1H NMR (400 MHz, CDCl3)
8.32
8.32
8.31
8.26
8.26
8.26
7.91
7.91
7.90
7.26 CDCl3
3.97
3.21
1.55 H2O
1.00
1.02
0.98
3.07
0.94
f1 (ppm)

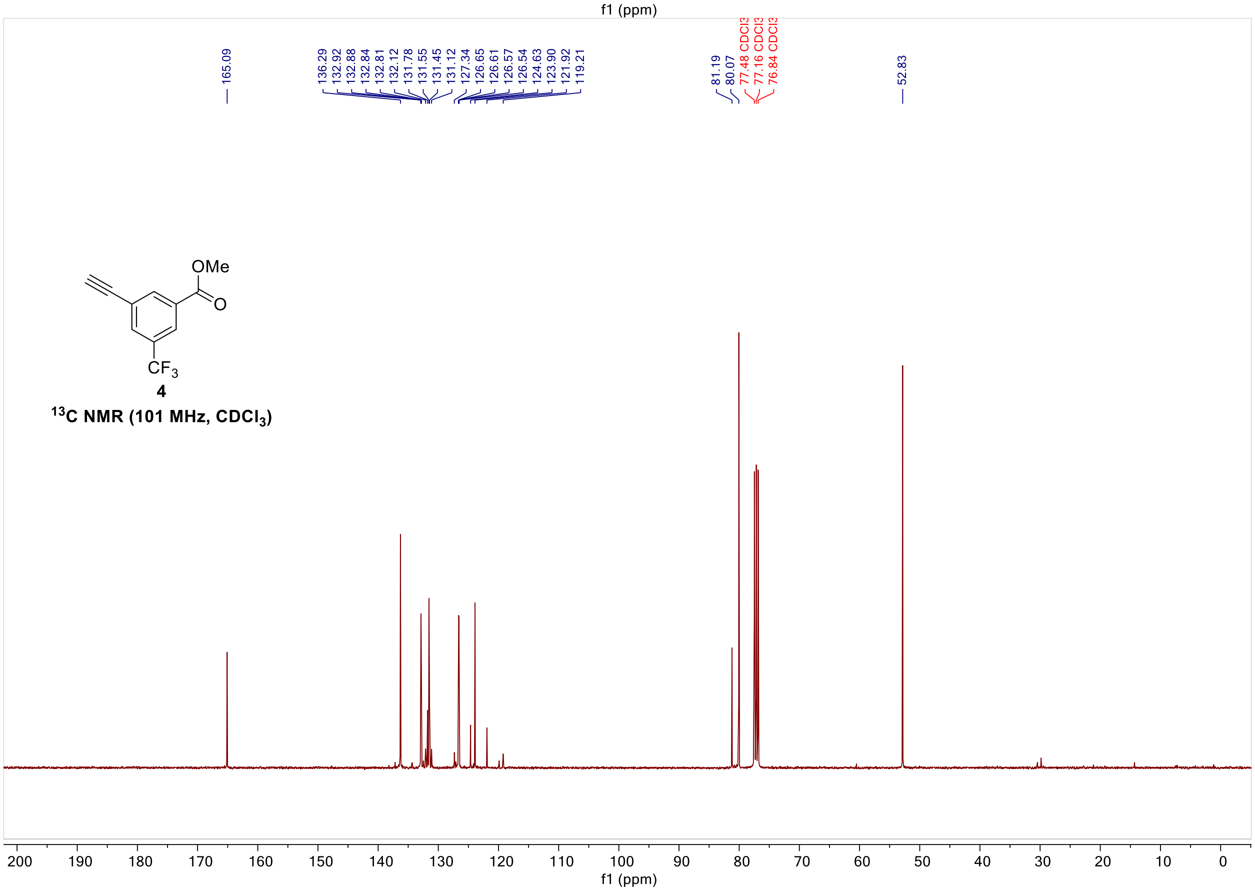

165.09
136.29
132.92
132.88
132.84
132.81
132.12
131.78
131.55
131.45
131.12
127.34
126.65
126.61
126.57
126.54
124.63
123.90
121.92
119.21
81.19
80.07
77.48 CDCl3
77.16 CDCl3
76.84 CDCl3
52.83
OMe
O
CF3
4
13C NMR (101 MHz, CDCl3)
f1 (ppm)

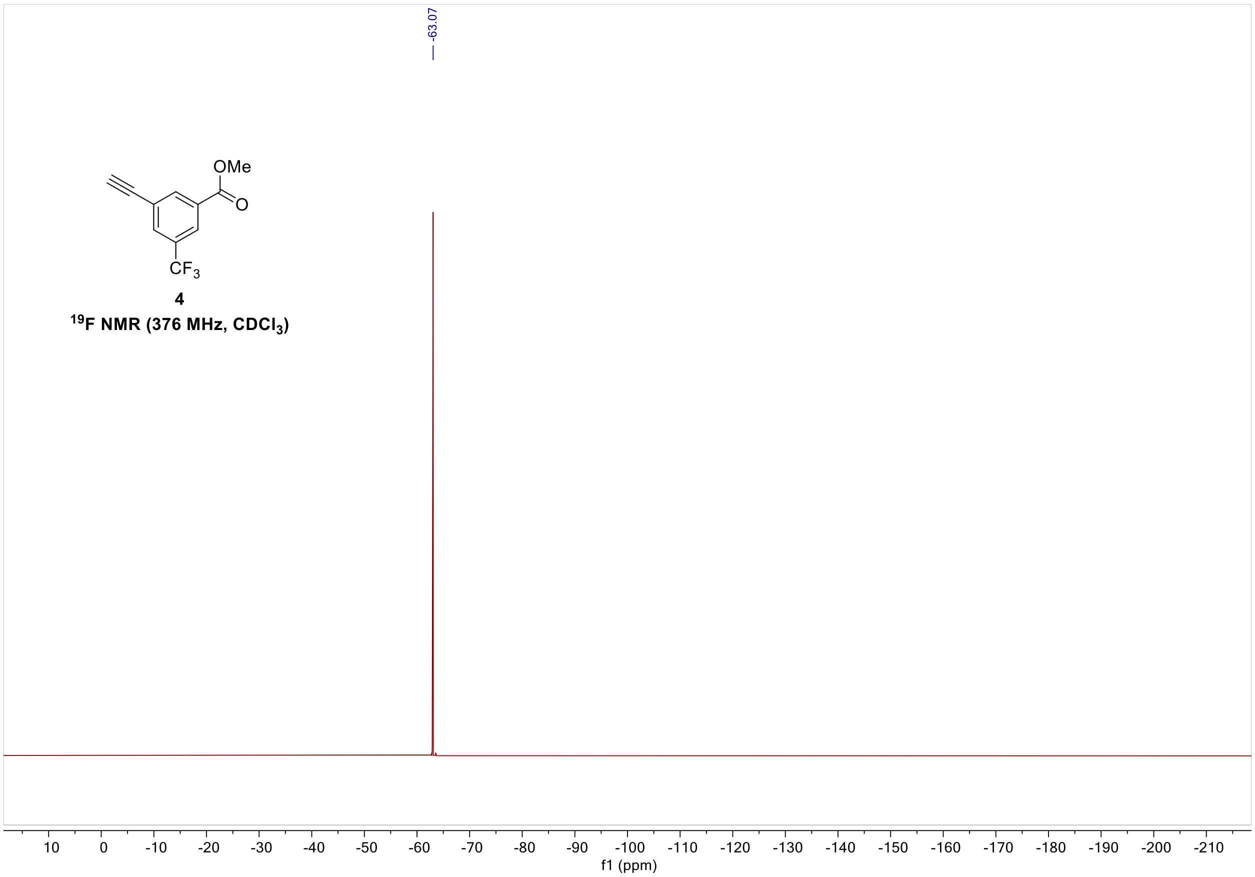
-63.07
OMe
O
CF3
4
19F NMR (376 MHz, CDCl3)
10 0 -10 -20 -30 -40 -50 -60 -70 -80 -90 -100 -110 -120 -130 -140 -150 -160 -170 -180 -190 -200 -210
f1 (ppm)

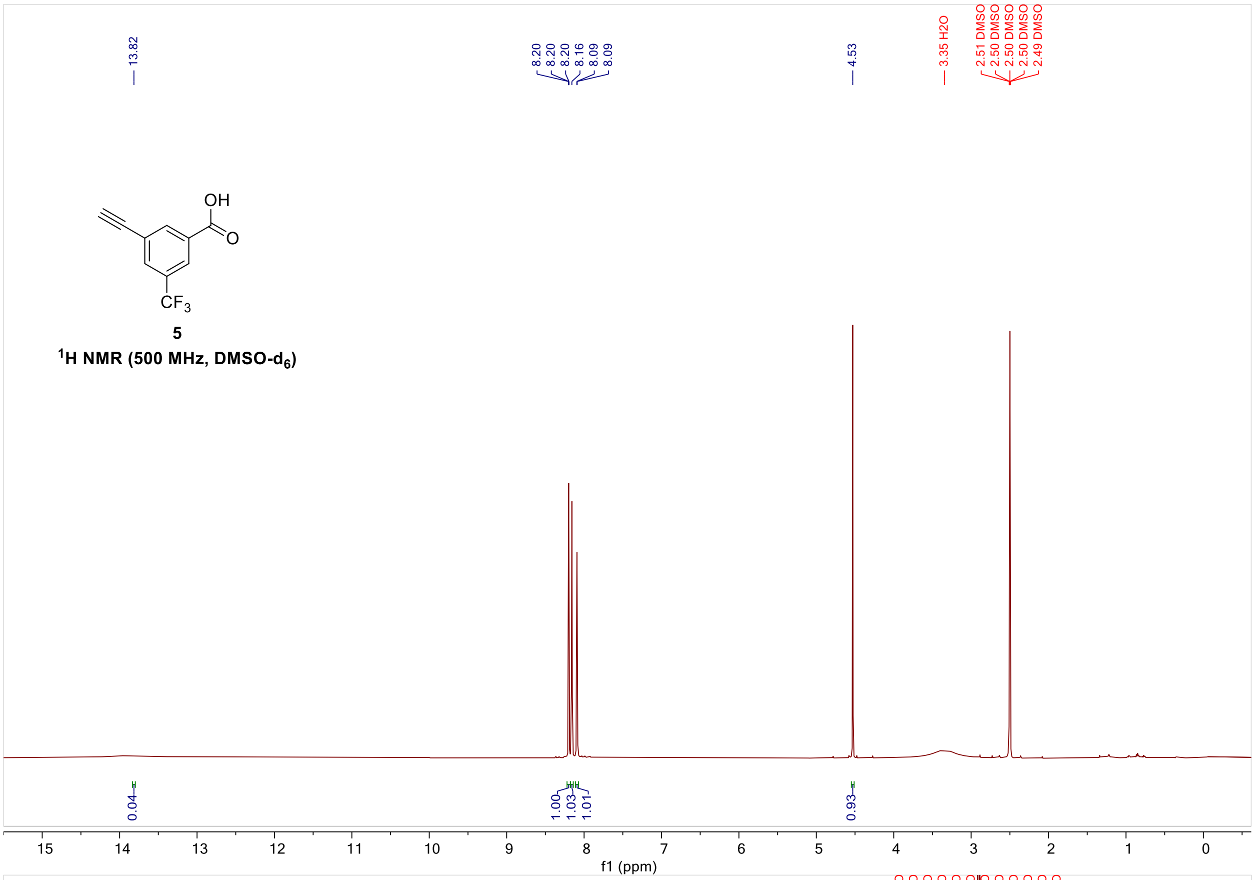
13.82
8.20
8.20
8.20
8.16
8.09
8.09
4.53
3.35 H2O
2.51 DMSO
2.50 DMSO
2.50 DMSO
2.50 DMSO
2.49 DMSO
OH
O
CF3
5
1H NMR (500 MHz, DMSO-d6)
0.04
1.00
1.03
1.01
0.93
15 14 13 12 11 10 9 8 7 6 5 4 3 2 1 0
f1 (ppm)

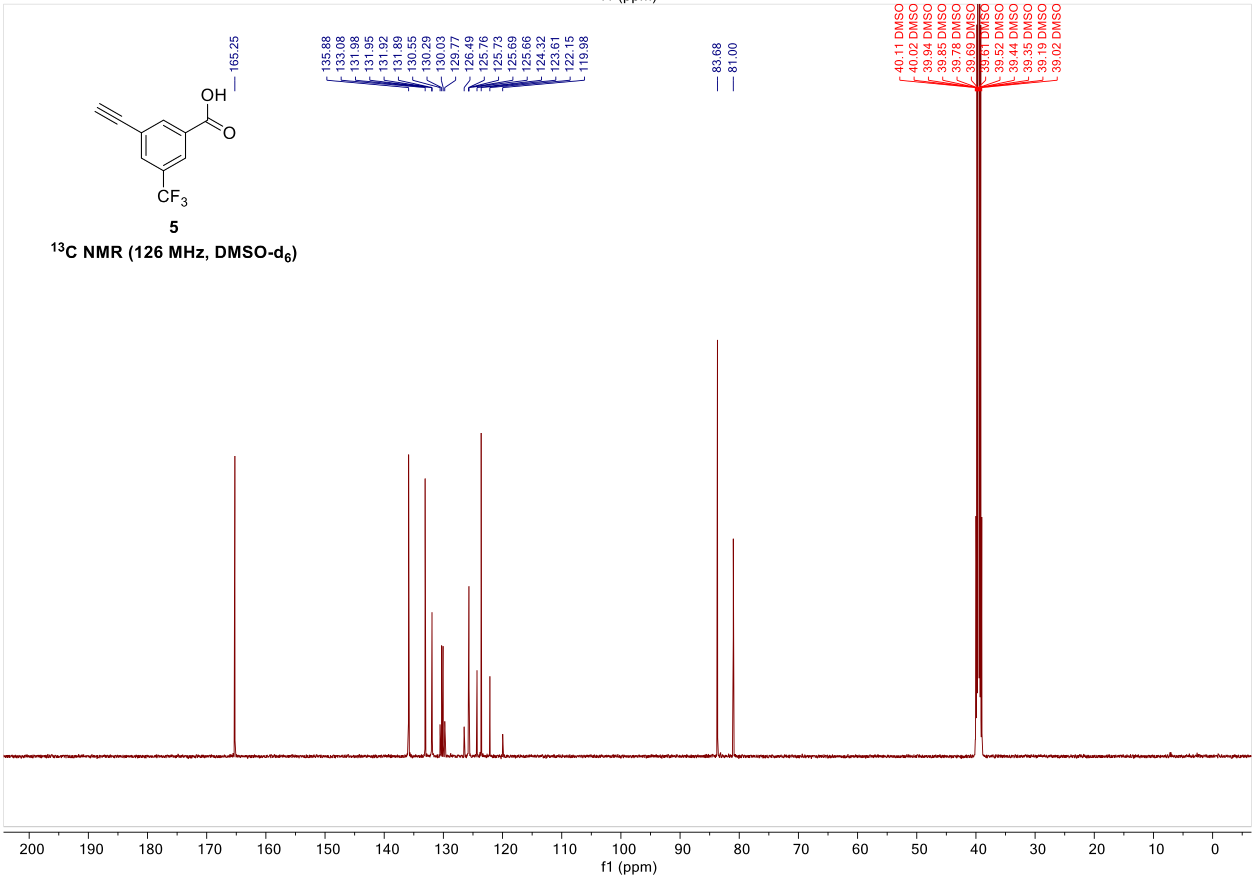
165.25
135.88
133.08
131.98
131.95
131.92
131.89
130.55
130.29
130.03
129.77
126.49
125.76
125.73
125.69
125.66
124.32
123.61
122.15
119.98
83.68
81.00
40.11 DMSO
40.02 DMSO
39.94 DMSO
39.85 DMSO
39.78 DMSO
39.69 DMSO
39.61 DMSO
39.52 DMSO
39.44 DMSO
39.35 DMSO
39.19 DMSO
39.02 DMSO
OH
O
CF3
5
13C NMR (126 MHz, DMSO-d6)
200 190 180 170 160 150 140 130 120 110 100 90 80 70 60 50 40 30 20 10 0
f1 (ppm)

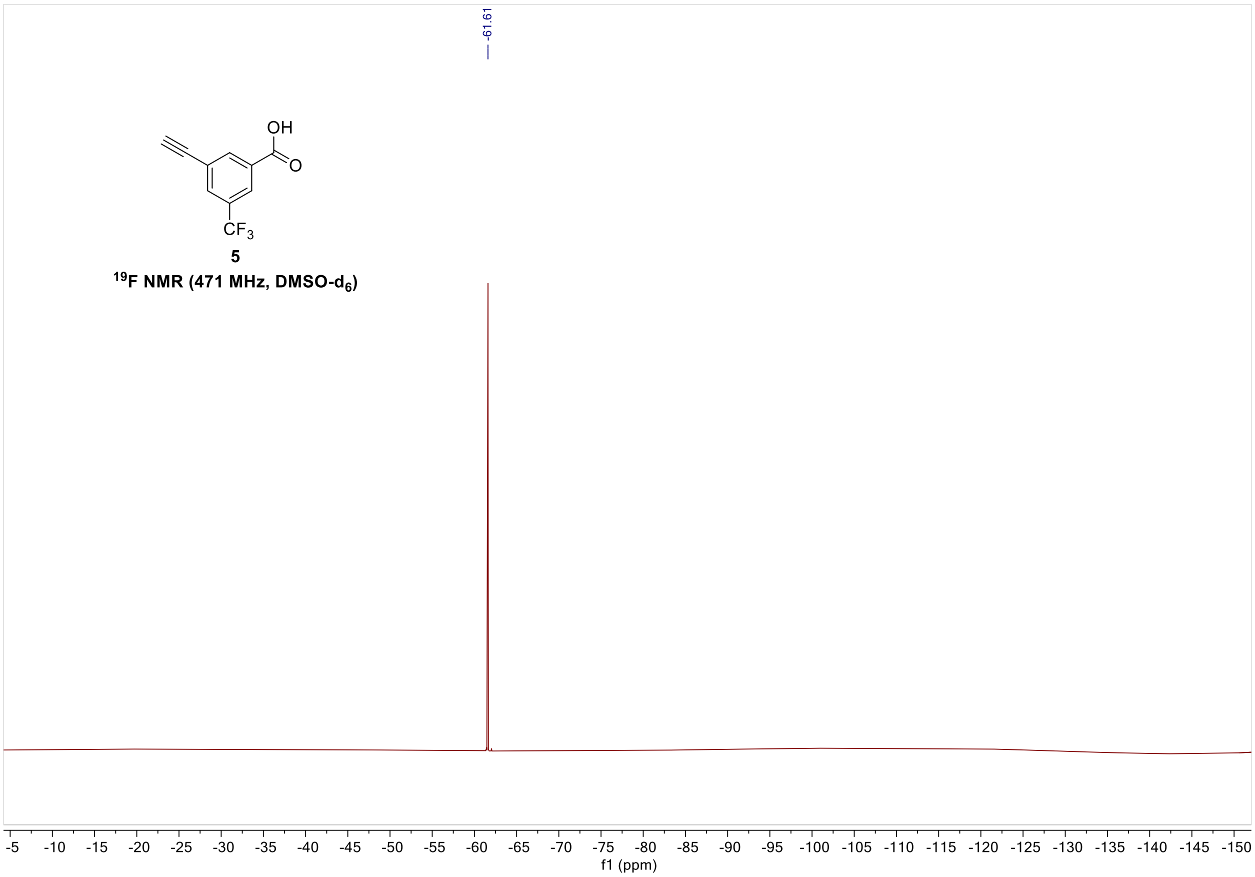
-61.61
OH
O
CF3
5
19F NMR (471 MHz, DMSO-d6)
-5 -10 -15 -20 -25 -30 -35 -40 -45 -50 -55 -60 -65 -70 -75 -80 -85 -90 -95 -100 -105 -110 -115 -120 -125 -130 -135 -140 -145 -150
f1 (ppm)

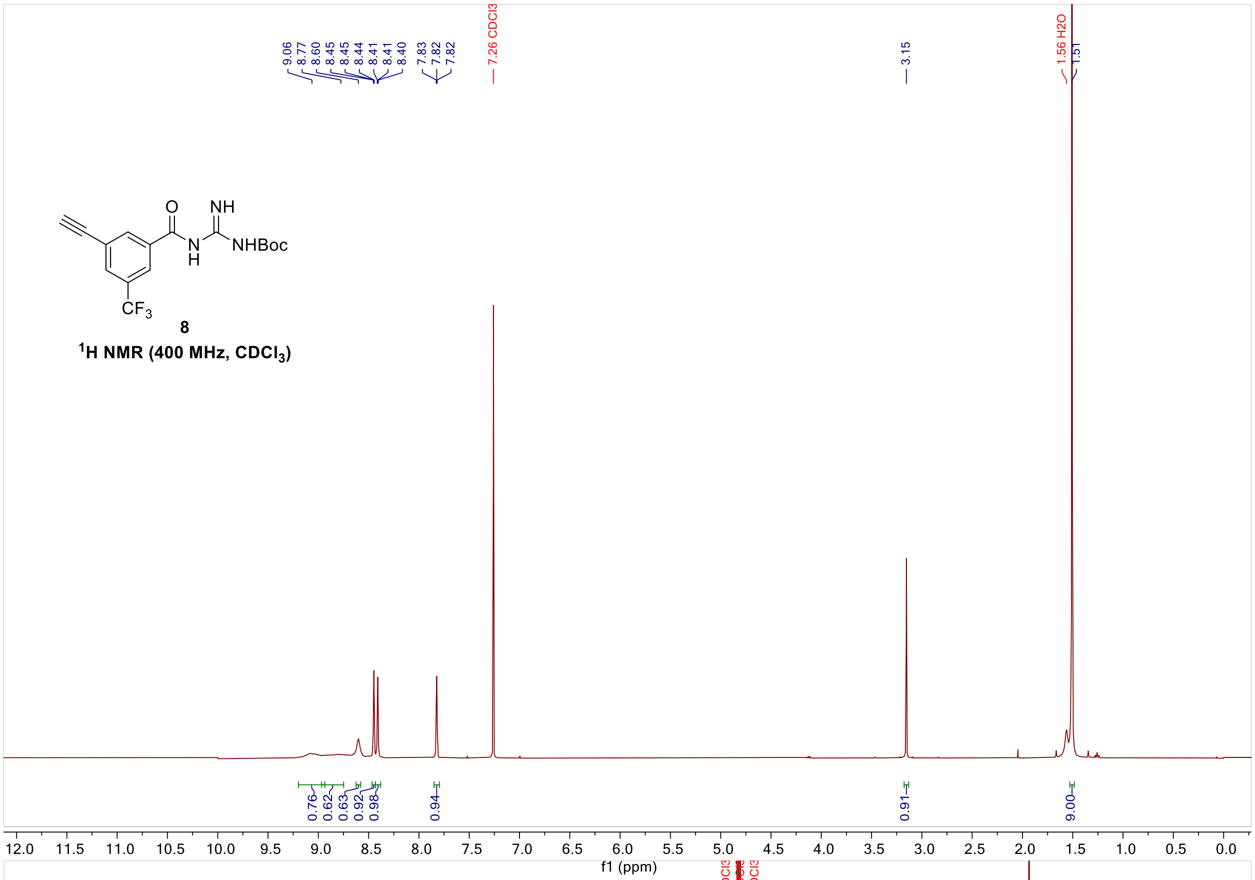
9.06
8.77
8.60
8.45
8.45
8.44
8.41
8.41
8.40
7.83
7.82
7.82
7.26 CDCl3
3.15
1.56 H2O
1.51
O
NH
N
H
NHBoc
CF3
8
1H NMR (400 MHz, CDCl3)
0.76
0.62
0.63
0.92
0.98
0.94
0.91
9.00
12.0 11.5 11.0 10.5 10.0 9.5 9.0 8.5 8.0 7.5 7.0 6.5 6.0 5.5 5.0 4.5 4.0 3.5 3.0 2.5 2.0 1.5 1.0 0.5 0.0
f1 (ppm)

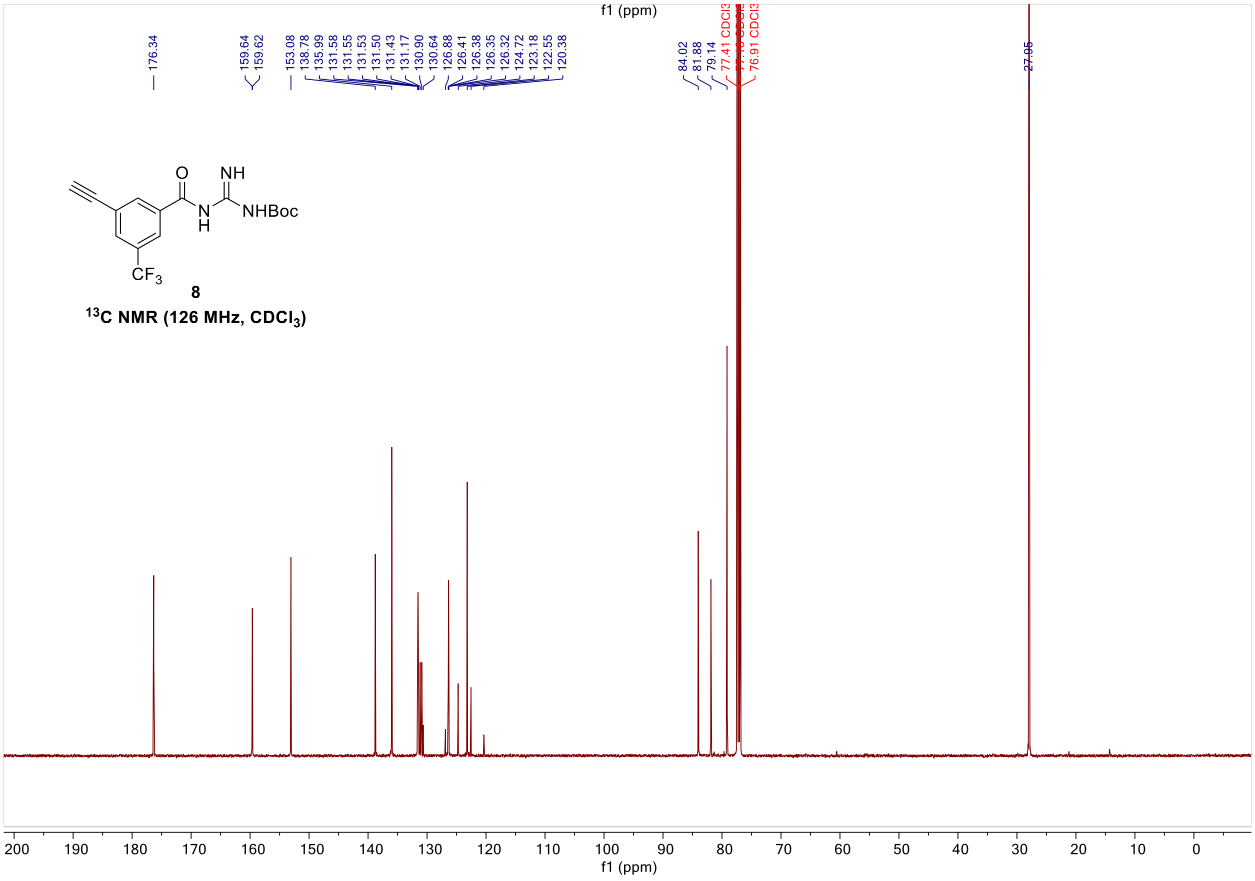
176.34
159.64
159.62
153.08
138.78
135.99
131.58
131.55
131.53
131.50
131.43
131.17
130.90
130.64
126.88
126.41
126.38
126.35
126.32
124.72
123.18
122.55
120.38
84.02
81.88
79.14
77.41 CDCl3
76.91 CDCl3
27.95
O
NH
N
H
NHBoc
CF3
8
13C NMR (126 MHz, CDCl3)
200 190 180 170 160 150 140 130 120 110 100 90 80 70 60 50 40 30 20 10 0
f1 (ppm)

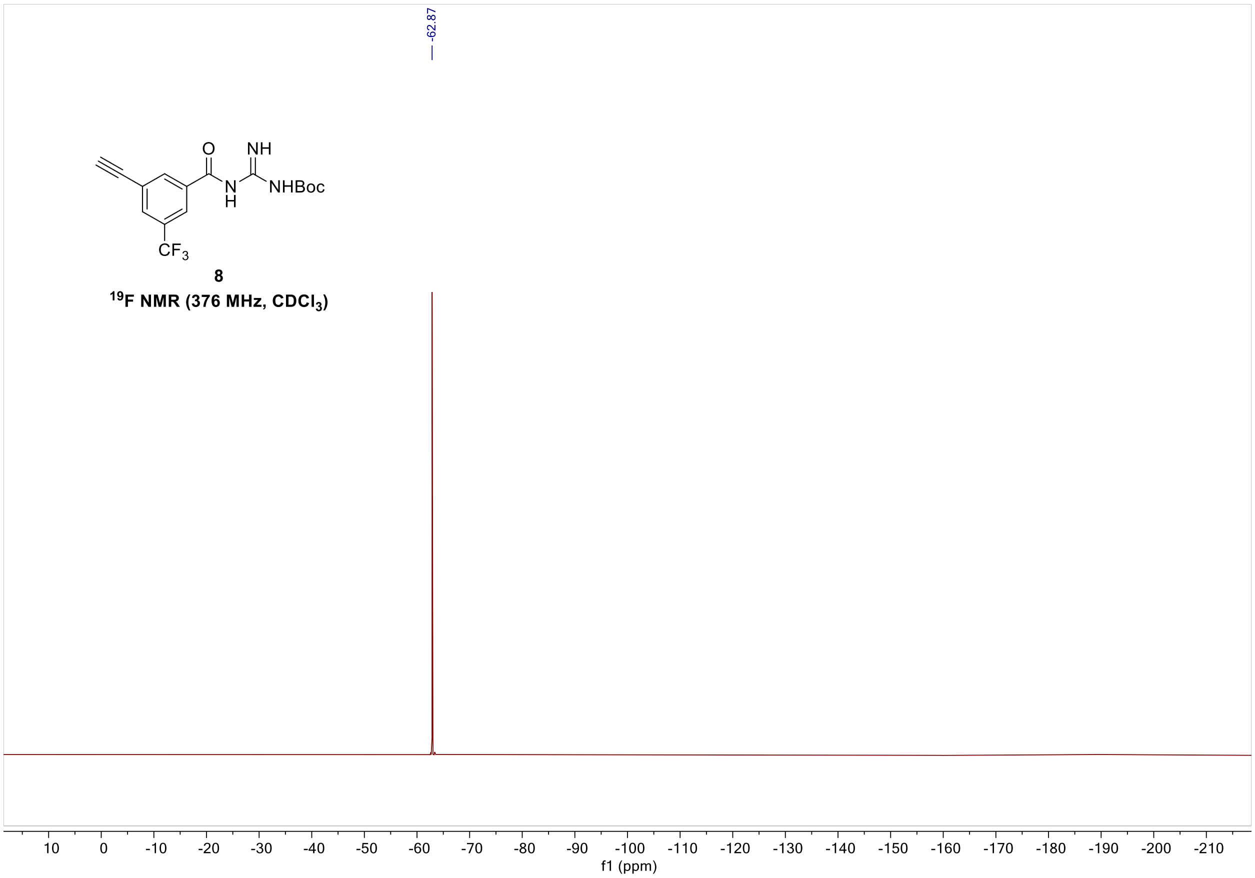
-62.87
NH
O
NHBoc
N
H
CF3
8
19F NMR (376 MHz, CDCl3)
10 0 -10 -20 -30 -40 -50 -60 -70 -80 -90 -100 -110 -120 -130 -140 -150 -160 -170 -180 -190 -200 -210
f1 (ppm)

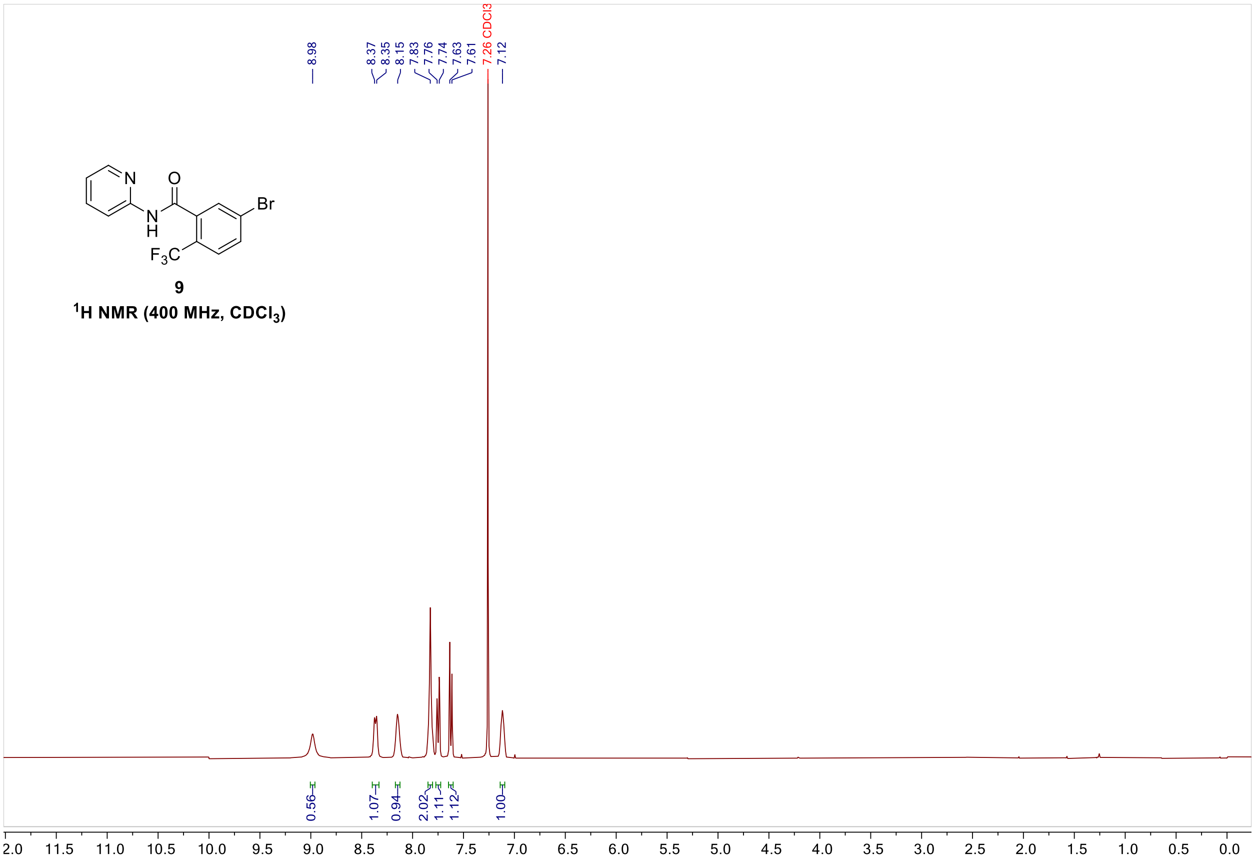
8.98
8.37
8.35
8.15
7.83
7.76
7.74
7.63
7.61
7.26 CDCl3
7.12
Br
N
O
N
H
F3C
9
1H NMR (400 MHz, CDCl3)
0.56
1.07
0.94
2.02
1.11
1.12
1.00
f1 (ppm)

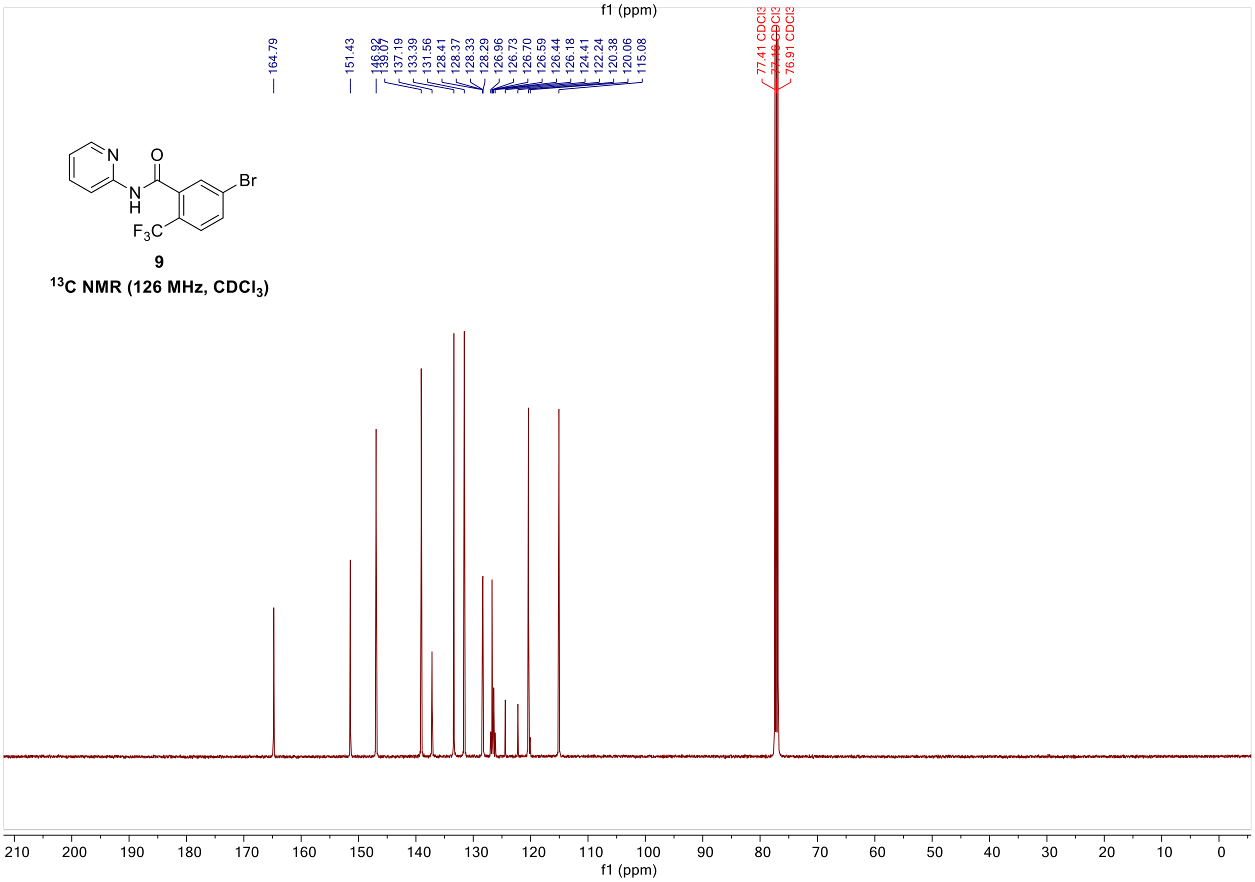
164.79
151.43
137.19
133.39
131.56
128.41
128.37
128.33
128.29
126.96
126.73
126.70
126.59
126.44
126.18
124.41
122.24
120.38
120.06
115.08
77.41 CDCl3
76.91 CDCl3
Br
N
O
N
H
F3C
9
13C NMR (126 MHz, CDCl3)
f1 (ppm)

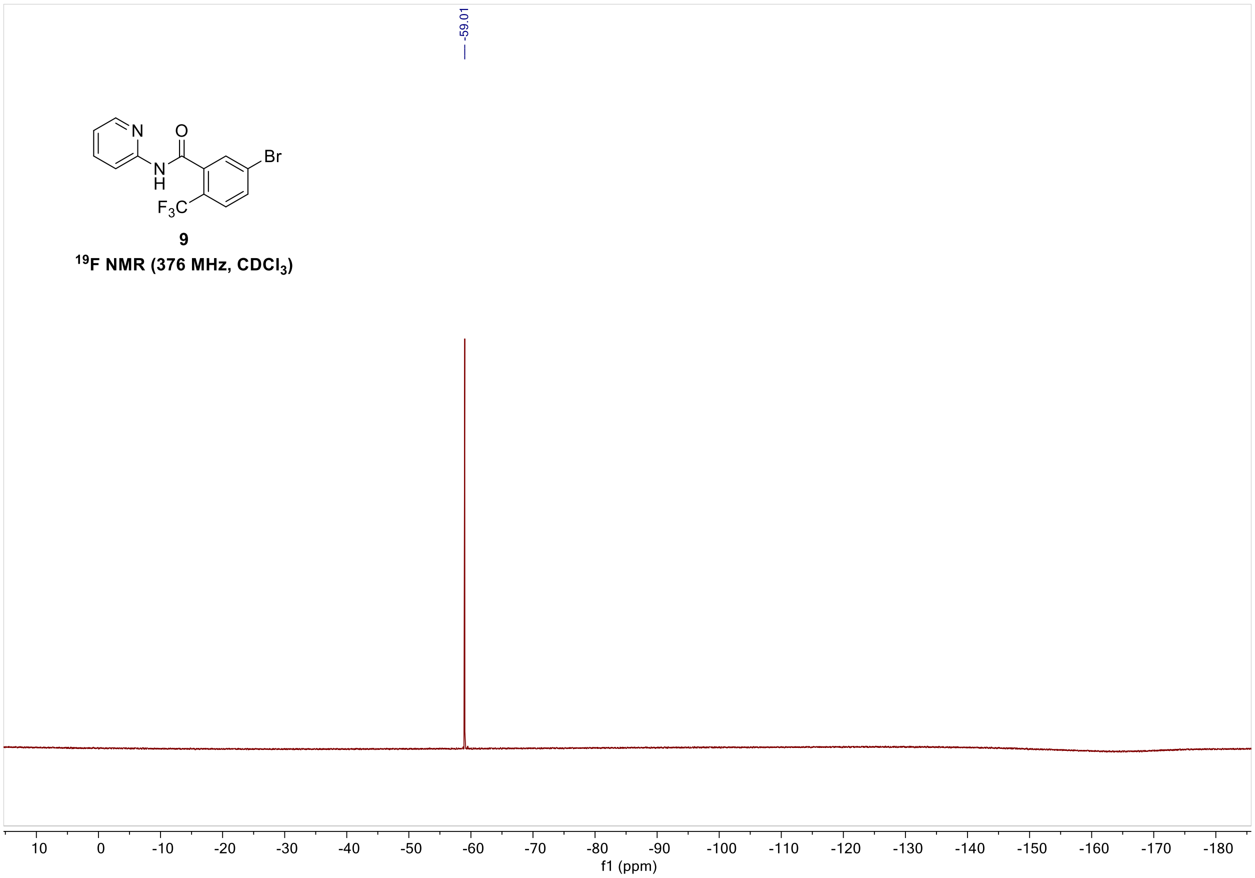
-59.01
N
O
N
H
Br
F3C
9
19F NMR (376 MHz, CDCl3)
10
0
-10
-20
-30
-40
-50
-60
-70
-80
-90
-100
-110
-120
-130
-140
-150
-160
-170
-180
f1 (ppm)

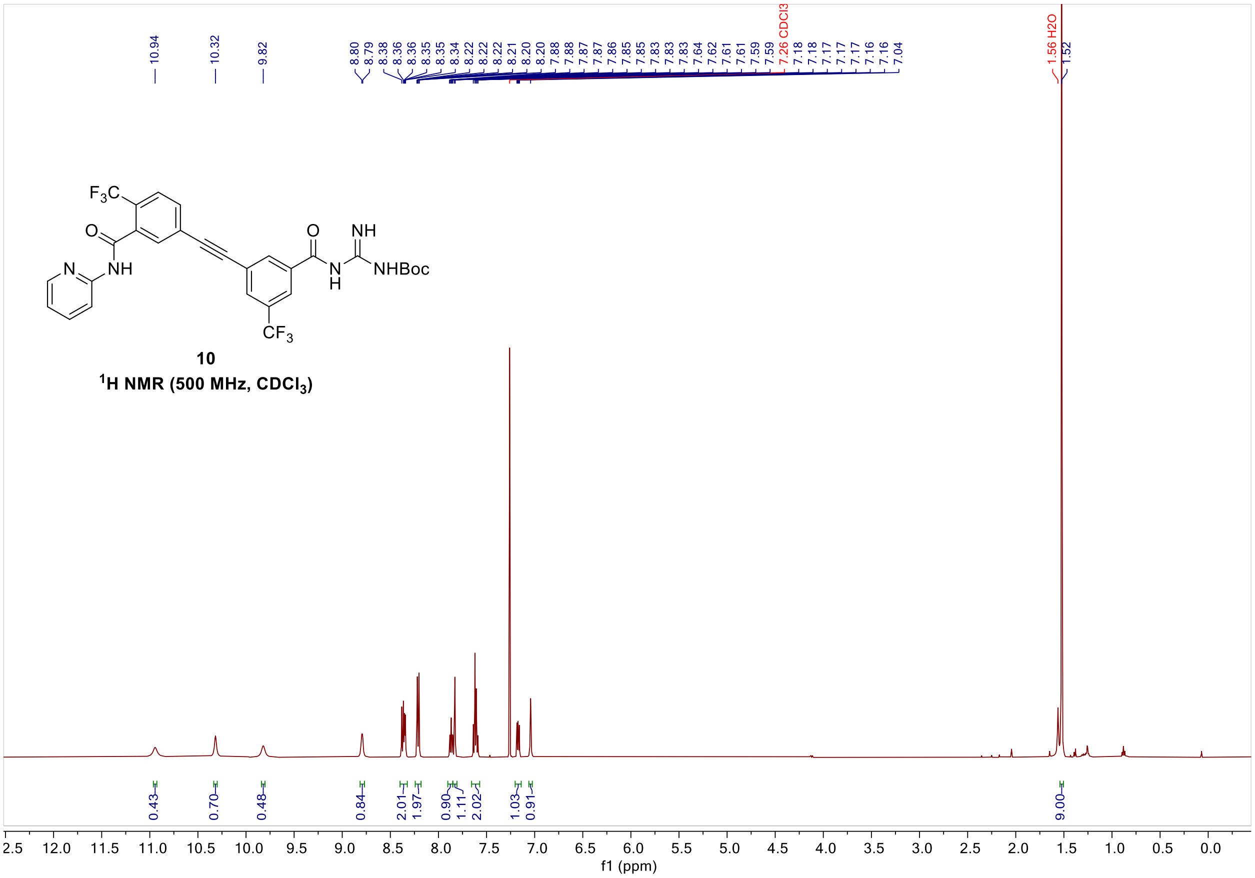
10
1H NMR (500 MHz, CDCl3)
f1 (ppm)

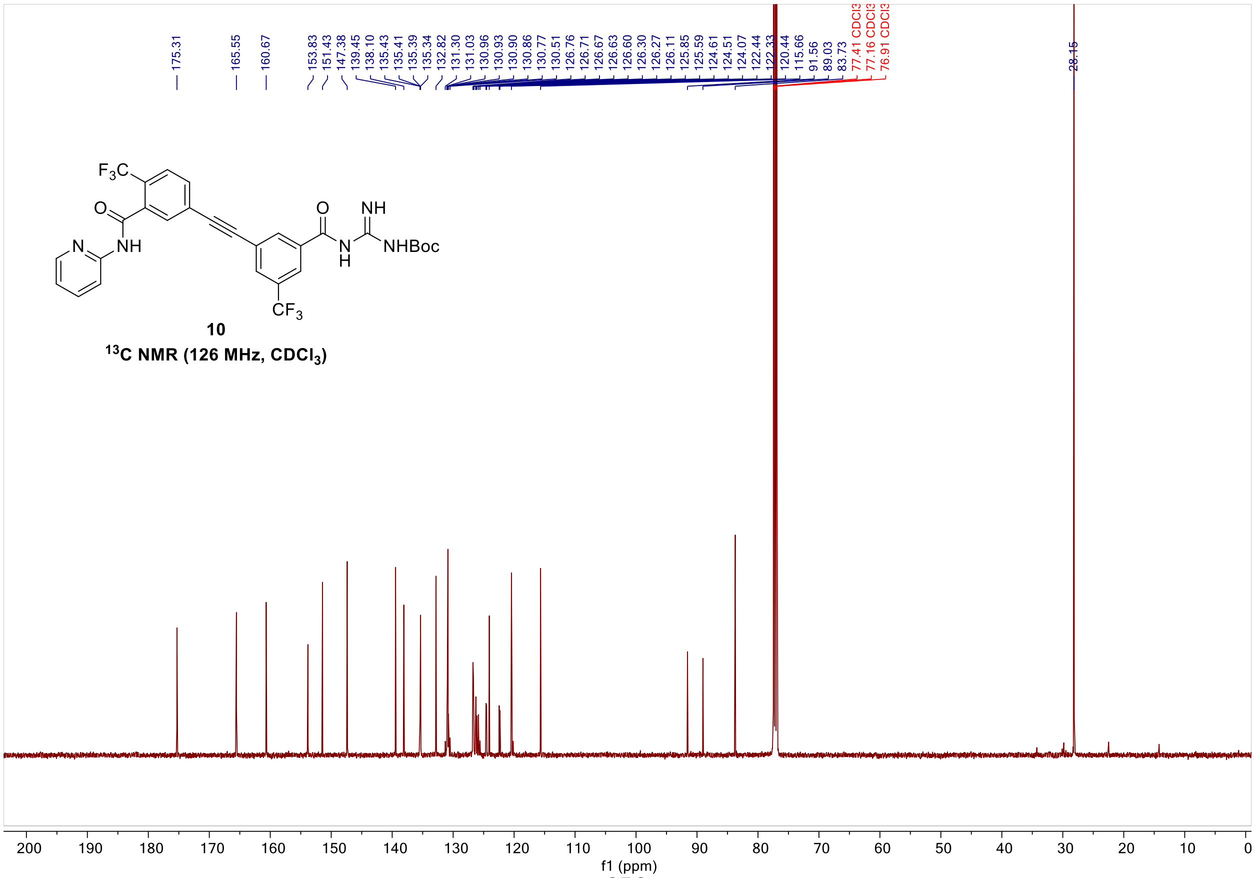
10
13C NMR (126 MHz, CDCl3)
f1 (ppm)

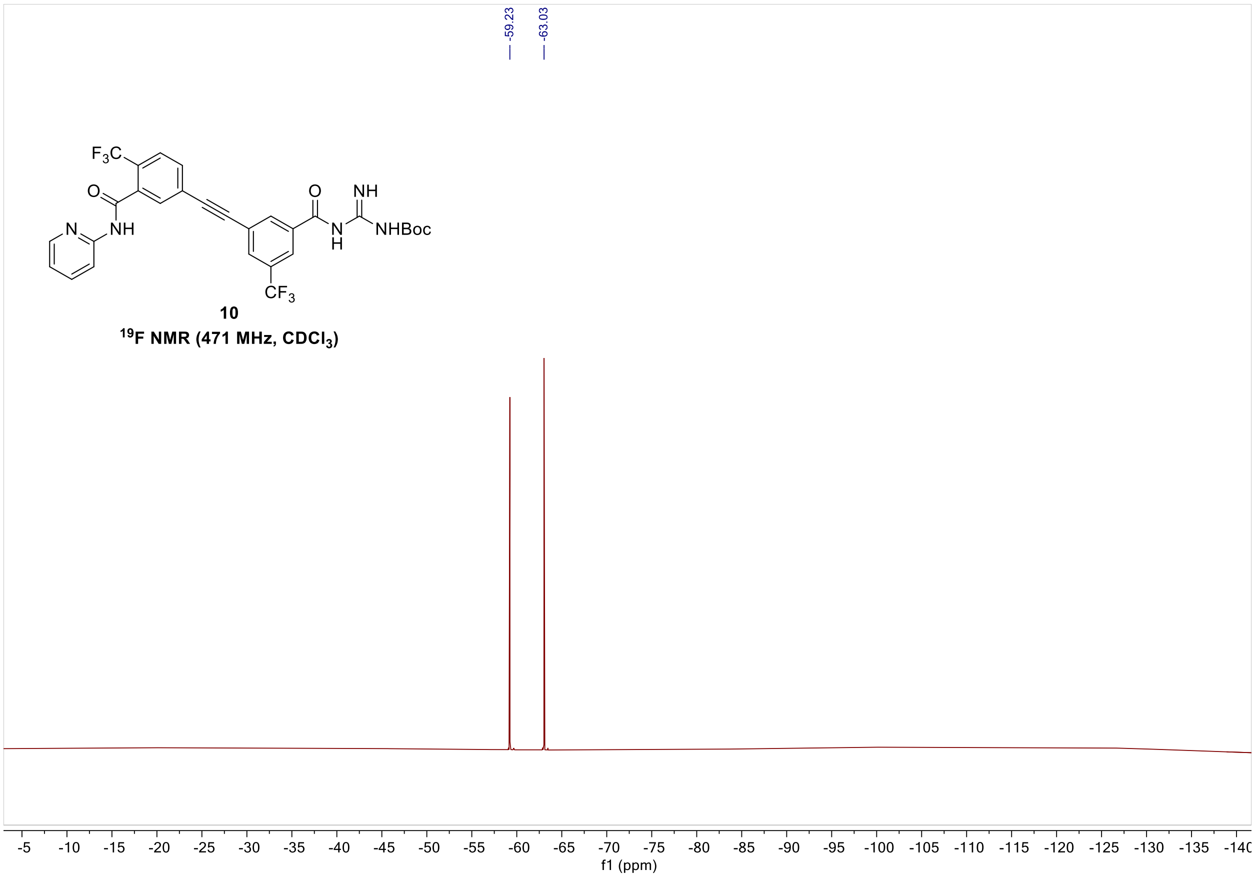
-59.23
-63.03
F3C
O
NH
N
O
NH
N
H
NHBoc
CF3
10
19F NMR (471 MHz, CDCl3)
-5 -10 -15 -20 -25 -30 -35 -40 -45 -50 -55 -60 -65 -70 -75 -80 -85 -90 -95 -100 -105 -110 -115 -120 -125 -130 -135 -140
f1 (ppm)

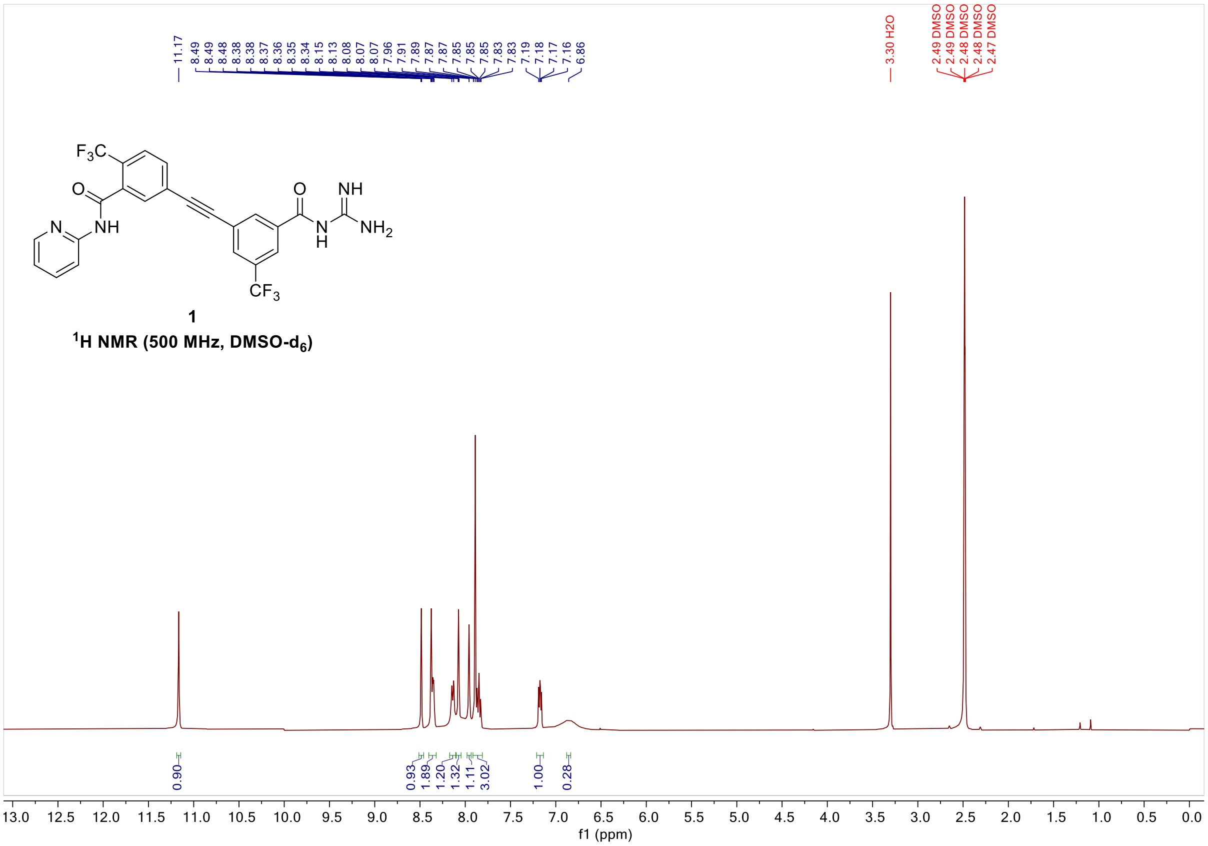

11.17
8.49
8.49
8.48
8.38
8.38
8.37
8.36
8.35
8.34
8.15
8.13
8.08
8.07
8.07
7.96
7.91
7.89
7.87
7.87
7.85
7.85
7.85
7.83
7.83
7.19
7.18
7.17
7.16
6.86
3.30 H2O
2.49 DMSO
2.49 DMSO
2.48 DMSO
2.48 DMSO
2.47 DMSO
F3C
O
NH
N
O
NH
N
H
NH2
CF3
1
1H NMR (500 MHz, DMSO-d6)
0.90
0.93
1.89
1.20
1.32
1.11
3.02
1.00
0.28
13.0
12.5
12.0
11.5
11.0
10.5
10.0
9.5
9.0
8.5
8.0
7.5
7.0
6.5
6.0
5.5
5.0
4.5
4.0
3.5
3.0
2.5
2.0
1.5
1.0
0.5
0.0
f1 (ppm)

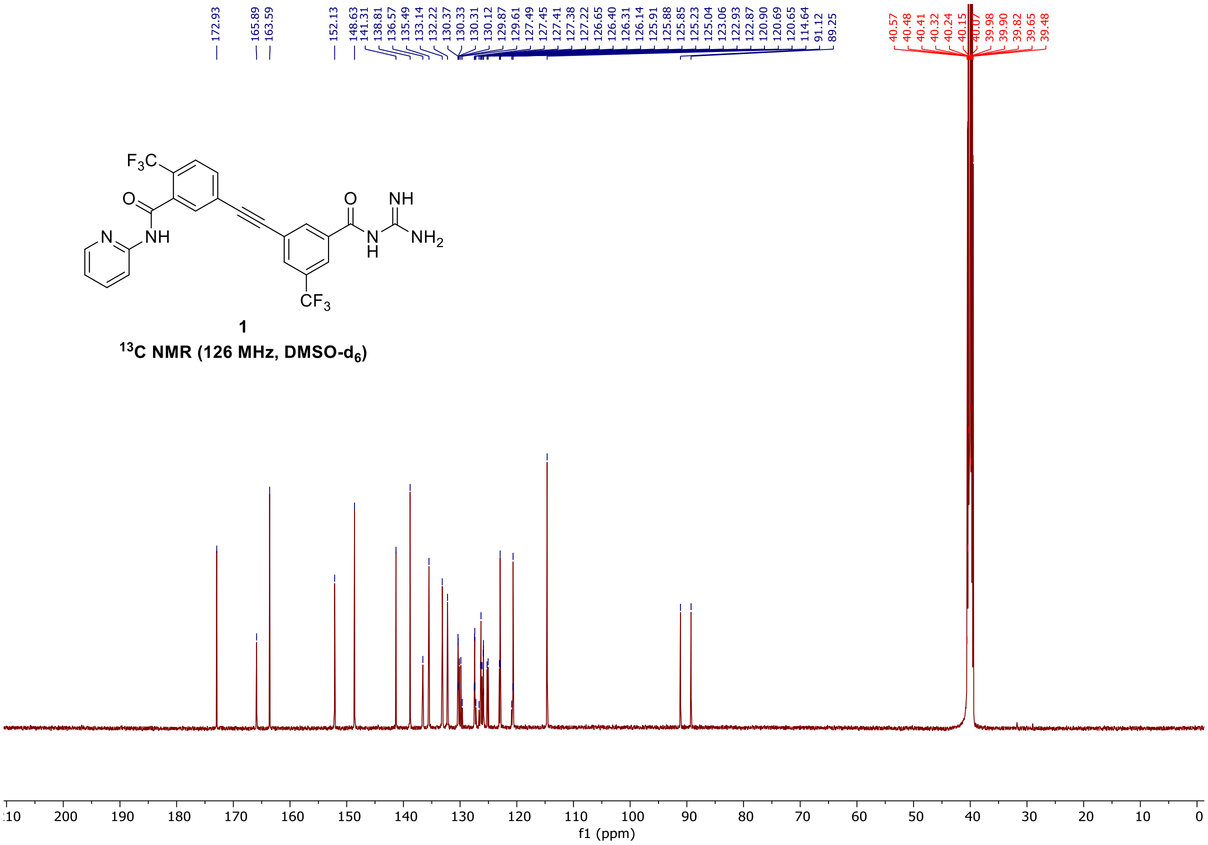

172.93
165.89
163.59
152.13
148.63
141.31
138.81
136.57
135.49
133.14
132.22
130.37
130.33
130.31
130.12
129.87
129.61
127.49
127.45
127.41
127.38
127.22
126.65
126.40
126.31
126.14
125.91
125.88
125.85
125.23
125.04
123.06
122.93
122.87
120.90
120.69
120.65
114.64
91.12
89.25
40.57
40.48
40.41
40.32
40.24
40.15
40.07
39.98
39.90
39.82
39.65
39.48
F3C
O
NH
N
O
NH
N
H
NH2
CF3
1
13C NMR (126 MHz, DMSO-d6)
200
190
180
170
160
150
140
130
120
110
100
90
80
70
60
50
40
30
20
10
0
f1 (ppm)

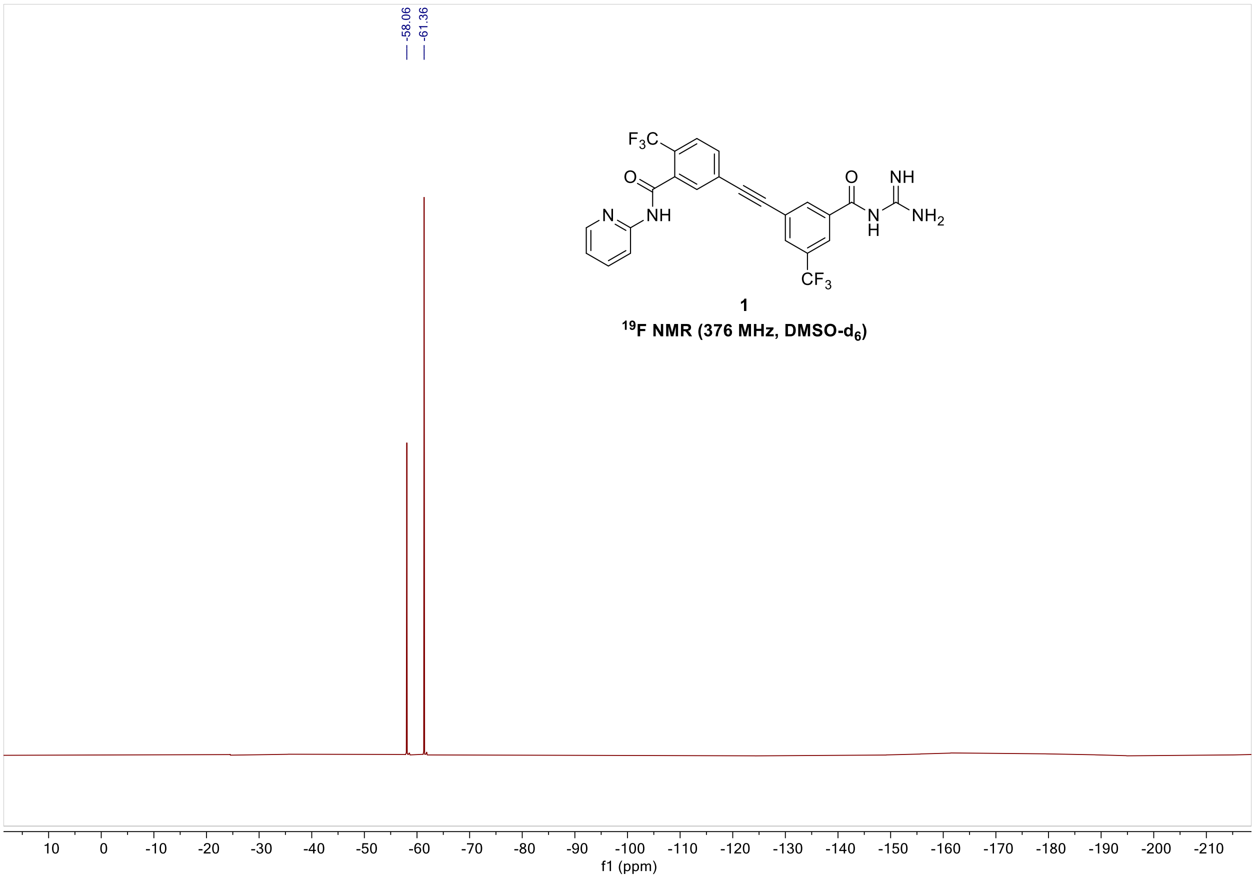

-58.06
-61.36
F3C
O
NH
N
NH
NH2
H
N
CF3
1
19F NMR (376 MHz, DMSO-d6)
10 0 -10 -20 -30 -40 -50 -60 -70 -80 -90 -100 -110 -120 -130 -140 -150 -160 -170 -180 -190 -200 -210
f1 (ppm)
